# Spatial resolution of cellular senescence dynamics in human colorectal liver metastasis

**DOI:** 10.1111/acel.13853

**Published:** 2023-05-08

**Authors:** Ombretta Garbarino, Luca Lambroia, Gianluca Basso, Veronica Marrella, Barbara Franceschini, Cristiana Soldani, Fabio Pasqualini, Desiree Giuliano, Guido Costa, Clelia Peano, Davide Barbarossa, Destro Annarita, Andreina Salvati, Luigi Terracciano, Guido Torzilli, Matteo Donadon, Francesca Faggioli

**Affiliations:** ^1^ IRCCS Humanitas Research Hospital Rozzano, Milan Italy; ^2^ Department of Molecular and Translational Medicine University of Brescia Brescia Italy; ^3^ Institute of Genetics and Biomedical Research, UoS of Milan National Research Council Milan Italy; ^4^ Department of Hepatobiliary and General Surgery IRCCS Humanitas Research Hospital Rozzano, Milan Italy; ^5^ Department of Biomedical Science Humanitas University Pieve Emanuele (MI) Italy; ^6^ Fondazione Human Technopole Milan Italy; ^7^ Department of Pathology IRCCS Humanitas Research Hospital Rozzano, Milan Italy

**Keywords:** cellular senescence, colorectal cancer liver metastasis, EMT, prognostic role, senescence‐associated secretory phenotype, spatial transcriptomics

## Abstract

Hepatic metastasis is a clinical challenge for colorectal cancer (CRC). Senescent cancer cells accumulate in CRC favoring tumor dissemination. Whether this mechanism progresses also in metastasis is unexplored. Here, we integrated spatial transcriptomics, 3D‐microscopy, and multicellular transcriptomics to study the role of cellular senescence in human colorectal liver metastasis (CRLM). We discovered two distinct senescent metastatic cancer cell (SMCC) subtypes, transcriptionally located at the opposite pole of epithelial (e) to mesenchymal (m) transition. SMCCs differ in chemotherapy susceptibility, biological program, and prognostic roles. Mechanistically, epithelial (e)SMCC initiation relies on nucleolar stress, whereby c‐myc dependent oncogene hyperactivation induces ribosomal RPL11 accumulation and DNA damage response. In a 2D pre‐clinical model, we demonstrated that RPL11 co‐localized with HDM2, a p53‐specific ubiquitin ligase, leading to senescence activation in (e)SMCCs. On the contrary, mesenchymal (m)SMCCs undergo TGFβ paracrine activation of NOX4‐p15 effectors. SMCCs display opposing effects also in the immune regulation of neighboring cells, establishing an immunosuppressive environment or leading to an active immune workflow. Both SMCC signatures are predictive biomarkers whose unbalanced ratio determined the clinical outcome in CRLM and CRC patients. Altogether, we provide a comprehensive new understanding of the role of SMCCs in CRLM and highlight their potential as new therapeutic targets to limit CRLM progression.

## Graphical abstract

Two distinct senescent cancer ecosystems located at the opposite pole of epithelial (e) to mesenchymal (m) transition duel in metastasis onset of colorectal cancer, defining the immune landscape and the clinical fate of patients.

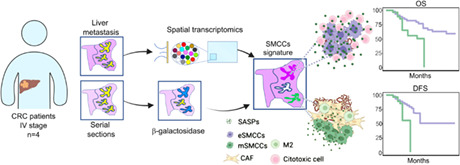

**Abbreviations**
CRC: colorectal cancer
CRLM: colorectal liver metastasis
CSs: capture spots
DFS: disease free survival
ECM: extracellular matrix
EMT: epithelial to mesenchymal transition
(e)SMCC: epithelial SMCC
H&E: hematoxylin and eosin
HR: hazard ratio
iSMCC: intermediate SMCC
(m)SMCC: mesenchymal SMCC
OS: overall survival
SA‐β‐gal: senescence associated β ‐galactosidase
SMCC: senescent metastatic cancer cell
ST: spatial transcriptomics

## 1 INTRODUCTION

Metastasis is an inefficient process because of the multitude of obstacles that circulating cancer cells meet before successful seeding (Celià‐Terrassa & Kang, 2018). Indeed, in a mouse model of melanoma, <0.1% of tumor cells were capable of metastasizing (Luzzi et al., 1998). However, 90% of cancer lethality is due to metastasis (Steeg, 2006). Colorectal liver metastasis (CRLM) is not an exception. CRLM develops in 50% of colorectal cancer (CRC) patients. Surgical resection after neo‐adjuvant chemotherapy is the gold standard, but only 10%–20% of patients are eligible (Zhou et al., 2022). CRLM seeding was reported to occur in 80% of CRC cases before the primary tumor was clinically detectable (Hu et al., 2019), indicating that metastasis initiation is an unexpectedly early event.

Cellular senescence is viewed as a desirable outcome for cancer therapy because it represents a state of permanent cell‐cycle arrest (Hayflick & Moorhead, 1961; Sieben et al., 2018). Indeed, in this terminal state, cancer cells are still alive but are either not harmful or in an intermediate stage of vulnerability and targetable by senolytic drugs (Naylor et al., 2013; Ovadya & Krizhanovsky, 2018). Senescent cancer cells might also promote immune recruitment for their suicide or signal for the clearance of non‐senescent cancer cells through their secretome (Kang et al., 2011; Xue et al., 2007). However, there is evidence also of a cancer‐promoting function, including local invasion and epithelial‐to‐mesenchymal transition (EMT), fueling efforts in targeting senescent cells therapeutically (Ou et al., 2021). To date, translation of pro‐senescence compounds to the clinic has focused on primary tumors (Ovadya & Krizhanovsky, 2018) with less attention given to the role of cellular senescence in metastasis. Additionally, studies on chemotherapy‐naive metastatic patients are rare, except Haugstetter et al. (2010), who described senescence as a common feature in CRLM and having a positive prognostic role in patient survival. By contrast, senescent cancer cells were found to support tumor spread in colorectal and thyroid cancers (Choi et al., 2021; Kim et al., 2017). These conflicting findings highlight the complex role of cellular senescence in the evolution of cancer from primary to secondary organs (Faggioli et al., 2023).

Here, to study the pathophysiological role of cellular senescence in CRLM, we combined spatial transcriptomics (ST) with the senescence‐associated β‐galactosidase (SA‐β‐gal) assay and 3D‐microscopy. We define the mechanisms of senescence activation and the role played by chemotherapy. Moreover, integration of data into a multicellular transcriptional database identified the senescent‐dependent cancer ecosystems that coexist within CRC and CRLM and that determine the clinical outcome.

## 2 RESULTS

### 2.1 Tumor cells consistently turn senescent in CRLM

First, we analyzed a small cohort of CRLM patients (Table S1) with the SA‐β‐gal assay, the gold standard for the detection of pan‐senescence (Debacq‐Chainiaux et al., 2009; Gorgoulis et al., 2019). 75% of specimens were SA‐β‐gal‐positive in intrametastatic areas (Figure S1a,b). Cell‐cycle arrest was confirmed by immunohistochemistry for the CDK2 inhibitor p21^Cip1^ and the CDK4/6 inhibitor p16^Ink4a^, the molecular effectors of the p53‐ and pRB‐sustained senescence pathways (Gorgoulis et al., 2019). As for primary CRC (Choi et al., 2021), SA‐β‐gal‐positive cells accumulated prevalently at the metastatic marginal zone (Figure S1a). The average SA‐β‐gal‐positive area within cancer spanned from 10% to 30% (Figure S1c). No significant correlation was found between clinical parameters and senescence grading (data not shown). However, we confirmed in an independent cohort that cellular senescence is not a stochastic event in CRLM.

### 2.2 Deciphering the transcriptional heterogeneity of senescent metastatic cancer cells by generating a whole‐transcriptome spatial atlas of human CRLM

**FIGURE 1:**
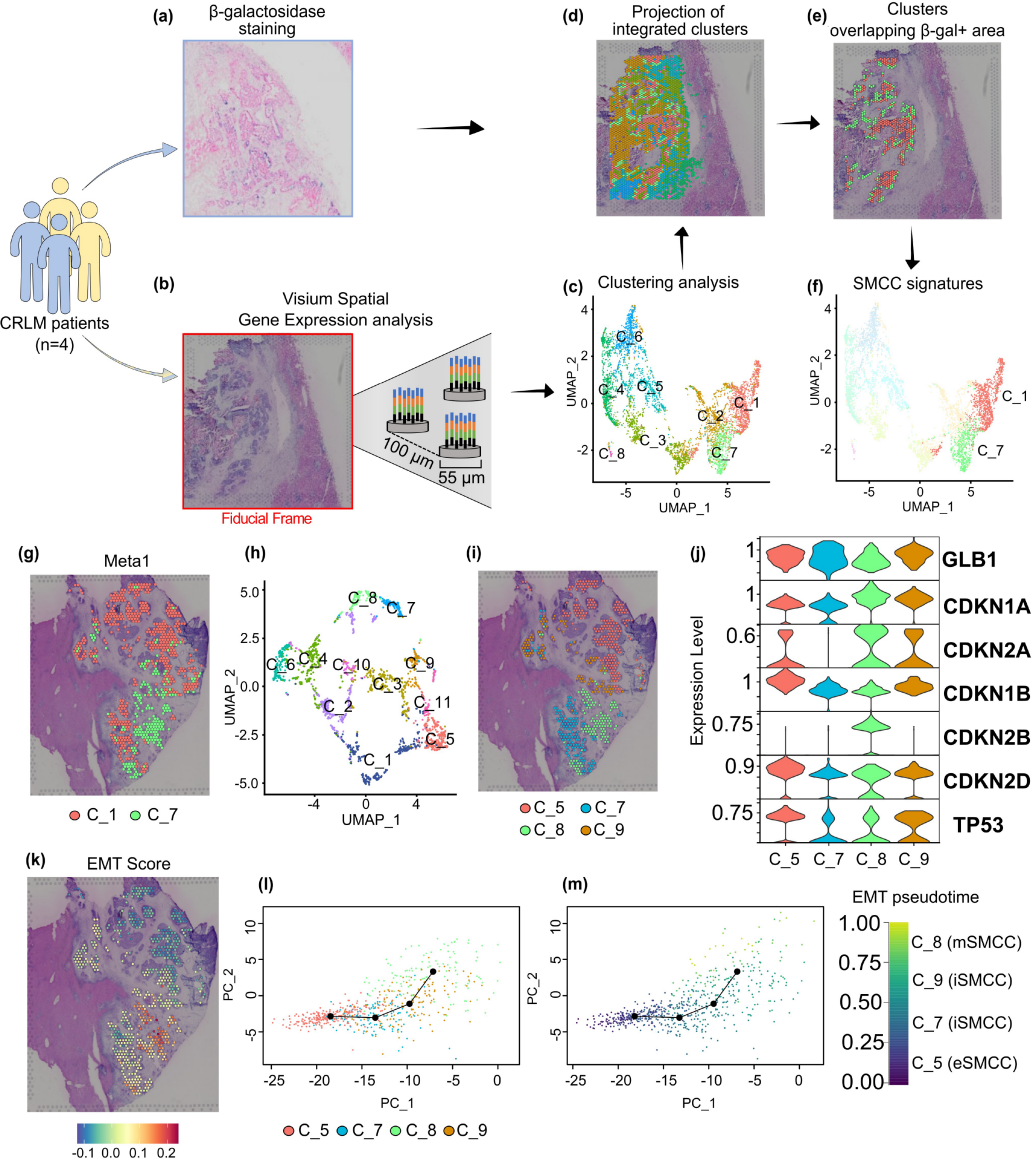
Spatial and transcriptional identification of senescent metastatic cancer cells in colorectal liver metastasis (CRLM). (a–f) Workflow of spatial transcriptomics combined with SA‐β‐gal assay on CRLM specimens. Four liver metastatic biopsies were analyzed with ST technology: Two of them (blue) underwent SA‐β‐gal assay. (a) A SA‐β‐gal‐positive specimen. (b) Hematoxylin and eosin (H&E) image of a CRLM specimen prior to permeabilization for ST processing. (c) UMAP of ST‐integrated analysis. (d) Projection of 8 integrated cancer clusters onto the H&E image acquired before permeabilization. (e) SA‐β‐gal‐positive transcriptional signature projected onto an H&E image. (f) UMAP of two identified senescent cancer clusters: C_1 (salmon) and C_7 (green). (g) Spatial projection of tumoral senescent clusters on meta1. (h) UMAP of individual analysis, showing 11 clusters. (i) Spatial projection of senescent clusters onto the corresponding H&E image. (j) Violin plots of senescence‐associated genes in tumoral clusters for the metal specimen. (k) Epithelial‐to‐mesenchymal transition (EMT) score generated on the EMTome platform. (l) Trajectory generated by PCA on metal genes. (m) Pseudotime trajectory on EMT genes.

We then asked whether it would be possible to dissect the role of cellular senescence within CRC metastasis. We reasoned that any transcriptional approach would not, per se, accurately identify senescent metastatic cancer cells (SMCCs), which lack exclusive markers (Gorgoulis et al., 2019), and that intra‐tumor heterogeneity would make deciphering the senescence signature even more difficult. To overcome these limits, we performed ST and SA‐β‐gal assay on serial cryosections (Figure 1a,b). This approach allowed us to localize β‐gal‐positive cells within metastases and then to identify overlapping transcriptional clusters through ST on the consecutive section. The data were collected from four post‐chemotherapy patients; for each, one hepatic lesion was selected and processed through the Visium Spatial Gene Expression protocol (Figure 1b–d); two metastases were pre‐screened with SA‐β‐gal (Figure 1a and Figure S1d–f). To distinguish senescent cells from not senescent, we first compared the histology of the lesion that underwent ST and the consecutive section stained for β‐gal. An expert pathologist, blinded to transcriptional data, identified on ST histological section the areas corresponding to β‐gal‐positive cells (Figure 1a,b). Then, we identified the transcriptional clusters enriched in β‐gal – senescent‐like areas (Figure 1e). ST reliably quantified the spatial distribution of >800 genes in individual capture spots (CSs), adding up to 8431 genes/CS over the four biopsies. We detected 4296.25 ± 1106 unique molecular identifiers and 1489 ± 226 unique genes per CS. Integrated gene‐expression clustering of the intrametastatic datasets identified eight distinct clusters in 17,874 genes stratified in tumoral crypt and extracellular matrix (ECM) subgroups (Figure 1c). Spatial data were verified to strongly recapitulate CRLM histology (Figure S2a–c). Parenchymal data were not included in further analysis.

Within the transcriptional space, we uncovered two consistently expressed transcriptional programs (C_1 and C_7), later referred to as “senescent metastatic cancer cells” (SMCCs) (Figure 1f). Notably, only in one sample (referred to as meta1) C_1 and C_7 clusters were uniformly located in geographically distinct regions (Figure 1g); in the other three, the clusters were distributed unevenly, like mixed puzzle pieces, making it difficult to address how the cells behaved (Figure S2d).

We, therefore, performed unsupervised individual analysis on the meta1 specimen to get a more accurate gene‐expression profile of 16,811 genes, clustered in 11 subgroups (Figure 1h), identifying four distinct transcriptional signatures in the tumoral crypts (C_5, C_7–C_9) overlapping with SMCCs clusters (Figure 1i). All four clusters expressed high levels of galactosidase (GLB1) mRNA (Figure 1j), confirming that even if meta1 was not pre‐screened for β‐gal activity, active GLB1 transcription proved that the SMCCs were bona fide (Hernandez‐Segura et al., 2017). In line with the literature (Kumari & Jat, 2021; Sonzogni et al., 2014), there was an accumulation of p21^Cip1^ and p16^Ink4b^ cyclin inhibitor kinases and p19^Ink2d^, which is involved in senescence related‐chromatin compaction, suggesting that redundant mechanisms coexist to reinforce proliferation arrest (Figure 1j). Notably, the cyclin kinase inhibitors p27^Kip1^ (Lloyd et al., 1999) and p15^Ink4b^ (Hannon & Beach, 1994) were differentially expressed in C_5 and C_8, raising the question of whether the properties of these SMCCs subtypes were fundamentally different.

### 2.3 SMCCs have EMT‐dependent transcriptional programs and different secretomes

Epithelial‐to‐mesenchymal transition is a reversible process implicated in invasion, tumor stemness, and metastatic spread (Kalluri & Weinberg, 2009). To investigate whether SMCC signatures differed in EMT states, we integrated our spatial data into the EMTome database, which includes an EMT signature of 1153 genes across 32 cancer types (Vasaikar et al., 2021). We assigned an EMT score to each of our CSs based on the expression values of the EMT gene signature (Figure 1k). EMT score mapping revealed that C_8 was enriched in mesenchymal markers, while C_5 was closely related to the epithelial signature. This was confirmed by principal component analysis (PCA) derived trajectories of EMT genes, with C_5 located at the zero EMT state and C_8 at the trajectory extreme, representing a mature mesenchymal phenotype (Figure 1l,m). As a result, we defined C_5 and C_8 as epithelial (e) and mesenchymal (m) SMCCs, respectively. The regulon‐enriched analysis also supported these findings (Table S2). C_7 and C_9 signatures were found to be in between the EMT poles and were thus classified as intermediate stages (iSMCCs, Figure 1m, EMT pseudotime).

**FIGURE 2:**
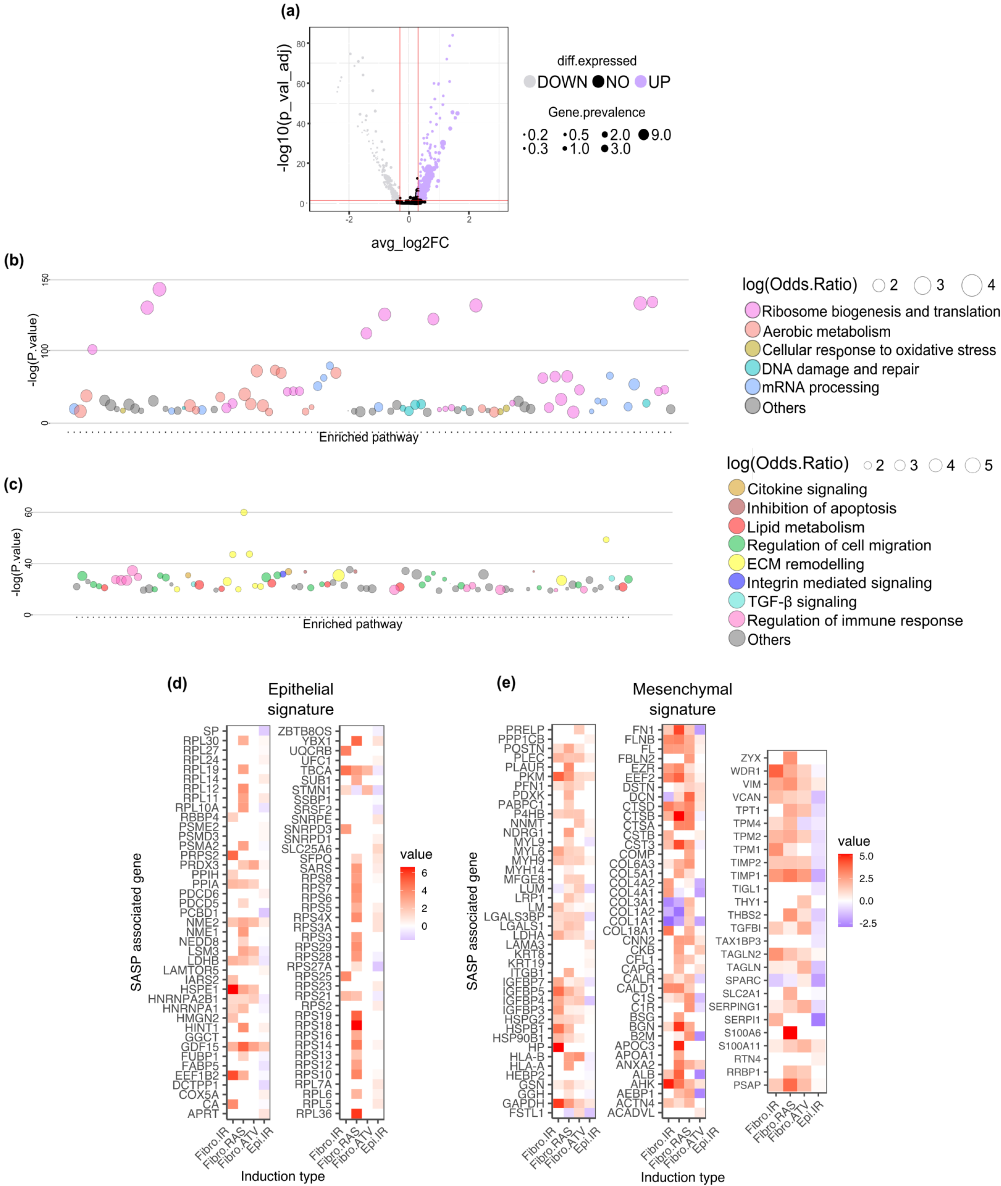
eSMCCs and mSMCCs have independent transcriptional programs. (a) Volcano plot of differential expression analysis of eSMCC vs. mSMCCs. Average log2 FC >0.3; adjusted *p* < 0.05. Gene ontology analysis and most‐significant pathways are given for eSMCC (b) and mSMCC (c) clusters. Heatmaps of SASP‐associated genes for eSMCC (d) and mSMCC (e) signatures, compared with the SASP atlas database.

Expression analysis of ST data of eSMCCs versus mSMCCs identified 349 differentially upregulated genes (Figure 2a and Table S3). Gene ontology analysis of eSMCC‐related genes identified enrichment of pathways involved in nucleolar stress (Figure 2b and Table S4) (Boulon et al., 2010; Yang et al., 2018). In contrast, the 229 differentially upregulated genes identified in mSMCCs were enriched for tumor carcinogenesis and micro‐environment remodeling (Figure 2c and Table S5), suggesting that mSMCCs undergo functional and structural reprogramming typical of aggressive cancer cells.

Many factors can influence the senescence program. Here, we investigated the SMCCs phenotype related to stress signals and the type of cells, exploiting the Senescence‐Associated Secretory Phenotype (SASP) atlas. This comprehensive proteomic database includes the profile of senescent pulmonary fibroblasts and epithelial cells developed from several stressors (Basisty et al., 2020). 24% of eSMCCs genes overlapped with those in the SASP database, while up to 51% of mSMCCs genes were included (Figure 2d,e). These findings indicate SMCCs diverged in terms of the type and magnitude of secretory molecules. Of note, the eSMCC SASP profile recapitulated the signature of RAS oncogene‐induced senescent fibroblasts, while fewer similarities emerged in comparison with epithelial irradiated senescent cells (Figure 2d). Additionally, mSMCCs had a more mixed phenotype similar to several inducers, including pharmacological treatment (Figure 2e), suggesting that for SMCCs SASPs complexity was determined by the senescence‐stressor inducer.

Mechanistically, SASPs were supported by ubiquitous and SMCC‐type related signaling driving upregulation of NFKB1, the master regulator of primarily SASP factors, in both SMCC subtypes (Figure S3a,b) (Salminen et al., 2012).

### 2.4 Mechanisms of SMCCs induction

#### 2.4.1 Nucleolar stress drives the eSMCC signature

**FIGURE 3:**
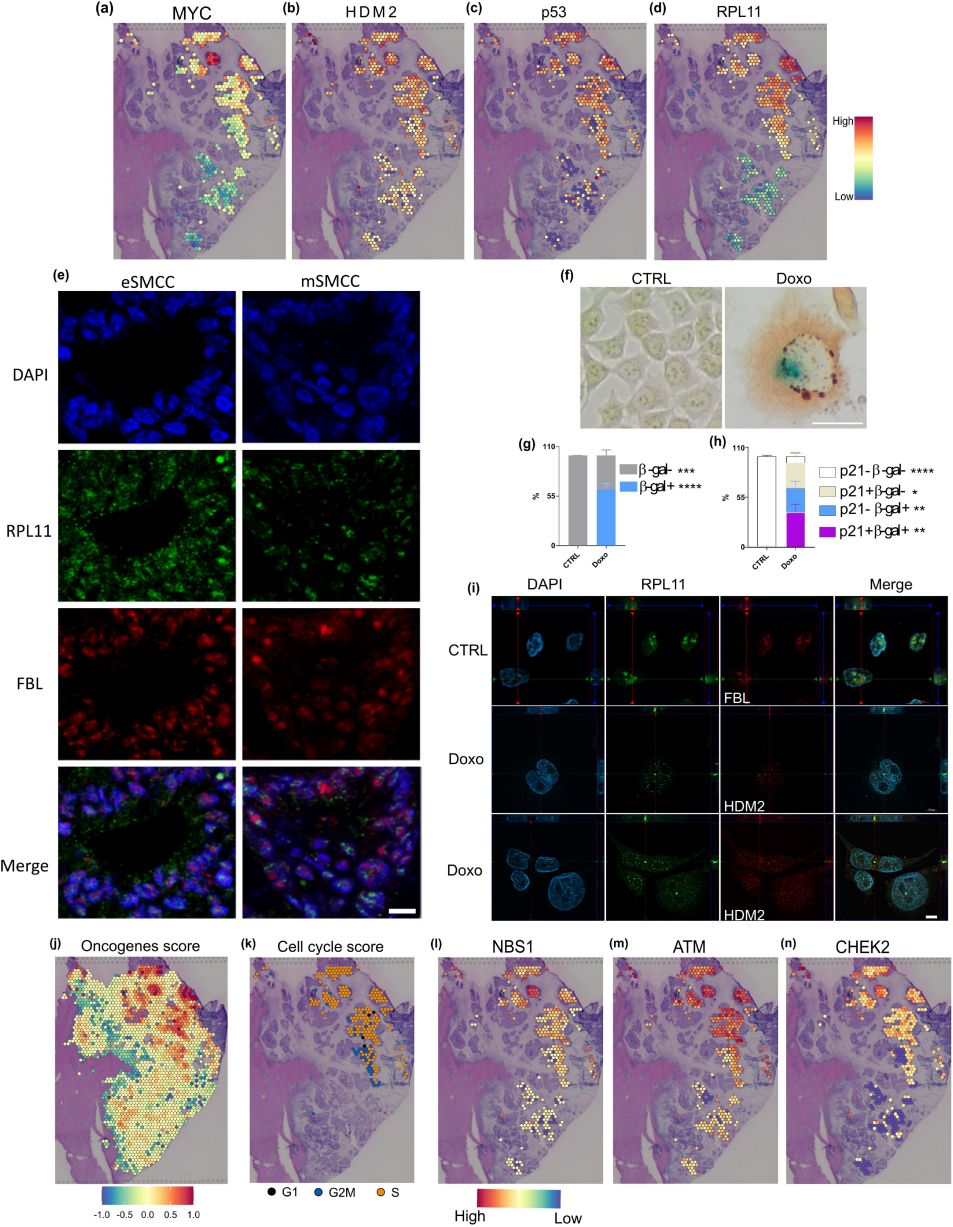
Nucleolar stress drives the eSMCC signature. (a–d) Activity maps of genes involved in HDM2‐p53 axis. (e) Z‐stack projection of RPL11 and FBL immunofluorescence on selected areas of eSMMCs and mSMMCs on meta1, bar 10 μm. (f) Co‐staining of p21 protein (brown) and β‐gal enzymatic activity (blue) on HCT‐116 cell line (CTRL vs. Doxo), bar 100 μm. Quantification of β‐gal (g) and p21‐positive cells (h) scored by optical evaluation over three experiments. *T* test, *p < 0.05; **p < 0.01; ***p < 0.001; ****p < 0.0001. (i) Representative single focal plane of Z‐stacks for RPL11, FBL, and HDM2 immunofluorescence on doxorubicin‐exposed HCT‐116 cells and control, bar 20 μm. (j) Oncogene activation score. (k) Cell cycle phase analysis. (l) DNA damage sensor, upstream (m) and downstream (n) kinase activity maps.

Nucleolar stress indicates an impairment of morphological and functional nuclear homeostasis, which lead to the activation of p53 effector (though inhibition of E3 ubiquitin‐protein ligase HDM2) and, more often, cellular senescence. Ribosome biogenesis and its transcriptional controller, *c‐MYC* (Destefanis et al., 2020), are key regulators of nucleolar integrity and are extensively exploited by eSMCCs as *HDM2* and p53 (Figure 3a–d). To validate involvement in anti‐cancer response, we focused on the highly expressed ribosomal protein RPL11 (Figure 3d), which inhibits p53–HDM2 interaction (Havel et al., 2015; Lohrum et al., 2000). First, through 3‐multilabel immunofluorescence on whole sections of meta1, we confirmed that RPL11 accumulated significantly in eSMCCs (Figure 3e). Confocal analysis revealed that when located in the cytoplasm, RPL11 probably takes part in global translation typical of excessive ribosome biogenesis, but when in the nucleoplasm (77% over 181 inspected cells), it interacts with HDM2 (Russo & Russo, 2017). HDM2's activity map overlapped with cells having nucleoplasm‐located RPL11 suggesting possible interaction (Figure 3b,e). To validate this hypothesis, we induced cellular senescence in the HCT‐116 cell line, in which both p16^Ink4a^ alleles are inactivated (Burri et al., 2001) and HDM2‐p53‐p21^Cip1^‐dependent senescence most likely occurs. Doxorubicin (Doxo) is a known chemotherapic agent but also a potent genotoxic ribosomal stress inducer (Morgado‐Palacin et al., 2014; Petrova et al., 2016; Was et al., 2018). After its exposure, upregulation of p21^Cip1^ at the protein level confirmed the activation of a p53‐dependent senescence phenotype in HCT‐116 treated cells, reaching a mean of 62% β‐gal‐positive cells (Figure 3f–h). Interestingly, not all β‐gal‐positive cells expressed the p21 protein, most likely due to a delay in the senescence activation program. Interestingly, the transcriptional signature of Doxo‐treated HCT‐116 cells overlapped with both eSMCC and mSMCC groups, supporting our previous observation that the type of stressor rather than the cell type is a determinant in SMCCs phenotype (Figure S3c–f). Indeed, Doxo as a chemotherapic agent and nucleolar stress inducer is able to activate both phenotypes in the same cell line. Immunofluorescence for RPL11 and HDM2 revealed that RPL11 co‐localized with fibrillarin in the basal condition, but that the nucleolar marker was not more present in treated cells, indicative of nucleolar stress (Figure 3i and data not shown). Given the nucleolar disruption, it was not possible to assess the translocation of RPL11 into the nucleoplasm; however, RPL11 and HDM2 were tightly co‐localized within the nucleus in >72% of inspected cells; in contrast, 50% of cytoplasmic RPL11 co‐localized with HDM2 (Figure 3i), supporting the role of RPL11 as a shuttle for HDM2‐mediated proteasomal degradation (Bursać et al., 2012). Therefore, we speculated that excessive free RPL11 in eSMCCs increased the chance of interaction with HDM2 at the nuclear and cytoplasmic levels, driving stabilization of p53 and activation of senescence.

We then reasoned that c‐Myc was a proto‐oncogene that was not only involved in ribosome biogenesis; indeed, activated oncogenes are prominent inducers of senescence (Braig & Schmitt, 2006). Therefore, we pulled together the expression data of significantly upregulated oncogenes in eSMCCs and generated an oncogene activation score for the epithelial signature, indicative of high mitogenic activity (Figure 3j). As expected, we found genes (e.g., *Braf*, *Fos*) involved in conflicting roles as a tumor promoters or suppressor in various cancers (Mahner et al., 2008; Michaloglou et al., 2008). Excessive mitogenic stimulation was confirmed by the *CellCycleScoring* function (Seurat) of R package, indicative of eSMCCs being synchronized in the S phase (Figure 3k) (Tirosh et al., 2016). This condition was not exclusive to proliferating cells, since oncogene‐induced senescent cancer cells can be S‐phase arrested (Campisi & D'Adda Di Fagagna, 2007).

The limit on oncogene‐induced cell growth is aberrant DNA replication, which in turn generates DNA damage (Campisi & D'Adda Di Fagagna, 2007). Indeed, ST analysis on eSMCCs identified upregulation of molecular factors involved in each step of the DNA damage repair pathway: sensors of single‐ and double‐strand breaks (e.g., NBS1), central regulators of the DNA damage response network (e.g., ATM; Maréchal & Zou, 2013) and downstream kinases (e.g., CHEK2; Figure 3l–n and data not shown). The functional activation of DNA damage repair pathway was confirmed by the co‐localization of the phosphorylated form of H2AX and ATR in eSMCC crypts detected by immunofluorescence (Figure S3g; Podhorecka et al., 2010). Thus, oncogene overexpression in eSMCCs overlaps with DNA damage response activation, and most likely contributes to the establishment of senescence.

#### 2.4.2 TGFβ‐dependent paracrine activation of mSMCC cell cycle arrest

**FIGURE 4:**
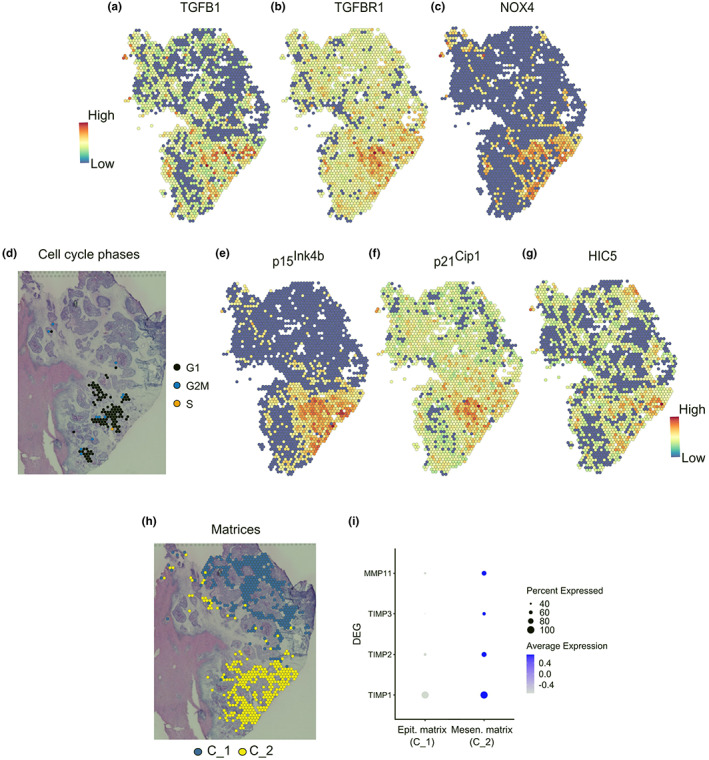
Paracrine activation of the mSMCC signature. (a) Activity maps for TGFβ and (b) its cognate receptor, TGFB1. (c) ST distribution of NOX4. (d) Cell‐cycle assignment of mSMCCs. Activity maps for p15^Ink4b^ (e), p21^Cip1^ (f) and HIC5 (g) are shown. (h) Extracellular matrix cell cluster localization over meta1 (C1, C2). (i) Differential remodeling factors in extracellular matrix surrounding crypts.

Pleiotropic TGFβ can exert cell‐autonomous tumor suppressor or pro‐tumorigenic effects in cancer (Baba et al., 2022; Massagué, 2008). To understand the multiple consequences of TGFβ activity within mSMCCs, we first confirmed local activation. In meta1, TGFβ1 mainly localized to stroma adjacent to mesenchymal crypts, a finding suggesting that this cytokine is produced, or at least retained, in a latent state by ECM cells (Figure 4a). The TGFBr1 receptor was selectively overexpressed in mSMCCs and corresponding ECM cells (Figure 4b), which were most likely the main targets of TGFβ (vander Ark et al., 2018). Then, to define the impact of TGFβ on SMCCs, we focused on its known anti‐proliferative properties. Indeed, TGFβ inhibits proliferation by inducing G1‐ phase cell‐cycle arrest through the activation of NADPH oxidase 4 (*NOX4*) and accumulation of reactive oxygen species, which drive c‐MYC downregulation. In turn, c‐MYC negatively regulates p15^Ink4b^ and p21^Cip1^ expression (Baba et al., 2022; Bird et al., 2018; Senturk et al., 2010; Seoane & Gomis, 2017). In line with the literature, we found positive transcriptional activity for *NOX4* in mSMCCs (Figure 4c), downregulation of *c‐MYC* (Figure 3a), G1 phase synchronized cancer cells (Figure 4d), and upregulation of the CDK inhibitory genes p15^Ink4b^ and p21^Cip1^ (Figure 4e,f); the upregulation of hydrogen peroxide‐inducible clone 5 (*HIC5*) (Figure 4g), a sensor of free radicals and an antagonist of *NOX4* (Desai et al., 2014; Wolfgang Doppler, 2019), confirmed the tissue response to *NOX4* activation. Notably, *NOX4* as a downstream effector of TGFβ1 might coordinate additional functions in mSMCCs not related to cell‐cycle arrest, like EMT, tissue remodeling, and angiogenesis (Chen et al., 2017). To this end, we investigated differences in ECM remodeling factors. As expected, epithelial (C1) and mesenchymal (C2) matrices had opposite profiles (Figure 4h,i): mesenchymal ECM was characterized by higher expression of metalloproteinases (*MMP9*, *MMP2*, *MMP11*) and the corresponding inhibitors (*TIMP1*, *TIMP2*, *TIMP3*). These differences reflected distinct abilities to manipulate the micro‐environment and were indicative of effective ECM remodeling in proximity of mesenchymal crypts. All of these features are known to be involved in metastatic spread and poor prognosis (Winkler et al., 2020).

### 2.5 Clinical predictions based on the assessment of epithelial and mesenchymal SMCCs in a CRLM validation cohort and an extended CRC database

**FIGURE 5:**
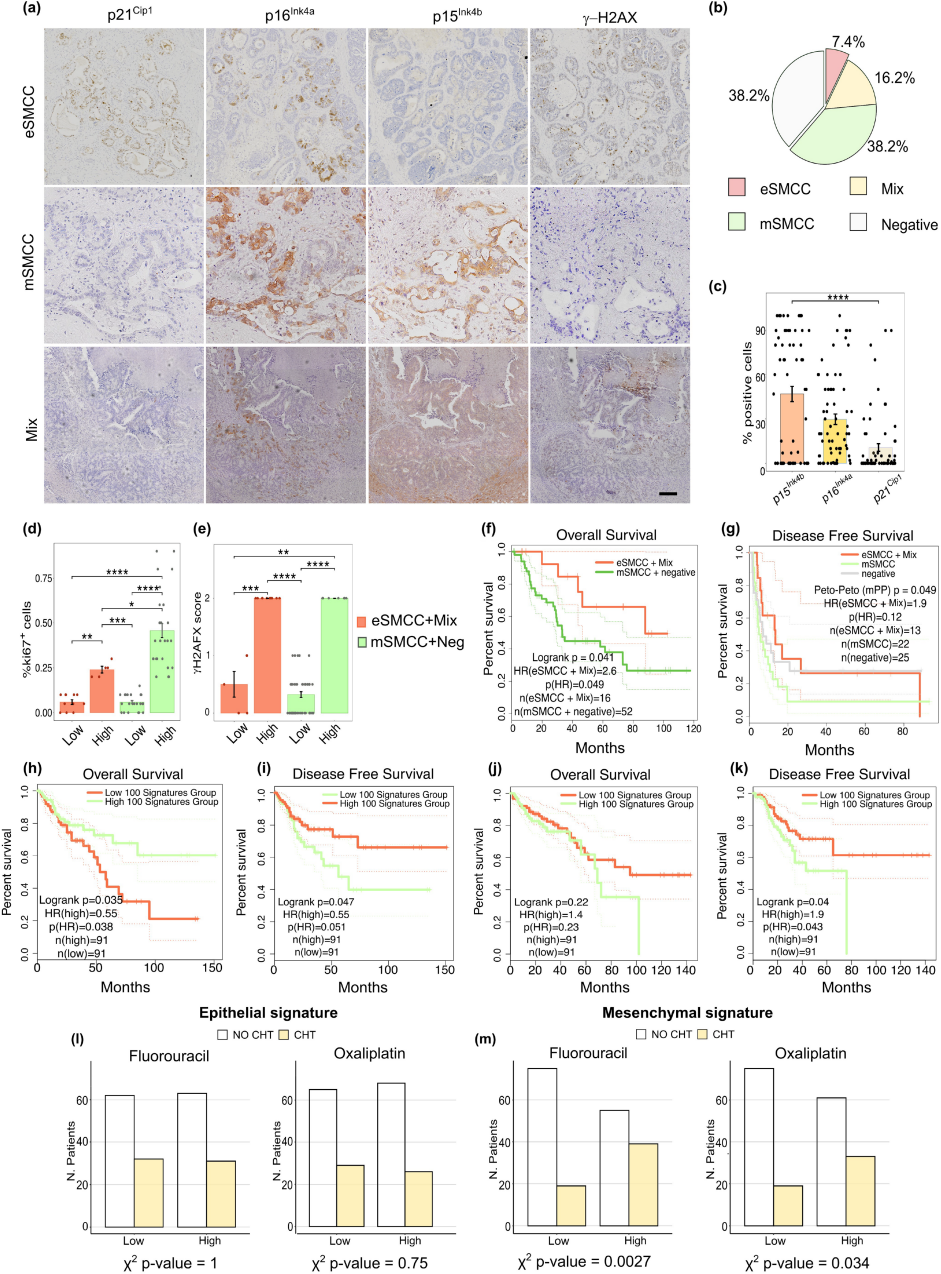
Clinical predictions based on the assessment of epithelial and mesenchymal senescent metastatic cancer cell (SMCC) signatures in colorectal liver metastasis (CRLM) and TCGA‐CRC cohorts. (a) 5‐multilabel immunohistochemistry for p21^Cip1^, p16^Ink4a^, p15^Ink4b^, γH2A.X, and ki67 performed over 68 CRLM patients, randomized in chemo and not chemo treated, bar 200 μm. (b) Overall quantification in SMCC subgroups of the CRLM cohort. (c) Quantification of p21^Cip1^ and p16^Ink4a^, p15^Ink4b^ markers. Wilcox.test, ****p < 0.0001 (d) ki67 and (e) γH2A.X quantification over the SMCCs groups. Wilcox.test, *p < 0.05; **p < 0.01; ***p < 0.001. (f) Overall survival (OS) Kaplan–Meier curve of CRLM patients: peto‐peto, *p < 0.05. (g) Disease‐free survival (DFS) Meier curve relative to the same CRLM cohort. OS Kaplan–Meier curve (h) and DFS‐Meier curve (i) for the eSMCC signature. (j) OS and (k) DFS Kaplan–Meier curves for mSMCCs signature over TCGA‐CRC cohort (*n* = 362). Significance of chemotherapy treatment for eSMCC^high‐low^ (l) and mSMCC^high‐low^ (m) signatures related to fluorouracil and oxaliplatin‐specific drugs.

To investigate the clinical impacts of the eSMCC and mSMMC signatures, we first performed a retrospective analysis (follow‐up, ≥10 years) on CRLM samples randomized into chemotherapy‐naïve (*n* = 34) and treated (*n* = 34) groups (Table S1). To identify patients expressing an SMCC‐type signature, we established a 5‐multilabel serial approach using selected markers of proliferation (ki67), DNA damage (γH2AX), and cell‐cycle arrest (p21^Cip1^, p16^Ink4a^, p15^Ink4b^) that had emerged from our previous spatial analysis (Figures 1j and 5a). We classified the 68 patients into four categories following these criteria: eSMCCs were defined by co‐expression of p16^Ink4a^, p21^Cip1^, and γH2AX (7.4%); when also p15^Ink4b^ was co‐detected, we classified the samples as “Mix” (16.2%); mSMCCs were defined by the co‐expression of p15^Ink4b^ with p16^Ink4a^ and or with p21^Cip1^ (38.2%); all the other possibilities were classified as negative (38.2%). The overall quantification of SMCCs and cell cycle inhibitors markers are indicated in Figure 5b,c. With this approach, we spatially dissected the localization of SMCC populations across the metastasis, identifying within the Mix group three cases (4.4%) in which SMCCs were located in two distinct regions (Figure S4a). Contrarily to meta1, we found Mix type on one side and (e) or (m) SMCCs markers on an adjacent area. eSMCC and Mix‐associated phenotypes were characterized by a low proliferative index (<15% in the metastatic area) and significant phosphorylation of the histone variant H2AX compared to mSMCC and negative groups (Figure 5d,e).

Kaplan–Meier curves showed that eSMCC‐Mix patients had improved overall survival versus the non‐expressing group (Figure 5f), indicating this phenotype's beneficial role in limiting cancer progression. Notably, the eSMCC‐Mix signatures also significantly impacted the risk of relapse (Figure 5g). In DFS, negative and mSMCCs groups acted distinctly, suggesting that mSMCC‐like markers had the worst survival and a principal role in recurrence. Depending on the heterogeneity of therapeutic tools and the limited number of cases randomized in subgroups, we could not establish an impact of chemotherapy in CRLM samples.

To overcome those limits, we investigated the predictive role of the SMCC signatures on The Cancer Genome Atlas database, over an extended group of 362 colon and rectal adenocarcinoma cases. Based on eSMCC versus mSMCC DEG, we selected the genes with *p*‐value adj <0.05. After that, for both e/mSMCC groups, we selected the top one hundred genes according to log2FC. We confirmed a significant association between the eSMCC^high^ signature and improved survival in CRC patients (Figure 5h): indeed, >60% of eSMCC^high^ patients survived more than 150 months; in contrast, only 20% of patients presenting with an eSMCC^low^ signature reached this goal (hazard ratio [HR] = 0.55). Furthermore, the disease‐free survival curve indicated that eSMCC^high^ patients had less than a 50% chance of cancer recurrence (Figure 5i; HR = 0.55, *p* = 0.051). These findings supported the notion of a beneficial role for the eSMCC^high^ signature in primary and corresponding metastatic tumors, as well as its validity as a prognostic marker.

In contrast, the mSMCC^high^ signature was predictive of a worse prognosis for CRC: overall survival indicated that mSMCC^high^ patients were 40% more likely to die within the first 8 years versus mSMCC^low^ patients (Figure 5j; HR = 1.4, pHR = 0.23). These patients had almost twice the chance of cancer relapse versus mSMCC^low^ patients (Figure 5k; HR = 1.9, pHR = 0.043). These findings underscored the poor prognostic value of the mSMCC signature. Of note, there was no correlation between the eSMCC signature and the type of chemotherapy (Figure 5l). Instead, for the mSMCC signature, there was a significant correlation with pharmacological treatment: oxaliplatin and fluorouracil were determinants in the establishment of the highest mSMCC expression (Figure 5m).

Finally, we identified SMCCs in a demonstrative ST analysis of human CRC available at 10× Genomic's website. This analysis demonstrated the reproducibility of our approach over independent experiments, confirming the existence of SMCC populations in primary CRC. At least another tumoral cluster (indicated as not‐SMCCs) was identified without any significant prognostic value (Figure S5a,c), as for iSMCCs signature over TCGA database (data not shown).

### 2.6 Opposing carcinoma immune ecosystems develop from epithelial and mesenchymal *SMCCs*

**FIGURE 6:**
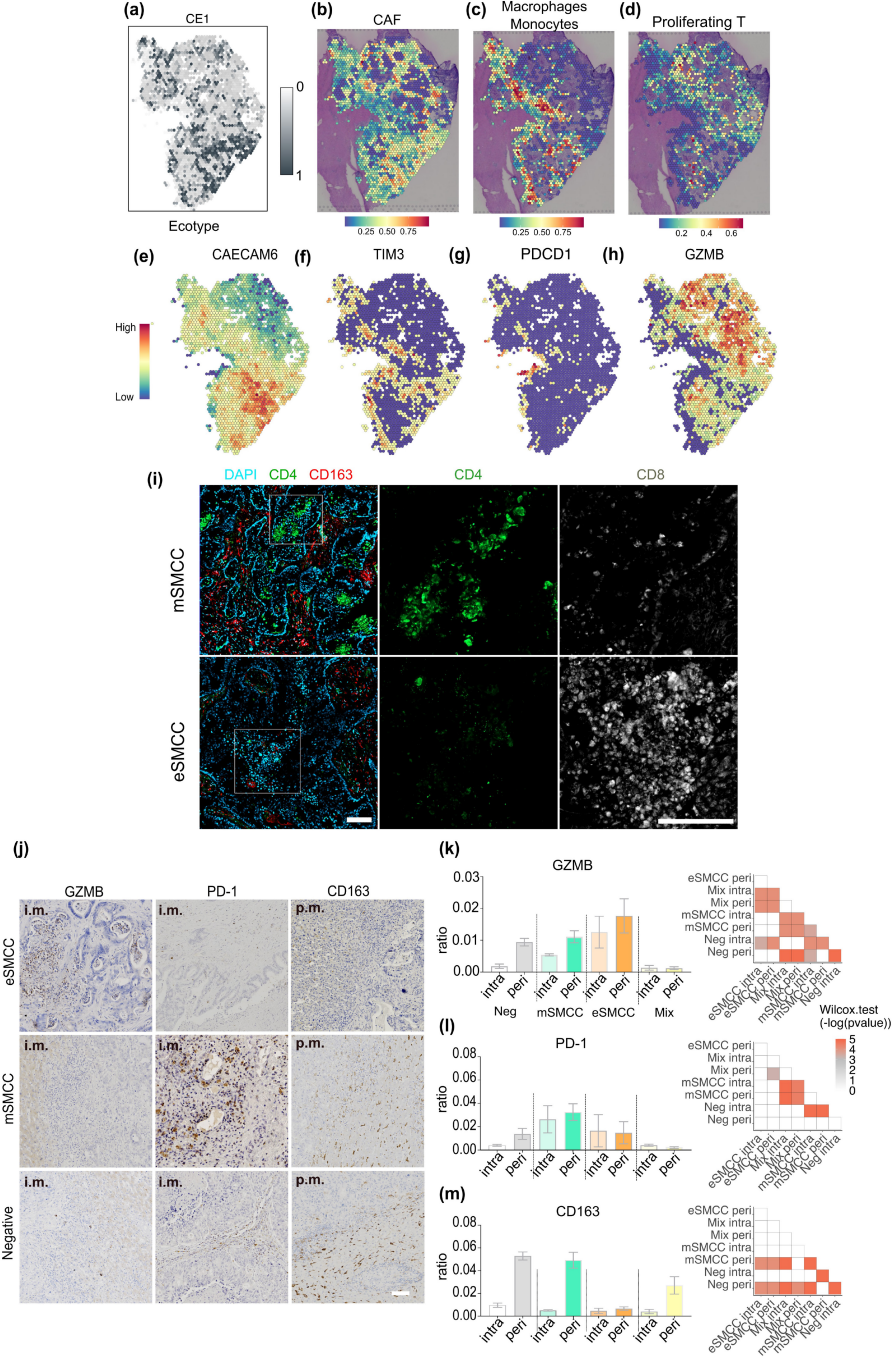
Immuno‐modulation properties of epithelial versus mesenchymal senescent metastatic cancer cells (SMCCs) in colorectal liver metastasis (CRLM). (a) CE1 ecosystem produced by ecotype analysis. (b) Integrated analysis of spatial data and single‐cell extracellular matrix (ECM) signatures from an independent CRLM study, showing CAF localization over meta1. Integrated analysis of spatial data and immune single‐cell signatures from an independent CRLM study, showing (c) macrophages and (d) proliferating T‐cell localization over meta1 specimen. Immunosuppressive (e–g) and (h) cytotoxicity markers activity maps localized in mSMCCs and eSMCCs ecosystems, respectively. (i) Z‐stack projection of CD4, CD163, and DAPI co‐staining on meta1 cryosection (left). CD4 and CD8 immunofluorescence corresponding magnifications (right). CD8 antigen immunostaining was performed on meta1 serial cryosection, bars 100 μm. (j) Immunohistochemistry for GZMB, PD‐1, and CD163 over our CRLM cohort (*n* = 5 for each group), showing that meta1 findings are reproducible. GZMB, shown in the intrametastatic area (i.m.), accumulates in eSMCCs comparing other groups. PD‐1 is shown in the i.m., while CD163 is shown in the perimetastatic area (p.m.). Digital quantification of GZMB (k), PD‐1 (l), and CD163 (m) markers randomized in intrametastatic and perimetastatic areas in epithelial and mesenchymal SMCCs, Negative and Mix patients. On the right, heatmap of Wilcoxon test applied to the data reported on the left (k–m). The values <−log(0.05) were considered not significant and reported in white.

We then determined the cellular composition of the local micro‐environment. EcoTyper—a machine‐learning framework for large‐scale identification of cell states and multicellular communities (Luca et al., 2021)—assigned specific carcinoma ecosystems (CEs) within meta1. CEs have predictive cellular, genomic, and clinical outcomes that are strongly conserved across 16 types of human carcinomas. This approach extrapolated three clinically distinct CEs localized in specific metastatic areas: CE1 was characterized by stimulation of CAF transcription, a high risk of death, M2‐like macrophages, and lymphocyte deficiency, and was mainly identified in mesenchymal ECM (Figure 6a); in contrast, CE9 and C10 were characterized by strong immunoreactivity, associated with longer overall survival, were typical of highly infiltrated tumors, and represented at the perimetastatic boundaries (Figure S6a,b). To confirm the CE1 phenotype and to overcome the technical gap in the single‐cell resolution of ST, we clustered our spatial data with two single‐cell databases (Figure S6c–f, workflow). The integration between ST data and single‐cell atlas of primary CRCs and liver metastasis (Che et al., 2021) revealed an overlap between the CAF signature and mesenchymal ECM, a phenotype absent in the epithelial area (Figure 6b). We then integrated our spatial stromal transcriptome with single‐cell RNA‐seq data of treated CRLM‐derived immune subpopulations (Che et al., 2021). As expected, M2‐phenotype macrophages were present in correspondence with the CE1 ecosystem (Figure 6c and Figure S6g,h). Colony‐stimulating factor 1 (*CSF1*) and C‐X‐C motif chemokine ligand 1 (*CXCL1*), known to attract macrophages and neutrophils, were significantly upregulated in mesenchymal crypts (Figure S6i,j), as well as several immunosuppression‐related genes (Cagnoni et al., 2021; Chiavarina et al., 2021; Liu et al., 2018; Patry et al., 2015) (Figure 5e–g). CAFs and M2 macrophages are known to produce TGFβ (Tauriello et al., 2022). Therefore, we reasoned that since in our setting those populations were spatially identified in mSMCC surrounding and physically overlapped (Figure S6l,m), they would be the primary source of TGFβ and mSMCC induction. In contrast, the eSMCC ecosystem, deficient in TGFβ1, had active immune flow in which T and NK cells as well as their cytotoxic product Granzyme B accumulated massively (Figure 6d,h and Figure S6k). To validate our ST data, we performed a co‐staining between M2 and CD4/CD8 markers on meta1 (Figure 6i). As predicted, CD163‐positive macrophages (red) accumulated around the mSMCC crypts, that were enriched in CD4‐positive cells (green), whereby eSMCCs showed higher CD8 infiltration and less CD163 accumulation in the ECM. Finally, we extended this analysis in our CRLM cohort (Figure 6j and Figure S4b, *n* = 5 for group). As expected, the four groups acted distinctly for GZMB, PD‐1 and CD163 markers in terms of expression and distribution (Figure 6k–m). In the eSMCCs group, GZMB colonized the entire metastasis, while for PD‐1, most of the patients showed lower intrametastatic expression compared to mSMCCs. Only one eSMCC biopsy did not reflect this trend. M2 cells were scarce in both intra‐ and perimetastatic areas. In the mSMCCs group, GZMB and CD163 accumulated significantly in the perimetastatic zone, while PD1 was abundantly found in and out of the metastasis, suggesting the establishment of an immunosuppressive environment. Notably, in negative patients, we observed a conserved accumulation of immune cells in the perimetastatic area compared to the intrametastatic zone for all the immunogenic markers. The fact that the Neg group mimicked GZMB and CD163 mSMCC distribution, but not PD‐1, could partially explain the discrepancy in the impact on relapse showed in Figure 5g. Finally, Mix's patients resembled eSMCCs group trend for cytotoxic and immunosuppressive markers, but at lower levels, and for M2 in the intrametastatic area, while recruiting more M2 in the perimetastatic zone (Figure 6k–m). The three patients with spatially isolated SMCCs signatures recapitulated the corresponding immunogenic findings (Figure S4b).

## 3 DISCUSSION

This study exploits spatial transcriptomics to decipher the biological significance of senescent cancer cells throughout CRC evolution, finding that SMCCs are leading players in metastasis and tumor outgrowth. The novelty of our work resides in the discovery that the clinical outcomes of CRC and CRLM depend on these non‐proliferating cancer cells. Neo‐adjuvant chemotherapy, the gold‐standard therapy for CRLM, consists of the administration of anti‐proliferative drugs to reduce tumor size and allow easier metastasis resection. Our findings suggest that this first hit could compromise patient fitness, encouraging the establishment of an mSMCC phenotype, which would determine a fatal outcome. The choice of specific chemotherapeutic agents has to be carefully weighted from clinicians before any clinical intervention, due to their impact on mSMMCs incidence.

It would be important to understand whether eSMCC and mSMCC populations evolve reciprocally, as the EMT trajectory suggests. The partial overlap between SMCCs and Doxo‐induced signatures might suggest that those two processes can be concomitantly active. However, it is difficult to establish whether the distinct mechanisms of senescence induction, cell autonomous versus paracrine, are biologically coordinated and what could determine the complete evolution from a Mix to a “pure” signature. The absence of a clinical predictive role for iSMCCs or not‐SMCCs would further support the complexity of senescence behavior in cancer. Indeed, cells with EMT hybrid signatures have shown more effectiveness in circulation, colonization and development of metastasis (Pastushenko et al., 2018). However, we are aware that our evaluation is limited to CRC samples.

Research focuses on genetically aggressive variants that confer a transcriptional survival advantage (Birkbak & McGranahan, 2020; Giessler et al., 2017; Jones et al., 2008). We found no differences in the transcriptional signature of senescent cancer cell subtypes in primary versus secondary organs, meaning that some cancer cells are “born to be bad.” The clinical outcome seems to be determined by the percentage of SMCC subtype within cancer. However, which factors determine the multicellularity of metastasis and the kinetics of progression need investigation. Whether the predictive power of SMCC signatures depends on origin (epithelial or mesenchymal) or is more strictly related to senescence is another open question. Our findings suggest that SASP is linked to stressor type rather than cell origin. Indeed, eSMCC SASP overlaps with RAS‐induced senescence in fibroblasts rather than with IRR epithelial cells. Evidence for a connection between senescence and EMT comes from the observation that transcriptional signaling driving senescence is also involved in EMT (Smit & Peeper, 2010). Therefore, sharing a commitment to cancer progression is indisputably a functional overlap (Muñoz‐Espín & Serrano, 2014).

Therapeutically, the presence of eSMCCs is a desirable final stage, recalling the historical role of senescence as being tumor‐protective (Bennecke et al., 2010; Sieben et al., 2018). Regarding the detrimental outcome of mSMCCs, there are at least three hypotheses: (i) mSMCCs are linked to an intermediate stage of EMT, and, therefore, to a reversible process, under which cells undergo transcriptional‐EMT chromatin reorganization. Effectively, in several human pathologies and experimental models, tumor cells are released from senescence with high aggressive growth phenotype (Milanovic et al., 2018; Roberson et al., 2005; Saleh et al., 2020). Additionally, cellular senescence has been described as the steady state of somatic cell reprogramming, that if overcome would allow an efficient transition to a stem‐cell phenotype (Li et al., 2021). Those findings underlie the unexpected plasticity of senescent status, which represents a bottleneck for multiple cell‐type transitions. (ii) mSMCCs could become “revertant” cells, that is, cancer cells with low proliferating ability but highly increased migration and invasion (Yang et al., 2017); and (iii) mSMCCs could be irreversibly arrested in G1, but could forage the non‐senescent cancer cells to survive. In any case, senolytics tailored to the mSMCC molecular signature represent a novel area of investigation for the development of effective CRLM therapies.

One of the limits of our ST approach is the lack of single‐cell resolution, which restricts the findings on small cells and in the case of overlapping signatures masks the weakest or the less representative ones. By integrating our spatial transcriptome with single‐cell RNAseq CRLM‐derived immune subpopulation, we observed different immune landscapes around SMCCs areas, confirmed by immunohistochemistry. It is tempting to speculate that, as for primary CRC, CSF1 secreted by mSMCCs could drive M2 macrophage polarization, resulting in CD8^+^ T cell inhibition (Choi et al., 2021). Further studies are required to dissect the contribution of SMCCs to immune response. Recently, immunogenic properties of senescent cells have been validated in experimental murine models and human cancer cell lines (Chen et al., 2022; Marin et al., 2023). Whether those properties are relevant to CRLM pathology needs to be properly investigated. Additionally, understanding how targeting senescent cancer cells impacts tumoral immune micro‐environment or how the immune system can be harmed to drive the clearance of bad SMCCs will be crucial to enroll senotherapies for clinical trials (Amor et al., 2020; Waldman et al., 2020).

We have deciphered here the significance of the spatial organization and transcriptional program of senescent cancer cells in colorectal liver metastasis. We have elucidated the micro‐environmental landscape, including secretory properties and TME–cell interactions. We have demonstrated that SMCCs play an important role in shaping the physical outgrowth of metastasis. The coexistence of two SMCC populations with opposing effects can explain heterogeneity in relapse and drug resistance. Individually, SMCC signatures have a consistent prognostic role in all the steps of CRC evolution, highlighting their significance for the development of new therapeutic approaches in colorectal liver metastasis.

## 4 METHODS

### 4.1 Human samples

From the existing database at the IRCCS Humanitas Research Hospital, we have retrieved 68 patients according to the following inclusion criteria: histologically proven diagnosis of CRLM; availability of the full data for analyses including follow‐up. Diabetics, obese, HCV‐positive, HBV‐positive, and alcohol abusers were excluded. All patients were further randomized into Group A (patients treated only with hepatectomy) and Group B (patients treated with neo‐adjuvant chemotherapy followed by hepatectomy). The biological specimens consist of CLM tissue and normal non‐tumoral adjacent liver parenchyma. Additional 32 subjects were also enrolled with histologically proven diagnoses of CLM. The CLM specimens, consisting of CLM and corresponding non‐tumoral hepatic parenchyma, were analyzed in part for β‐gal positivity, and in part for spatial transcriptomic technology. β‐gal stained sections were subjected to pathology review to confirm that the positivity for cellular senescence marker was histologically consistent with the tumoral part and does not belong to other histological compartments.

All patients were enrolled into the clinical protocol entitled “Studio dell'interazione tra la senescenza cellulare e sistema immunitario nelle metastasi epatiche umane da tumore primario al colon: alla scoperta di un nuovo possibile marker prognostico,” which was approved and registered by the local institutional ethical committee (Humanitas 564/21). Each patient signed a written informed consent for general research purposes. Demographics and clinical characteristics from the retrospective cohort and the freshly removed specimens with β‐gal scoring are in Table S1.

### 4.2 Methods details

#### 4.2.1 Collection and preparation of CRLM tissue for spatial transcriptomic

The CRLM specimens have to be processed within 30 min from the surgical removal and for β‐gal enzymatic assay. Those are the best practices for preserving RNA integrity and β‐gal enzymatic activity, which would become undetectable after only one night at −80°C. CRLM specimens were embedded in Optimal Cutting Temperature (OCT) compound and snap‐frozen in a bath of Isopentane (2‐Methylbutane) and liquid nitrogen. Four serial sections for each specimen were immediately cryosectioned at 10 μm thickness. Three sections were processed for RNA Integrity Number (RIN), one for β‐gal assay. Cardinal points were signed on the OCT blocks, to re‐place the tissue block on the cryostat stage with the initial orientation, for the sectioning of desired areas identified after β‐gal staining.

#### 4.2.2 RNA quality assessment

The RNA was isolated using Qiagen RNeasy Mini Kit (Cat#74104), following manufacturer's recommendations. RNA quality control was performed with the Agilent 4200 Tape Station system using the High Sensitivity RNA ScreenTape (5067‐5579) analysis kit (Agilent); only RNAs having a RIN ≥7 were used for Spatial transcriptomic technology.

#### 4.2.3 Tissue optimization protocol

This step was necessary for the optimization of permeabilization procedure, which is tissue‐dependent. We employed ad hoc Visium Spatial Tissue Optimization Slide which contains canonical Capture Area printed with capture sequences for mRNA capture but lacks spatial barcode array. The procedure involved testing times of 60, 45, 30, 20, 10, and 5 min on serial cryosection of a CRLM sample. The release step of the surface probes was not performed, and the reverse transcription mixture contained the same reagents except for 0.5 mM of each dATP/dGTP/dTTP, 12.5 μM dCTP, and 25 μM Cy3‐dCTP. Glass slides were scanned in AxioScan microscope. Dim and low fluorescence signals indicative of suboptimal permeabilization were excluded; we established that the best condition for CRLM was 15 min of enzymatic digestion.

### 4.3 Spatial transcriptomic procedure

We select 4 CRLM samples based on RIN >7. Two of them were also pre‐screened for β‐gal assay resulting in positive. We mounted the selected OCT tissue blocks on the specimen stage aligned as previously based on cardinal signs and cut serial cryosections which have been placed onto Tissue Preparation Guide (CG000240). For the following steps, we acted by Visium Spatial Gene Expression protocol (CG000239) with minor modifications.

#### 4.3.1 Staining and imaging

Visium Spatial Gene Expression slide, containing the four CRLM sections, was incubated following the Methanol Fixation + H&E Staining guide (CG000160). Finally, the slide was dried at 37°C for 5 min and mounted with Mounting Medium (22.5 μL RNase inhibitor, 7.5 μL Nuclease‐free water and 170 μL Glycerol). The slide was imaged with AxioScan, in brightfield with 20× magnification. After acquisition, the Spatial Gene Expression slides were immersed in a 3× SSC solution in ultrapure water for 20 min, to remove the coverslip.

#### 4.3.2 Permeabilization, reverse transcription, denaturation, and cDNA generation

Permeabilization enzyme was equilibrated at 37°C for 15 min before proceeding. The slide was assembled into the Visium slide cassette to perform the enzymatic reaction on a thermocycler adaptor. Seventy μL of Permeabilization enzyme was uniformly added to each well, and the Visium slide cassette was incubated on a thermal cycler at 37°C for 15 min. Following Permeabilization step, we performed Reverse Transcription, Second Strand Synthesis and Denaturation. cDNA generated was transferred in a pcr tube from the slide. One spatially barcoded full‐length cDNA was amplified for 12 cycles and the other three cDNA for 14 cycles determined on the *C*_q_ value by qPCR amplification plots following the manufacturer's protocol (CG000239). After, the cleanup by SPRIselect beads, we evaluated the cDNA concentration by Qubit dsDNA HS Assay Kit (Q32854); a quality profile was performed with the Agilent 4200 Tape Station system using the High Sensitivity D5000 ScreenTape (5067‐5592) analysis kit (Agilent).

#### 4.3.3 Library construction and sequencing

Twenty‐five nanogram of the amplified cDNA was then used for each sample to construct Illumina sequencing libraries following the manufacturer's protocol (CG000239) using 17 cycles and Dual Index Plate TT set A for their generation. Final libraries were checked by Qubit dsDNA HS Assay Kit (Q32854) and the Agilent 4200 Tape Station system using the High Sensitivity D5000 ScreenTape (5067‐5592) analysis kit (Agilent). Sequencing was performed on the NextSeq550 Illumina sequencing platform following the run parameters such as Reads1 = 28; i7Index = 10; i5Index = 10; Reads2 = 90 and calculating sequencing depth requires estimating that the approximate capture Area covered by tissue is about 85%.

#### 4.3.4 Immunohistochemistry

Immunohistochemical analysis of CRLM tissues was performed on FFPE 3‐μm‐thick serial sections. The FFPE sections were deparaffinized, pre‐treated with Target retrieval solution, ph9 (1×) at 98°C (20 min) and then incubated o/n at 4°C (or 1 h a RT for γH2A.X antibody) with primary antibody anti‐human ki67 (DAKO, #F7268, 1:200), anti‐human CK20 (DAKO, # M7019, 1:200), anti‐human p21^Cip1^ (DAKO, #M7202 1:200), anti‐human γH2A.X (Cell signaling, #9718, 1:500), anti‐human NOX4 (Abcam, # 133303, 1:200) and anti‐human p15^Ink4b^ (Orbit, orb30654, 1:50). The antibodies were diluted in Universal Antibody Dilution Buffer. For anti‐human p16^Ink4a^, serial sections were pre‐treated with DIVA retrieval solution at 98°C (20 min) and then incubated for 1 h a RT with primary antibody (ready to use provided). For Granenzyme B (R&D, AF1865, 1:200) and CD163 (Leica Biosystems, PA0090, 1:200) antibodies, tissue sections were pre‐treated with Citrate solution at 98°C (20 min) while for PD1 antibody (ab52587, 1:100) with DIVA Decloaker solution (Biocare Medical, SKU DV2004) and then incubated o/n at 4°C with primary antibody. Endogenous peroxidase was blocked for 20 min in Real Peroxidase blocking solution.

Envision + System HRP Labeled Polymer anti‐Mouse, anti‐Rabbit, or anti‐Goat HRP‐Polymer kits was used as a secondary antibody, depending on the species of the primary antibody. After washing, slides were developed with DAB (3,30‐diaminobenzidine) (Betazoid DAB Chromogen Kit; Biocare Medical #BDB2004) and counterstained with Hematoxylin. Tissues were dehydrated with ethanol, mounted with Eukitt, and analyzed with an Olympus BX61 virtual slide scanning system or Axio Scan. For p21^Cip1^ (Origene, TA808128, 1:150) staining on Doxo‐treated cells, we applied the same conditions described above for tissue, avoiding the antigen retrieval treatment.

#### 4.3.5 Images analysis

To obtain the immune reactive area for each marker, tissue slides were digitized after staining procedure using a computer‐aided slide scanner (Axio Scan). An expert pathologist selected the same areas which overlapped with the previous staining for p16^Ink4a^, p21^Cip1^, p15^Ink4b^, and γH2AX. Since most CRLM patients receive neo‐adjuvant systemic chemotherapy leading to tumor necrosis to variable extents, we avoid necrotic areas often found in the tumor core region. The pathologist defined the peritumoral region based on a manual definition of the tumor and extracellular matrix surrounding the crypts. An image analysis software (QuPAth) was used to automatically determine the percentage of immune reactive area of the digitized images. The mean value, obtained from the three different microscopic areas, was calculated for each patient and used for subsequent analyses. With these criteria, we establish the immunogenicity of our patients accordingly to the previous classification in eSMCCs, mSMCCs, Mix and negative. Five patients representative of each category were analyzed.

#### 4.3.6 Immunofluorescence

The protocol for serial cryosection and HCT‐116 cells differs only for the mix of primary antibodies applied. The material was fixed at 4°C for 5 min in 4% of paraformaldehyde. Then, after three washes in PBS1× (5 min each), it was permeabilized in ice‐cold acetone for 5 min. After three washes in PBS1× (5 min each), the section was incubated for 30 min with blocking solution at room temperature (2.5% Bovine serum albumin in PBS1×). The cryosection was incubated for 1 h with anti‐human RPL11 (Thermo Fisher, Cat# PA5101381, 1:200) and anti‐human CD8 (Agilent, M7103, 1:200), while Doxo‐treated HCT‐116 cells and controls were incubated with anti‐human RPL11 and anti‐human MDM2 (Santa Cruz Biotechnology, sc‐965, 1:100). The antibodies anti‐Phospho‐Histone H2A.X (Millipore, code 05‐636, 1:500) and anti‐Phospho ATR (GeneTex, GTX128145, 1:500) were incubated o/n at 4°C. The slice and the cells were then incubated with the appropriate fluorophore‐conjugated secondary antibody. Then, they were processed for the staining with 594 conjugated‐fibrillarin. The biological material was fixed again with 4% of paraformaldehyde for 5 min at room temperature, washed three times with PBS1× for 5 min, and block for 30 min with 2.5% BSA solution. Then, it was incubated o/n at 4°C with anti‐human fibrillarin‐594 conjugated (Santa Cruz Biotechnology, sc‐166001 AF594, 1:50). The immunophenotyping on meta1 cryosection was performed with anti‐CD4 (ThermoFisher, PA5‐85858, 1:200) anti‐mouse anti‐human CD163 (BD Bioscience, clone GHI/61, cat. 562669, 1:100) antibodies. Before imaging, nuclei were counterstained with (DAPI). Cell line experiments were imaged with Axio scan, z‐stack projection (0.5 μm layers) with magnification 40×. Whole specimen imaging was performed with both Axio scan and Leica TCS SP8 Stimulated Emission Depletion (STED) super‐resolution microscopy and analyzed with corresponding software.

#### 4.3.7 β‐Galactosidase enzymatic assay

Fresh surgically removed human specimens were OCT embedded and flash‐frozen in liquid nitrogen. Immediately after, 10‐μm‐thick serial sections were cut, placed onto poly‐adenylated slides, and processed for β‐galactosidase staining with DBA kit (Cat#AB102534). The frozen slices were kept at room temperature for 5 min and then washed twice with PBS1× for 1 min. Then, they were fixed with a fixative solution for 10 min at room temperature, washed twice with PBS1× for 1 min, and placed in incubator at 37°C o/n with β‐galactosidase staining solution provided by the kit. The day after the slides were washed three times with PBS1× and counterstained with nuclear fast red. Then, they were dehydrated with ethanol, mounted with Eukitt, and analyzed with an Olympus BX61 virtual slide scanning. The same steps were performed also for HCT‐116 treated cells and controls.

#### 4.3.8 Induction of cellular senescence in HCT‐116 cell line and RNAseq analysis

About 2 × 10^5^ cells were seeded into 6‐well plates. After 1 day of starvation, cells were exposed to 800 nM doxorubicin (Doxo) (Merck; Cat # D1515) for 48 h. On Day 2, Doxo was replaced with fresh media and cells were followed until Day 13 with regular media changes every 4 days. Control cells were treated in parallel with DMSO 800 nM.

For RNAseq, three independent experiments were performed. RNA was extracted with RNeasy micro kit (cat. No. 74004, Qiagen) and sequenced after control quality check. The reads of the poly(A) RNAseq analysis were mapped against reference genome GRCh38 with STAR Aligner v2.7.10a (Dobin et al., 2013), and count table was generated using the function featureCounts from Rsubread package v2.12.0 (Liao et al., 2019), using exon for the reads summarization. Genes with less than 10 raw counts in 1% of the samples or with hypervariable expression found with DaMiRseq package v2.10.0 were removed (Chiesa et al., 2018). Differential gene expression analysis was performed by DESeq2 package v1.38.3. DEG genes with pvalue_adj <0.05 and log2FC >1 or log2FC ≤−1 as were included (Love et al., 2014). Pseudo‐bulk conversion of SMCCs spatial transcriptional data were performed by AggregateExpression function from Seurat package.

#### 4.3.9 RT‐qPCR assay

Cells were homogenized by cryogenic step. Total RNA was purified using Direct‐zol RNA MiniPrep w/Zymo‐Spin IIC Columns. cDNA synthesis was performed using Superscript Vilo cDNA Synthesis kit (Life Technologies) following manufacturer's instruction and random primers (0.5 μg/μL, Invitrogen). Real‐time PCR reactions were carried out using the Fast Sybr Green PCR kit (QuantiStudio 7 Flex RrealTime PCR; Applied Biosystems). The relative expression levels were calculated by the $∆∆C_{T}$ method after normalization to the average of GADPH level. Primer sequences are listed above.

#### 4.3.10 Visium spatial gene expression data analysis

Fastq files were generated with 10× Genomics software Space Ranger v1.2.2 with spaceranger mkfastq function, quality checked with fastqc v0.11.8. The reads were aligned to the human reference transcriptome GRCh38 with spaceranger count command. The obtained count matrices were processed using R v4.0.5 and Seurat package v4.0.1. We applied the following exclusion criteria: meta1: 200 < UMIs > 12,000; 100 < features > 3500; meta2: 200 < UMIs < 8000; 100 < features < 3000; meta4: 200 < UMIs < 20,000; 100 < features < 4500; meta3:200 < UMIs < 10,000; 100 < features < 3500. The mitochondrial gene rate cutoff was >15%. Data were normalized using SCTransform function. Each dataset was first analyzed separately. The transcriptional clusters which map within liver parenchyma were removed; from the resulting four spatial transcriptomic datasets, we generated a unique integrated transcriptional map by Seurat SCTransform integration workflow, using 3000 integration features. Principal component analysis was performed with default parameters; data clustering was performed using FindNeighbors, combining the first 30 PCA dimensions with the FindClusters function Seurat R package implemented (resolution parameter = 0.4). The UMAP dimensionality reduction were generated with min.dist = 0 and n.neighbors = 9 options. The clusters were projected back into H&E images and Meta2 and Meta3 SA‐β‐gal stained sections to identify which transcriptional cluster overlaps with β‐gala‐positive areas. The individual analysis on meta1 specimen was performed accordingly to the procedure described above, with minor modifications (resolution parameter = 0.8). Cell cycle phase was extrapolated by CellCycleScoring function; spatial gene expression maps were generated by SpatialFeaturePlot function; imputed gene expression count matrix was generated by RunALRA function (SeuratWrappers package v0.3.0); EMT score was extrapolated from the EMTome database, using AddModuleScore function with default parameters. Trajectory's analysis was performed with PCA reduction, on the 3000 most highly variable genes of individual meta1 transcriptome, while the pseudotime EMT inference is generated by slingshot package v1.8.0., setting C_5 as starting cluster. eSMCC versus mSMCC Differentially Expressed Genes (DEGs) were obtained with the FindMarkers function (logfc.threshold = 0.3, min.pct = 0.2, MAST algorithm). In the volcano plot, genes were considered significant with *p*_val_adj <0.05 and avg_log2FC > 0.3 < −0.3; gene prevalence was calculated as the ratio of pct1 to pct2. GO pathway enrichment was performed on the significant DEGs, using enrichR package v3.0. GO_Biological_Process_2021 is the reference database. Regulons activity was inferred with Single‐Cell Regulatory Network Inference and Clustering (SCENIC v1.2.4). The ribosome biogenesis network was reconstructed by Cytoscape v3.9.0 with STRING database. Cell states and ecotypes recovery in Visium data were performed by EcoTyper tool setting “Carcinoma” for Discovery dataset and “Epithelial.cells” for Malignant cell origin. Deconvolution of immune cell type in the spatial transcriptomic dataset was performed by scvi‐tools v0.16.4 used in R with reticulate package v1.25. Finally, the eSMCC and mSMCC‐specific gene signatures were obtained, selecting the top 100 DEGs based on avg_log2FC with a *p*_val_adj <0.05. Bonferroni correction was applied based on the total genes present in the dataset. Unless mentioned otherwise, all plots were generated using R package ggplot2 v3.3.5.

### 4.4 Statistical analysis

Survival analysis and Kaplan–Meier curves on retrospective cohort were performed with survival v3.2‐11 and survminer v0.4.9 R packages. Hazard ratio (HR) was estimated with a univariate Cox regression. Kaplan–Meier curves of TCGA patients were obtained by the online tool GEPIA2 (http://gepia2.cancer‐pku.cn/); the patients were assigned to the high cohort when their signature expression was above the third quartile or to the low cohort when it was below the first one. The Wilcox test in ggpubr package v0.4.0 has been applied in all other analyses.

### 4.5 Study approval

All research was conducted by both the Declarations of Helsinki and Istanbul; all research was approved by the appropriate ethics institutional review committee (IRCSS Istituto Clinico Humanitas, Prot. Nr. CE HUmaniats ex D.M. 8/2/2013 564/21). Written informed consent was received before participation.

## AUTHOR CONTRIBUTIONS

O.G. and L.L. conducted the experiments and analyzed the data. L.L. performed bioinformatics analysis. G.B. performed spatial transcriptomics on human specimens. D.G. performed RNAseq experiments. C.P. provided technical support for Spatial Transcriptomics. V.M. contributed to data analysis. B.F., C.S., D.B., and F.P. contributed to immunohistochemistry experiments. G.C. contributed to human CRLM patients' statistical analysis. D.A., A. S., and T.L. contributed to histological diagnosis of CRLM patients. G.T. and M.D. contributed to human CRLM patient selection. F.F., O.G., L.L., G.C., V.M., and M.D. provided in scientific discussion. F.F. conceived the study interpreted the data and wrote the manuscript. O.G and L.L. are co–first authors since they had complementary skills, essential to achieve the final results.

## CONFLICT OF INTEREST STATEMENT

The authors have declared that no conflict of interest exists.

## Supporting information


Figure S1

Figure S2

Figure S3

Figure S4

Figure S5

Figure S6

Table S1

Table S2

Table S3

Table S4

Table S5

## Data Availability

The raw data are available on Gene Expression Omnibus (GEO) under accession number “GSE206552”.
