# Supplementary material for: Spatial resolution of cellular senescence dynamics in human colorectal liver metastasis
Source: Aging Cell. 2023 May 8;22(7):e13853. doi: 10.1111/acel.13853 (PMC10352575; doi:10.1111/acel.13853)
Supplement: Supplementary file 1 — Figure S1 [file ACEL-22-e13853-s006.pdf]

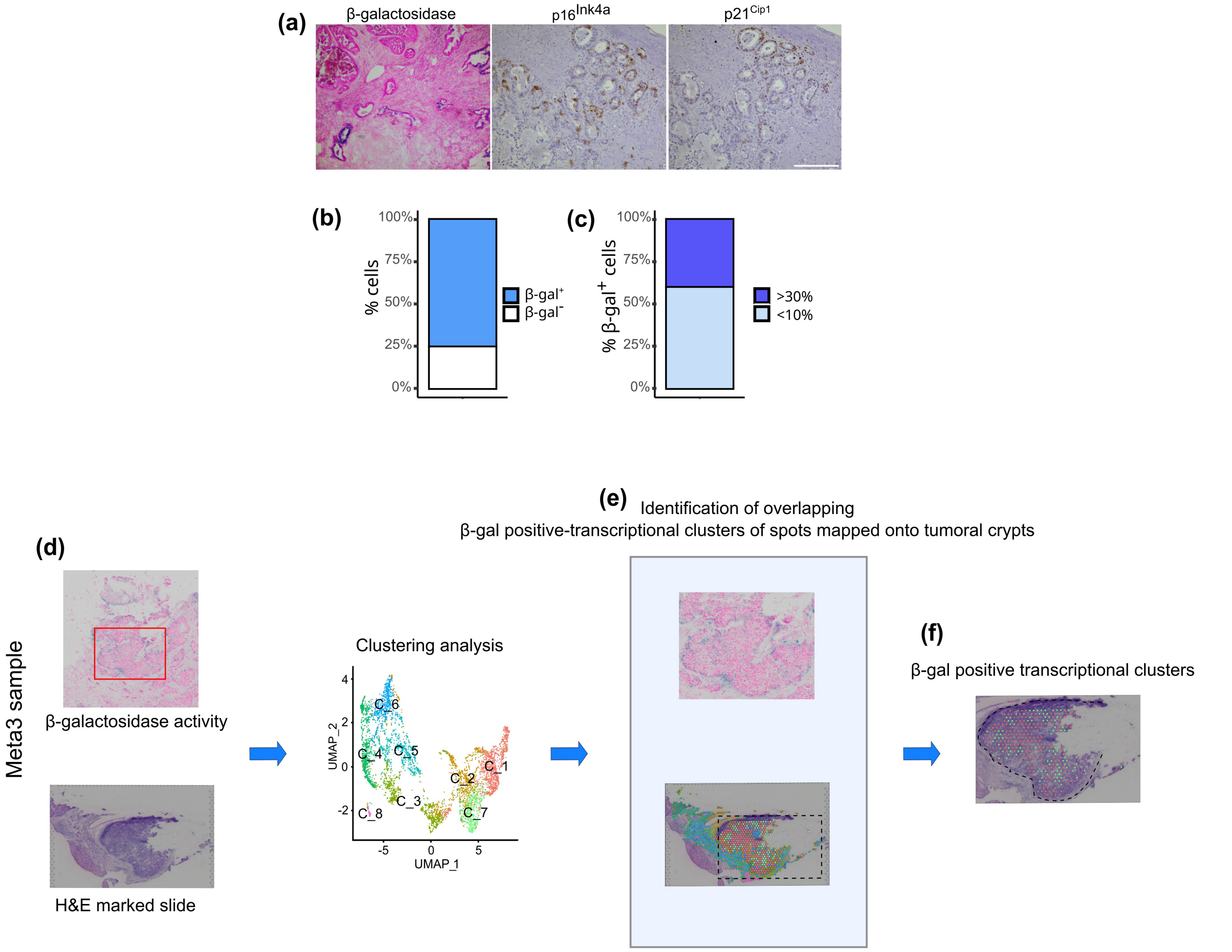

Supplementary Figure S1. Identification of senescent cells within CRLM specimens.

(a) Fresh CRLM specimens processed for either SA-β-gal staining and $p21^{Cip1}$ and $p16^{Ink4a}$ immunostaining on the corresponding FFPE sections. (b) Quantification of SA-β-gal positivity and (c) grading. (d) SA-β-gal staining. Spatial projection of SMCCs clusters into β-gal positive serial section of meta3 specimen. (e) Clusters projection onto meta3 H&E image. (f) SA-β-gal overlapping transcriptional clusters in meta3 specimen.
