# Supplementary material for: Spatial resolution of cellular senescence dynamics in human colorectal liver metastasis
Source: Aging Cell. 2023 May 8;22(7):e13853. doi: 10.1111/acel.13853 (PMC10352575; doi:10.1111/acel.13853)
Supplement: Supplementary file 2 — Figure S2 [file ACEL-22-e13853-s005.pdf]

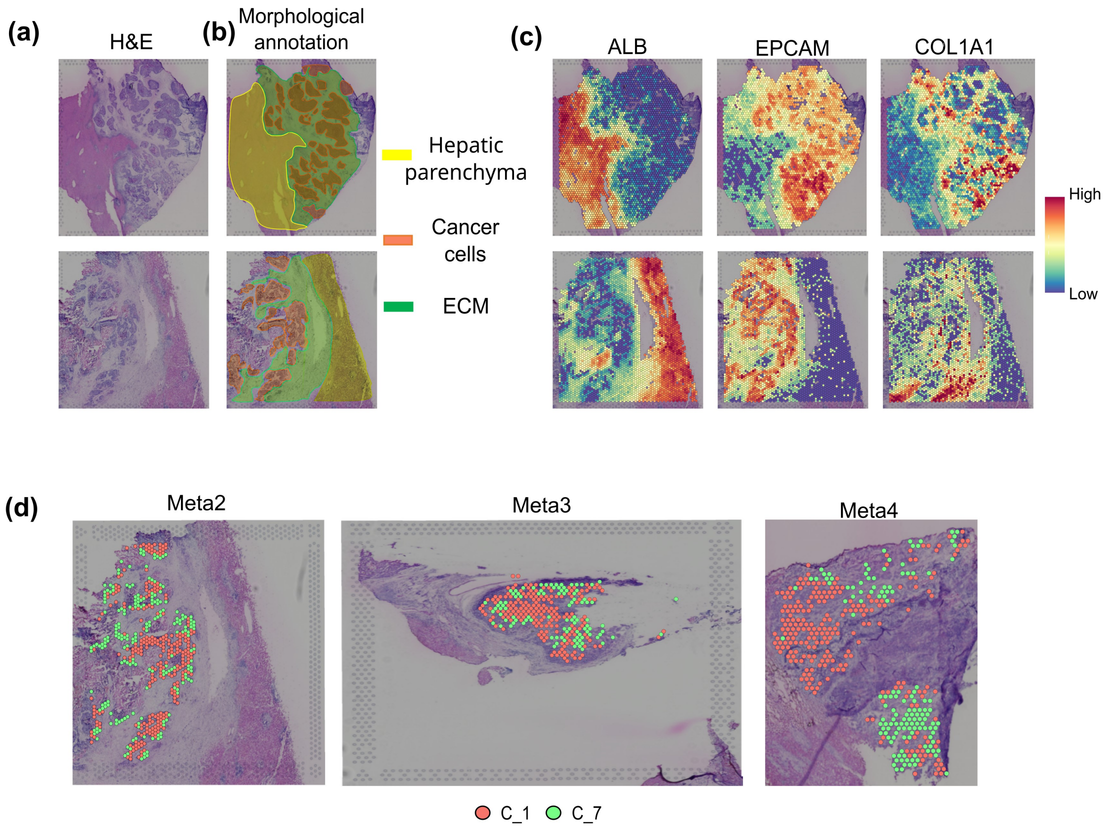

Supplementary Figure S2. Spatial data strongly recapitulate CRLM histology but SMCCs showed caotic distribution. (a) H&E images of meta1 and meta2. (b) Morphological annotation. (c) Activity maps of genes overexpressed in parenchyma (Albumin), tumoral crypts (Epcam) and stroma (COL1A1) are shown. Comparison between the panels overlapping confirmed overlap between annotated histological regions and the upregulated expected representative genes. (d) Spatial projection of C1 and C7 clusters into H&E sections of meta2, meta3 and meta4 specimens.
