# Supplementary material for: Spatial resolution of cellular senescence dynamics in human colorectal liver metastasis
Source: Aging Cell. 2023 May 8;22(7):e13853. doi: 10.1111/acel.13853 (PMC10352575; doi:10.1111/acel.13853)
Supplement: Supplementary file 3 — Figure S3 [file ACEL-22-e13853-s002.zip › ACEL_13853_S3_legend.pdf]

Supplementary Figure S3. SASPs differ in epithelial and mesenchymal SMCCs.

(a) Activity maps of genes involved in the molecular mechanisms of SASP induction, and (b) in the inflammatory arm of SASP. In eSMCCs there is evidence linking the DNA damage to NFKB1-dependent SASP expression. Indeed, NSB1, ATM and CHEK2, required for SASP initiation and maintenance, were upregulated (Figure 3l-n). TGFβ and inflammasome Retinoic Acid Inducible Gene I (RIG-1) dependent signalings are more likely mechanisms occurring in mSMCCs, while stress response dependent-overexpression of p38MAPK-NFKB1 axis seems a ubiquitous mechanism (a). Notably, NFKB1 do not regulate the entire SASP secretome, but usually acts synergistically with the transcription factor C/EBPβ, which regulate the inflammatory arm of SASP secretion[1]. In our setting, C/EBPβ and NOTCH1, its natural repressor, were respectively down and upregulated in eSMCCs; in mSMCCs they showed an opposite trend (b). (c) PCA plot showing the transcriptional profile of the poly(a)-RNAseq samples. The largest dots indicate the centroids of the samples in each group and the ellipsis represent the 95% of CI (Confidential Interval); (d) Heatmap showing 3234 DEGs genes between the DOXO-treated and the proliferating cells. (e) Venn diagrams showing overlapping between up-regulated genes in SMCCs and DOXO-treated HCT cells. Each group was first compared with HCT-116 proliferating cells. SMCCs ST data were converted in pseudo-bulk to allow the analysis with HCT-116 bulk RNAseq data. (f) SASP heatmap of DOXO-treated HCT-116 up-regulated genes after integration into SASP Atlas. The color bar represents LogRatio values. (g) Immunofluorescence for phospho-ATR and γH2AX on eSMCCs tumoral crypts, bar 50 and 20 μm.

1. Lopes-Paciencia, S. et al. The senescence-associated secretory phenotype and its regulation. Cytokine 117, 15–22 (2019).
