# Supplementary figures and images for: Spatial resolution of cellular senescence dynamics in human colorectal liver metastasis
Source: Aging Cell. 2023 May 8;22(7):e13853. doi: 10.1111/acel.13853 (PMC10352575; doi:10.1111/acel.13853)

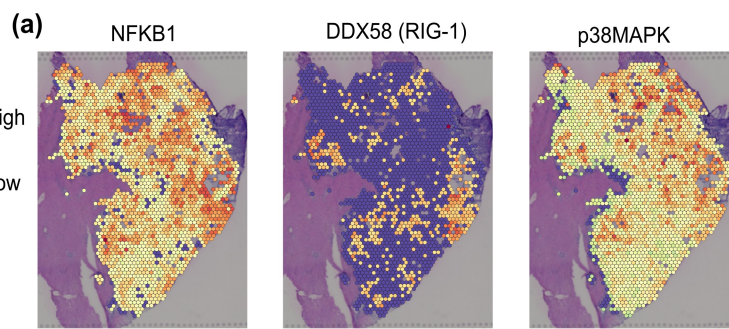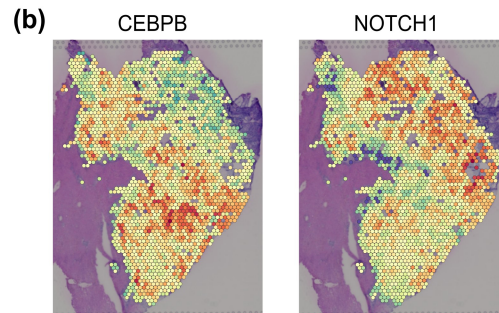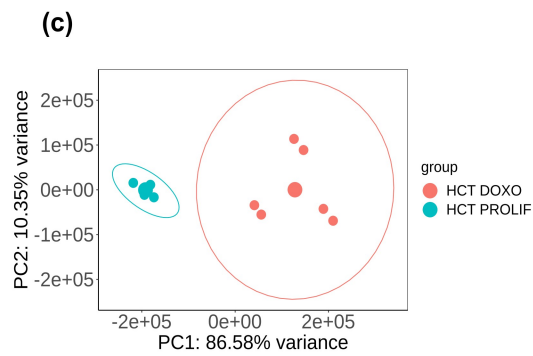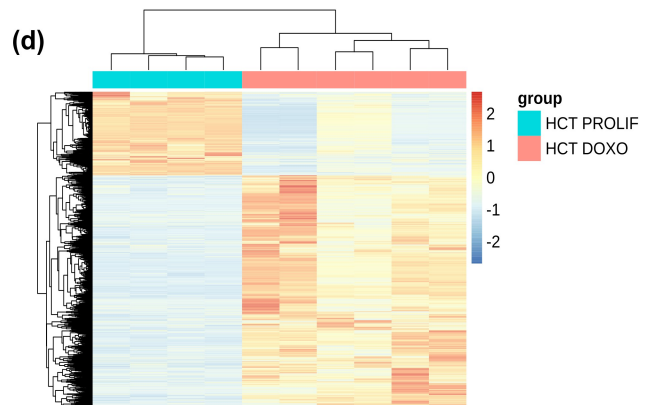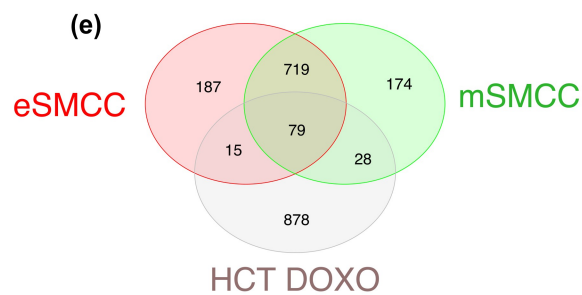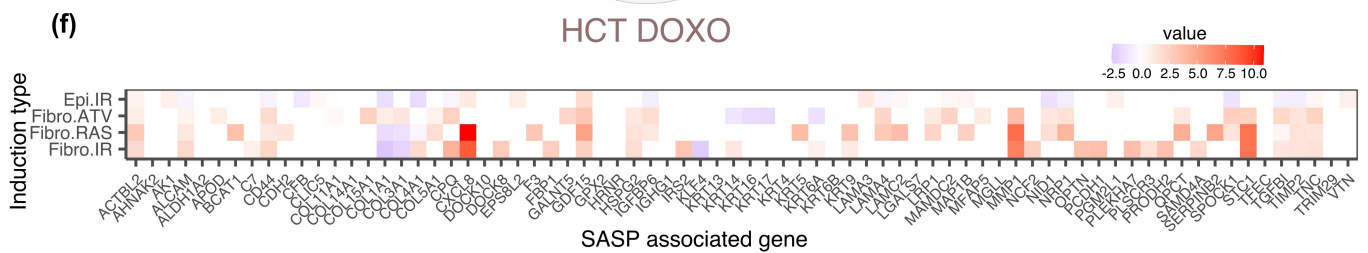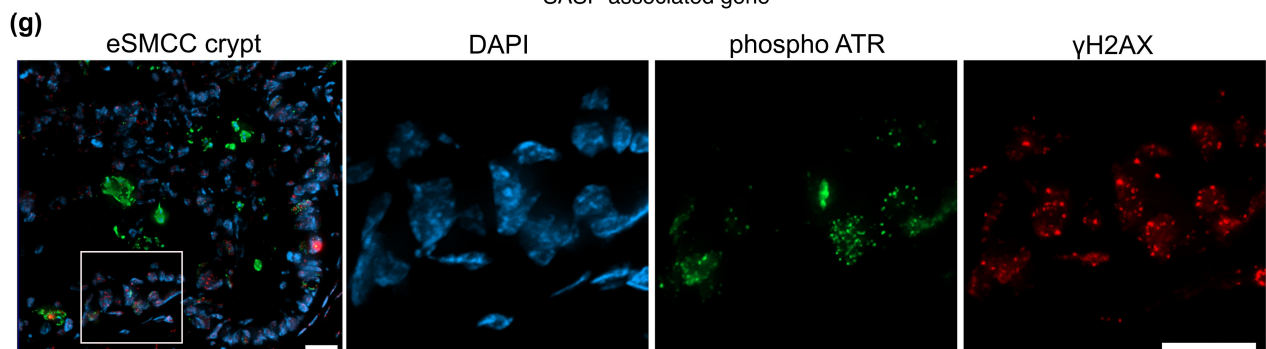

Supplement: Supplementary file 3 — Figure S3 [file ACEL-22-e13853-s002.zip › ACEL_13853_S3.pdf]

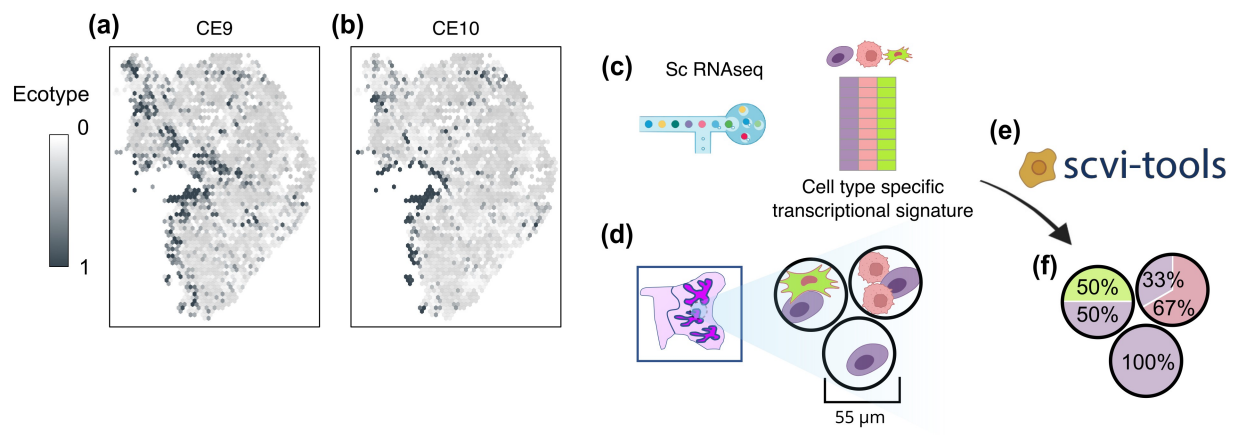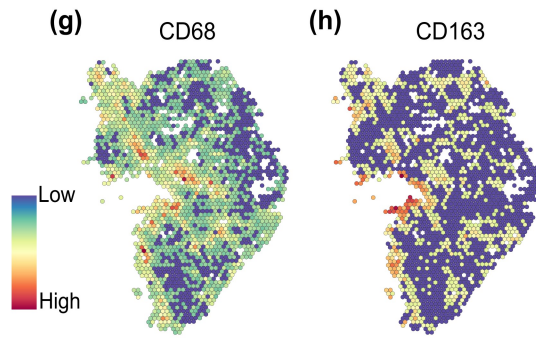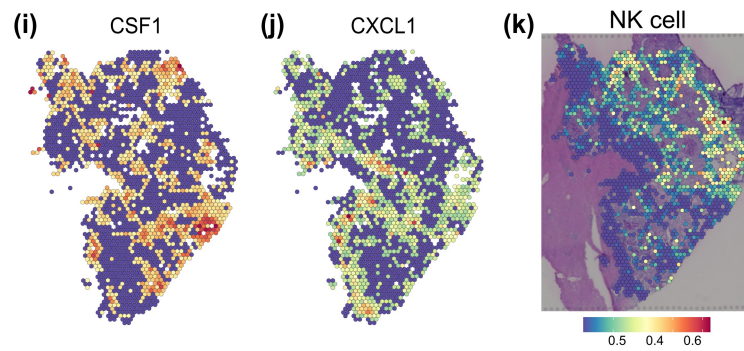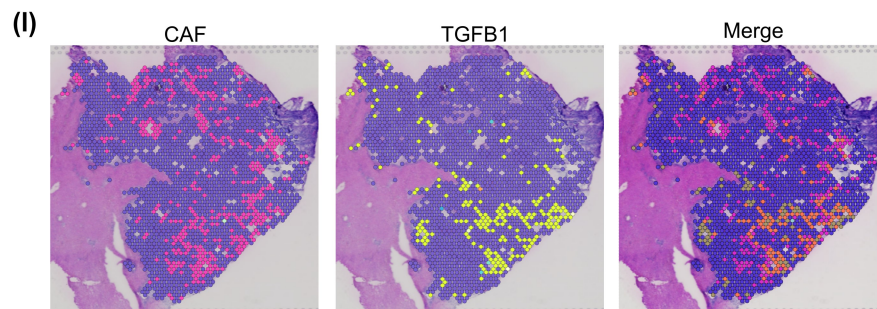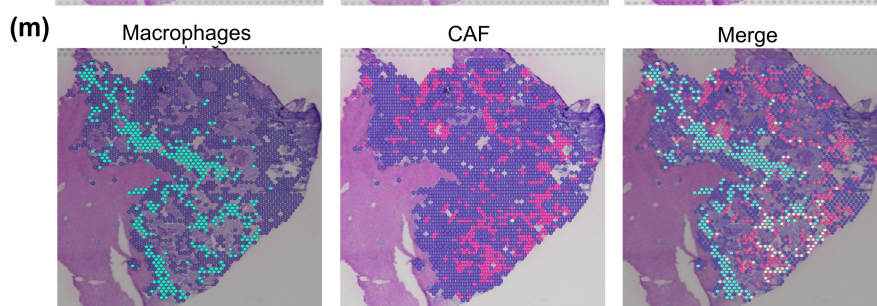

Supplement: Supplementary file 6 — Figure S6 [file ACEL-22-e13853-s010.zip › ACEL_13853_S6.pdf]
