# Supplementary material for: Spatial resolution of cellular senescence dynamics in human colorectal liver metastasis
Source: Aging Cell. 2023 May 8;22(7):e13853. doi: 10.1111/acel.13853 (PMC10352575; doi:10.1111/acel.13853)
Supplement: Supplementary file 4 — Figure S4 [file ACEL-22-e13853-s008.pdf]

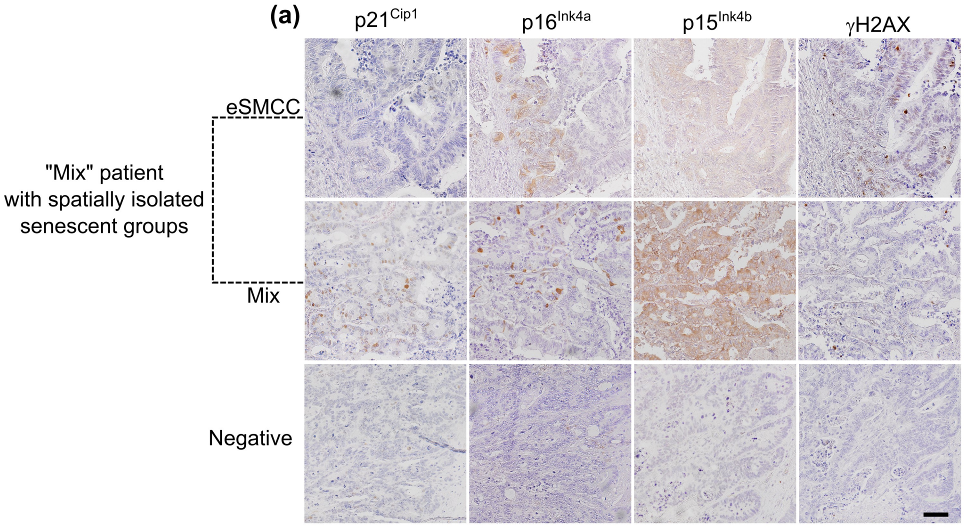

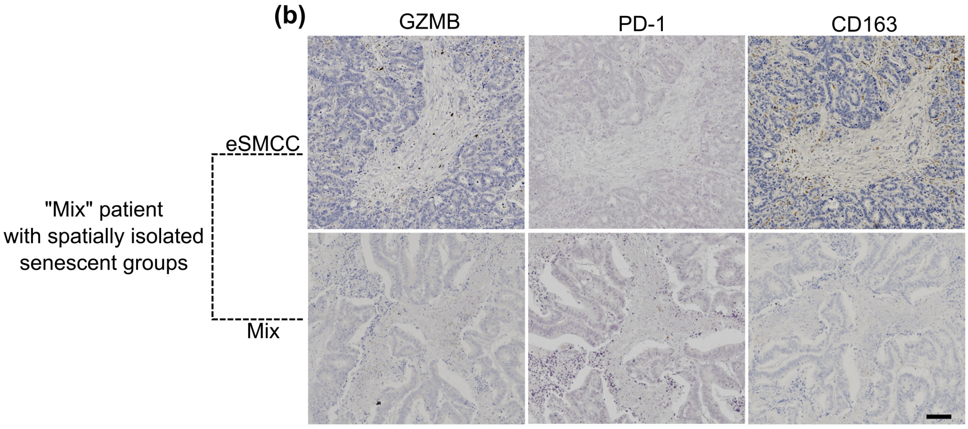

Supplementary Figure S4.

(a) 4-multilabeling immunohistochemistry for $p21^{Cip1}$, $p16^{Ink4a}$, $p^{15Ink4b}$ and γH2A.X on Mix and Negative CRLM patients, bar 200 µm. (b) Example of intrametastatic immunohistochemistry for GZMB, PD-1 and CD163 over Mix grouped patient in which SMCCs signature are spatially separated. Intrametastatic areas are shown, bar 100 µm.
