# Supplementary material for: Spatial resolution of cellular senescence dynamics in human colorectal liver metastasis
Source: Aging Cell. 2023 May 8;22(7):e13853. doi: 10.1111/acel.13853 (PMC10352575; doi:10.1111/acel.13853)
Supplement: Supplementary file 5 — Figure S5 [file ACEL-22-e13853-s001.pdf]

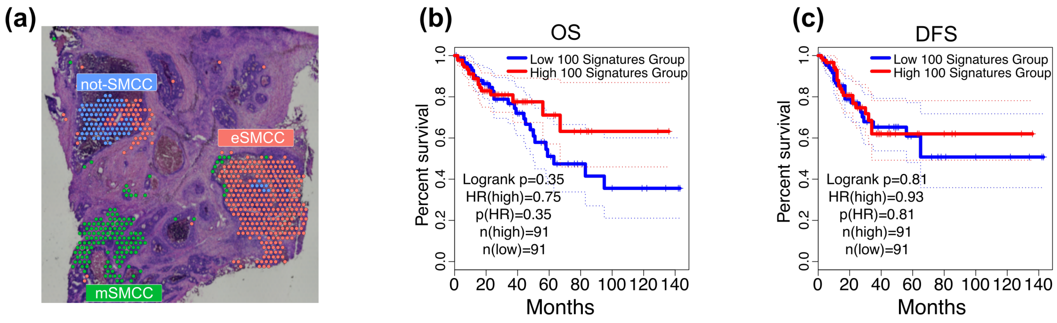

Supplementary Figure S5.

(a) ST projection of SMCCs signatures over CRC human sample. (b) OS and (c) DFS curve for not-SMMCs cluster integrated in TCGA database. OS= overall survival; DFS= disease-free survival.
