# Supplementary material for: Spatial resolution of cellular senescence dynamics in human colorectal liver metastasis
Source: Aging Cell. 2023 May 8;22(7):e13853. doi: 10.1111/acel.13853 (PMC10352575; doi:10.1111/acel.13853)
Supplement: Supplementary file 6 — Figure S6 [file ACEL-22-e13853-s010.zip › ACEL_13853_S6_legend.pdf]

Supplementary Figure S6.
(a-b) CE ecosystems produced by ecotype analysis. (c-f) Workflow of single cell integration data into our Spatial transcriptomic database. (c) Single cell RNA seq data from several public database (see Text for references) is a source of transcriptional signature specific for different known cell subtypes, that cannot be recognized per sè in the ST dataset (d) which lack single cell resolution. By integrating Sc RNAseq data into ST data thought the algorithm Variational Inference (DestVI) in the scvi-tools package (e), cell type proportions within every capture spot (55 mm) are recovered (f). (g) Activity maps of macrophages pan-marker (CD68), (h) M2 phenotype marker (CD163), CSF1 (i) and CXCL1 (j) SASP factors. (k) Activity maps of NK cells in meta1 specimen. (l) Overlay between CAF and TGFB1 and (m) CAF and macrophages are shown. To improve the overlay visualization, we arbitrarily set the gene expression threshold >1 for the cytokine and <=0.5 for Macrophages and CAF.
