# Supplementary material for: Spatial resolution of cellular senescence dynamics in human colorectal liver metastasis
Source: Aging Cell. 2023 May 8;22(7):e13853. doi: 10.1111/acel.13853 (PMC10352575; doi:10.1111/acel.13853)
Supplement: Supplementary file 7 — Table S1 [file ACEL-22-e13853-s009.pdf]

Table S1. Clinical data refer to the cohort of patients analyzed in this study.

Characteristic	**FFPE**	**FRESH**
Patients (number)	68	17
Age, Median range (years)	64 (29-83)	67 (47-80)
Sex, number (%)		
Male	48 (71)	10 (59)
Female	20 (29)	7 (41)
Primary tumor		
Adenocarcinoma - number (%)	68 (100)	17 (100)
Localization, number (%)		
Ascending colon, number (%)	19 (28)	9 (53)
Transverse colon, number (%)	3 (4)	1 (6)
Descending colon, number (%)	27 (40)	4 (24)
Rectum, number (%)	19 (28)	3 (18)
Synchronous, number (%)	16 (24)	13 (76)
TNM Colon Cancer Staging System		
Depth of infiltration (T), number (%)		
T1	4 (6)	0 (0)
T2	17 (25)	2 (12)
T3	46 (68)	12 (70)
T4	1 (1)	3 (18)
Regional lymph node involvement, number (%)		
N0	34 (50)	6 (35)
N1	25 (37)	8 (47)
N2	9 (13)	3 (18)
Metastases at the time of diagnosis (M), n (%)		
M1	16 (24)	13 (76)
Lesion, Median range (n)	3 (1-32)	3 (1-10)
Lesion size, Median range (cm)	4 (1-14)	3 (1-14)
CEA, Median range (ng/mL)	11 (1-2916)	15 (2-127)
Chemotherapy, n (%)	36 (53)	17 (100)
Cycles, Median range (n)	7 (2-19)	10 (3-39)
5-FU, n (%)	31 (46)	11 (65)
Oxaliplatino, n (%)	20 (29)	15 (88)
Irinotecan, n (%)	19 (28)	7 (41)
Bevacizumab, n (%)	9 (13)	10 (59)
Cetuximab, n (%)	10 (15)	3 (18)
