# Supplementary material for: Spatial resolution of cellular senescence dynamics in human colorectal liver metastasis
Source: Aging Cell. 2023 May 8;22(7):e13853. doi: 10.1111/acel.13853 (PMC10352575; doi:10.1111/acel.13853)
Supplement: Supplementary file 8 — Table S2 [file ACEL-22-e13853-s003.pdf]

Table S2. enriched regulons with Regulon Specificity Score (RSS).
e.= extended.

Regulon	RSS C_5	Regulon	RSS C_7	Regulon	RSS C_8	Regulon	RSS C_9
GTF2B_e. (32g)	0.405	CDX2_e. (42g)	0.273	FOSL2 (23g)	0.363	HOXB8_e. (29g)	0.218
DDIT3 (41g)	0.403	NPDC1_e. (48g)	0.273	SOX4_e. (42g)	0.324	JUND (87g)	0.217
NFYB (493g)	0.402	KLF5 (318g)	0.270	FOSL2_e. (296g)	0.301	SOX9_e. (33g)	0.214
DDIT3_e. (174g)	0.402	SOX4_e. (42g)	0.268	SOX4 (34g)	0.283	YBX1 (170g)	0.211
NFYB_e. (761g)	0.400	CDX2 (31g)	0.268	JUNB (36g)	0.274	JUND_e. (1625g)	0.209
MYC_e. (3678g)	0.396	CEBPA_e. (60g)	0.268	RUNX1_e. (38g)	0.273	HDAC1_e. (127g)	0.209
JUN_e. (319g)	0.396	KLF5_e. (1123g)	0.267	KLF5 (318g)	0.272	ATF5_e. (189g)	0.208
BRCA1_e. (783g)	0.395	HNF4A_e. (96g)	0.265	JUNB_e. (55g)	0.267	HSF1_e. (38g)	0.207
ATF1_e. (1334g)	0.393	HNF4A (65g)	0.263	ARID3A_e. (34g)	0.265	YBX1_e. (828g)	0.207
BRCA1 (611g)	0.393	TBX2_e. (56g)	0.262	CDX2_e. (42g)	0.261	E2F4 (741g)	0.206
