# Supplementary material for: Spatial resolution of cellular senescence dynamics in human colorectal liver metastasis
Source: Aging Cell. 2023 May 8;22(7):e13853. doi: 10.1111/acel.13853 (PMC10352575; doi:10.1111/acel.13853)
Supplement: Supplementary file 9 — Table S3 [file ACEL-22-e13853-s004.pdf]

Supplementary Table S3_DEGs eSMCCs Vs mSMCCs

gene	p_val	avg_log2FC	pct.1	pct.2	p_val_adj
H19	7,5E-50	1,61467117	0,968	0,347	1,2608E-45
NUPR1	1,4659E-47	1,53912999	0,968	0,629	2,4644E-43
RPS12	7,5889E-89	1,4497199	1	1	1,2758E-84
PRAC1	2,3014E-50	1,44718251	0,968	0,403	3,8689E-46
MT1G	2,9721E-42	1,3689636	0,936	0,379	4,9965E-38
RPS15A	1,4659E-83	1,35040952	1	1	2,4644E-79
SNHG5	9,9044E-66	1,34982578	0,992	0,879	1,665E-61
RPL22	6,173E-77	1,25603202	1	1	1,0378E-72
UQCRH	1,3576E-32	1,14338213	0,936	0,476	2,2823E-28
RPS27A	1,5331E-58	1,14089834	1	1	2,5773E-54
MT3	4,9043E-35	1,12935396	0,763	0,121	8,2446E-31
RPS13	3,5521E-54	1,12396886	1	0,96	5,9714E-50
SNHG25	3,4026E-33	1,10043443	0,944	0,476	5,7201E-29
NME2	1,9628E-47	1,02883138	1	0,944	3,2997E-43
SNHG8	4,3858E-30	1,00267586	0,924	0,5	7,3729E-26
RPS4X	1,1292E-64	0,98572684	1	0,992	1,8984E-60
RPL11	2,7669E-64	0,97930339	1	1	4,6514E-60
MICOS10	2,7321E-26	0,97803459	0,888	0,419	4,5929E-22
AREG	4,8595E-16	0,97331565	0,715	0,315	8,1693E-12
ATP5IF1	4,771E-23	0,92739101	0,876	0,5	8,0206E-19
GDF15	2,9329E-13	0,92269143	0,827	0,589	4,9305E-09
RPL26	2,755E-44	0,91146192	1	0,992	4,6314E-40
RPS3A	1,1121E-49	0,90530133	1	1	1,8695E-45
HMGN2	2,1513E-24	0,90300729	0,884	0,411	3,6166E-20
GAS5	2,9149E-30	0,88783257	0,996	0,847	4,9003E-26
RPS10	2,4593E-25	0,88201133	0,988	0,879	4,1344E-21
SNHG29	2,6114E-33	0,86895819	1	0,863	4,39E-29
RPS8	6,0637E-57	0,85611366	1	1	1,0194E-52
TOMM7	6,1744E-27	0,85101798	0,984	0,798	1,038E-22
NSMF	8,3539E-23	0,8426012	0,98	0,798	1,4044E-18
SNRPE	3,0637E-22	0,83912498	0,727	0,218	5,1504E-18
RPL34	9,4364E-65	0,83110198	1	1	1,5864E-60
ZFAS1	1,4062E-21	0,80043482	0,98	0,782	2,364E-17
H2AFZ	1,9562E-18	0,78802231	0,819	0,403	3,2885E-14
RPL35A	8,2123E-41	0,77859424	1	1	1,3806E-36
NDUFAF8	2,4117E-19	0,77587789	0,723	0,25	4,0542E-15
CHCHD2	7,2094E-22	0,77120469	0,972	0,661	1,212E-17
STMN1	1,9311E-18	0,76358145	0,663	0,202	3,2463E-14
ATP5PF	2,3874E-17	0,75244761	0,859	0,468	4,0135E-13
TOMM6	4,0819E-18	0,7507507	0,916	0,589	6,8621E-14
SLC25A6	7,2167E-20	0,74944045	0,928	0,718	1,2132E-15
UQCRB	1,6924E-27	0,73804207	1	0,903	2,8451E-23
RPL19	8,2592E-53	0,73451577	1	1	1,3885E-48
XIST	8,453E-22	0,7321901	0,59	0,089	1,421E-17
ATP5MPL	2,4174E-17	0,73217918	0,924	0,669	4,0638E-13
SNRPD1	1,1559E-18	0,72445643	0,655	0,185	1,9432E-14
RPL10A	4,4379E-34	0,7187825	1	0,968	7,4606E-30
RPL23A	1,1678E-22	0,71831963	0,992	0,895	1,9632E-18
RPS23	1,8888E-45	0,71564503	1	1	3,1753E-41
RPS24	4,8652E-33	0,70945667	1	0,984	8,1789E-29

NACA	9,6175E-29	0,70307233	1	0,984	1,6168E-24
RPS25	2,4955E-32	0,69186471	1	1	4,1953E-28
RPS26	9,5178E-17	0,69107068	0,968	0,79	1,6E-12
RPL27	6,8489E-30	0,6900712	1	1	1,1514E-25
IFITM3	6,66E-20	0,6871845	0,984	0,903	1,1196E-15
MME	2,1271E-17	0,68463155	0,727	0,266	3,5758E-13
ATP5PO	1,1334E-15	0,67096519	0,831	0,435	1,9054E-11
CCND2	2,5155E-13	0,66578396	0,855	0,556	4,2288E-09
MRPL13	7,9267E-16	0,66246963	0,659	0,25	1,3326E-11
TBCA	1,3478E-15	0,65539246	0,735	0,323	2,2657E-11
FAM3B	2,1992E-22	0,65425833	0,542	0,056	3,6971E-18
ATP5MC1	1,102E-14	0,65109077	0,667	0,25	1,8526E-10
LGR5	3,7241E-16	0,64189263	0,466	0,065	6,2606E-12
BTG2	1,003E-18	0,63672841	0,486	0,056	1,6861E-14
RPL41	5,6007E-31	0,63585917	1	1	9,4154E-27
FABP5	1,0418E-11	0,63259614	0,783	0,468	1,7515E-07
COA3	4,8643E-13	0,63023106	0,731	0,371	8,1774E-09
RPL12	3,2453E-39	0,62507059	1	1	5,4558E-35
HINT1	2,115E-12	0,62103223	0,867	0,597	3,5556E-08
AGR2	4,4129E-12	0,62037954	0,791	0,444	7,4185E-08
HSBP1	6,4471E-12	0,6103515	0,847	0,597	1,0838E-07
RPS28	1,0776E-34	0,60505874	1	1	1,8116E-30
TMA7	2,1024E-20	0,6004588	0,996	0,927	3,5344E-16
EPHB3	3,0037E-11	0,60002806	0,695	0,355	5,0496E-07
IFITM1	1,0924E-12	0,59893949	0,679	0,306	1,8364E-08
NDUFA4	6,1881E-12	0,59707879	0,851	0,524	1,0403E-07
RPS21	1,3104E-37	0,59454271	1	1	2,203E-33
SNRPD2	1,9068E-12	0,59365645	0,876	0,677	3,2056E-08
SELENBP1	2,0261E-11	0,58992331	0,855	0,524	3,4061E-07
WFDC2	3,0066E-16	0,5828232	0,446	0,056	5,0544E-12
TIMM13	1,8175E-11	0,58115093	0,843	0,54	3,0555E-07
ATP5MC2	3,0254E-12	0,57735464	0,896	0,637	5,086E-08
TSTD1	3,9055E-11	0,56420654	0,751	0,411	6,5656E-07
SELENOH	5,103E-11	0,56187889	0,755	0,427	8,5787E-07
RBIS	1,0068E-13	0,56162695	0,94	0,661	1,6925E-09
MRPL20	8,3339E-13	0,56100953	0,635	0,25	1,401E-08
RPS16	4,5562E-21	0,56079506	1	1	7,6594E-17
LSM5	1,0821E-12	0,55621095	0,683	0,29	1,8192E-08
NR4A1	1,7373E-07	0,55557006	0,598	0,331	0,00292063
RPS5	3,3667E-17	0,55280555	1	0,992	5,6598E-13
LAMTOR5	1,6752E-10	0,55133893	0,727	0,419	2,8162E-06
NDUFB3	4,9621E-12	0,54916214	0,631	0,25	8,3418E-08
DUSP2	3,7799E-13	0,54697647	0,454	0,097	6,3544E-09
NDUFAB1	7,0099E-12	0,54693291	0,651	0,274	1,1784E-07
PTP4A3	8,4011E-11	0,5467354	0,691	0,387	1,4123E-06
ELOC	1,5123E-13	0,54592055	0,651	0,242	2,5424E-09
PTPRO	9,8734E-17	0,54573388	0,446	0,048	1,6598E-12
DNAJB1	5,4072E-09	0,54485642	0,775	0,476	9,0901E-05
BTF3	4,2797E-11	0,542631	0,851	0,548	7,1946E-07
VAV3	4,2144E-15	0,54168217	0,618	0,185	7,0848E-11
EIF3H	1,1016E-12	0,53905012	0,859	0,508	1,852E-08
TMEM258	7,6006E-10	0,538365	0,896	0,677	1,2777E-05

RPL6	5,1946E-21	0,53662933	1	0,992	8,7327E-17
TRMT112	3,7399E-11	0,53400484	0,847	0,532	6,2871E-07
MYC	4,092E-08	0,52952611	0,715	0,435	0,00068791
NDUFB10	2,41E-09	0,52948949	0,715	0,387	4,0515E-05
RPS29	1,7404E-26	0,5282102	1	1	2,9258E-22
CYB5B	1,8826E-09	0,5275159	0,799	0,548	3,1648E-05
SNHG7	8,7363E-11	0,52636946	0,635	0,298	1,4687E-06
ATP5ME	1,609E-14	0,526208	0,98	0,903	2,7049E-10
HMGN1	4,9419E-12	0,5247091	0,888	0,573	8,3078E-08
LRRC75A	1,7964E-05	0,52257335	0,61	0,395	0,30198944
MAOA	3,9212E-11	0,52204624	0,506	0,161	6,5919E-07
NCOA7	1,0147E-08	0,52144138	0,731	0,444	0,00017058
HNRNPM	8,5378E-11	0,52015369	0,699	0,339	1,4353E-06
COX7C	9,1944E-13	0,51878426	0,968	0,79	1,5457E-08
GZMB	4,3937E-10	0,51514039	0,703	0,355	7,3863E-06
RPL36A	7,7431E-35	0,51447938	1	1	1,3017E-30
EGR1	6,5729E-11	0,51356854	0,398	0,089	1,105E-06
COX4I1	2,4605E-12	0,51219106	0,992	0,919	4,1363E-08
RPL36AL	3,0899E-09	0,50784639	0,823	0,548	5,1944E-05
MRPL51	2,732E-09	0,5057588	0,622	0,315	4,5928E-05
COX7A2	3,1964E-10	0,5029852	0,948	0,75	5,3734E-06
HSPE1	6,991E-09	0,49902428	0,956	0,831	0,00011753
COMMD6	7,5659E-08	0,49641645	0,904	0,742	0,0012719
PDCD5	6,311E-10	0,49504592	0,787	0,476	1,0609E-05
RPS14	1,4205E-16	0,49494672	1	0,992	2,388E-12
ODC1	8,3618E-10	0,49454465	0,562	0,226	1,4057E-05
UQCR11	1,9545E-11	0,49247282	0,98	0,919	3,2857E-07
RPL14	1,2032E-21	0,49040879	1	1	2,0228E-17
NME1	4,2671E-10	0,48920018	0,739	0,403	7,1734E-06
COX5A	9,6942E-10	0,48780093	0,743	0,411	1,6297E-05
RPL37A	2,5397E-37	0,48730657	1	1	4,2696E-33
ID3	1,7293E-08	0,48659076	0,618	0,298	0,0002907
MGST1	2,5569E-11	0,48518073	0,53	0,169	4,2984E-07
MRPS23	2,3229E-12	0,4846922	0,506	0,137	3,9051E-08
RPS7	2,7097E-22	0,48396275	1	1	4,5554E-18
SNRPG	1,626E-07	0,48248086	0,755	0,508	0,00273349
TATDN1	6,9481E-13	0,48035187	0,474	0,105	1,168E-08
LSM3	6,1115E-11	0,4789519	0,546	0,185	1,0274E-06
CKS2	1,9616E-11	0,47890541	0,506	0,161	3,2977E-07
PAM16	3,7614E-10	0,47831619	0,566	0,218	6,3233E-06
LSM7	1,5185E-10	0,47822797	0,976	0,911	2,5527E-06
POMP	1,2358E-07	0,47606339	0,723	0,444	0,00207756
MRPL15	2,1897E-10	0,47532107	0,478	0,161	3,6811E-06
HMGB2	5,9358E-09	0,47346606	0,498	0,194	9,9786E-05
SMOC2	1,478E-12	0,47336222	0,398	0,065	2,4846E-08
JUN	4,9849E-08	0,47045062	0,827	0,556	0,00083802
ANAPC11	1,287E-08	0,47027266	0,695	0,387	0,00021636
ILF2	2,6971E-08	0,46535847	0,594	0,306	0,0004534
MT1A	1,4575E-13	0,46515225	0,462	0,089	2,4502E-09
TRIL	4,839E-12	0,46367966	0,494	0,129	8,1348E-08
MT1E	8,4071E-08	0,46264287	0,787	0,548	0,00141332
COX20	4,418E-08	0,45794329	0,659	0,363	0,0007427

HNRNPA2B1	2,9846E-08	0,45782975	0,956	0,815	0,00050174
RPS27	2,9037E-12	0,45726211	1	1	4,8814E-08
RPS2	6,3474E-23	0,45675746	1	1	1,0671E-18
YBX1	2,555E-08	0,45664482	0,763	0,468	0,00042953
CIAO2B	1,7416E-08	0,45560184	0,719	0,411	0,00029278
PSMA2	9,101E-08	0,453626	0,683	0,379	0,00152997
DMAC1	1,093E-08	0,453626	0,643	0,315	0,00018375
SLC12A2	6,6985E-08	0,4531347	0,855	0,621	0,00112609
NDUFB2	6,04E-08	0,4523006	0,699	0,395	0,00101539
SELENOW	2,3657E-07	0,45194355	0,88	0,685	0,00397693
CPSF6	6,2365E-11	0,45172396	0,434	0,105	1,0484E-06
MAGOH	4,978E-09	0,45098289	0,49	0,177	8,3685E-05
APRT	4,694E-07	0,44987386	0,843	0,685	0,00789115
ZBTB8OS	3,4166E-11	0,44903289	0,478	0,129	5,7436E-07
HMGN3	5,7136E-10	0,4463212	0,478	0,153	9,6052E-06
CLNS1A	5,9153E-11	0,44611194	0,462	0,129	9,9443E-07
HSPA1B	1,4896E-05	0,44499999	0,763	0,565	0,25041326
ATP5PD	6,4477E-10	0,44444657	0,643	0,29	1,0839E-05
AP2S1	9,2853E-09	0,44258383	0,526	0,21	0,0001561
EXOSC7	1,4195E-10	0,44234019	0,442	0,113	2,3864E-06
COX6C	4,4006E-11	0,44229686	0,996	0,935	7,3978E-07
RPL30	4,9611E-29	0,44121542	1	1	8,3401E-25
MRPS33	3,7269E-12	0,43985784	0,41	0,073	6,2652E-08
COQ8A	1,6052E-07	0,4384438	0,546	0,25	0,00269843
POLR2K	3,796E-08	0,43546342	0,775	0,484	0,00063814
NDUFB11	7,6482E-08	0,43512175	0,763	0,476	0,00128574
UQCC2	5,4684E-08	0,43135028	0,647	0,339	0,00091929
ATP5MG	2,5017E-10	0,43005035	0,876	0,565	4,2057E-06
GABARAPL2	1,0177E-08	0,42842544	0,614	0,29	0,00017108
PPIA	7,0933E-14	0,42627032	1	1	1,1925E-09
ENY2	9,4026E-08	0,42574696	0,779	0,492	0,00158067
BANF1	2,8801E-08	0,42570892	0,663	0,347	0,00048418
HNRNPA1	7,8275E-08	0,42393838	0,948	0,798	0,00131589
TMEM14B	7,1279E-12	0,4236393	0,43	0,089	1,1983E-07
PRDX3	1,2855E-09	0,42303768	0,446	0,129	2,1611E-05
CSKMT	2,0547E-09	0,42275532	0,474	0,153	3,4542E-05
DDT	5,9015E-07	0,42205592	0,554	0,298	0,00992106
SNHG15	2,5012E-09	0,41809445	0,438	0,129	4,2048E-05
RPS6	1,2406E-26	0,4175271	1	1	2,0856E-22
LDHB	2,6181E-06	0,41592653	0,755	0,524	0,04401242
PIN4	8,8604E-08	0,41589561	0,562	0,266	0,00148952
PTRH2	2,7671E-09	0,41579855	0,394	0,105	4,6517E-05
FCGRT	2,3434E-05	0,41242016	0,735	0,54	0,39394894
LSM2	1,6729E-06	0,40820687	0,594	0,331	0,02812358
SNRPF	1,371E-07	0,40815161	0,574	0,274	0,00230473
DNPH1	2,0267E-07	0,40804496	0,478	0,202	0,00340709
MUC1	2,3617E-09	0,4069759	0,522	0,194	3,9703E-05
PPIH	3,2911E-12	0,40645479	0,365	0,048	5,5327E-08
SLIRP	3,6297E-07	0,40645479	0,691	0,403	0,00610194
DNAJC19	6,0374E-09	0,40562062	0,486	0,169	0,0001015
UXT	3,8607E-08	0,40545024	0,687	0,371	0,00064903
UQCRQ	5,2263E-07	0,40536233	0,976	0,903	0,00878594

MRPL37	1,2424E-09	0,40530617	0,414	0,113	2,0885E-05
MCM3	1,4454E-08	0,4045705	0,498	0,185	0,00024298
RBBP4	7,8159E-09	0,40428266	0,462	0,153	0,00013139
HOXB8	1,2188E-09	0,40395694	0,398	0,097	2,0489E-05
RPL17	5,4256E-16	0,40368509	1	1	9,1211E-12
FOS	8,6644E-07	0,40309087	0,643	0,355	0,01456568
SNHG12	5,0783E-09	0,40273031	0,41	0,121	8,5372E-05
POLR2I	7,0689E-07	0,4021623	0,622	0,339	0,01188354
UQCR10	5,0847E-06	0,39966317	0,707	0,476	0,08547815
NDUFA12	2,3764E-08	0,39755007	0,522	0,21	0,00039949
SNRPC	3,9619E-08	0,39561651	0,631	0,315	0,00066604
RPA3	4,7074E-09	0,39519404	0,446	0,145	7,9136E-05
NDUFB9	2,6628E-07	0,39497789	0,863	0,629	0,00447648
SEPHS2	1,6337E-07	0,39459653	0,522	0,226	0,00274637
MRPL18	1,0908E-08	0,39144256	0,49	0,177	0,00018338
LYRM1	1,169E-10	0,39122393	0,349	0,056	1,9652E-06
NTHL1	2,132E-07	0,39071179	0,446	0,177	0,00358403
RPL36	1,4957E-18	0,38996185	1	1	2,5144E-14
CFTR	5,0598E-09	0,38891189	0,434	0,129	8,5061E-05
HSPA8	3,1948E-06	0,38882567	0,582	0,323	0,05370821
KIF12	2,1651E-11	0,38859211	0,345	0,048	3,6398E-07
PSME2	1,0925E-08	0,38810859	0,61	0,282	0,00018366
CENPX	3,9898E-06	0,38803766	0,618	0,363	0,06707294
SNHG3	1,252E-07	0,38785823	0,494	0,202	0,00210475
RBM8A	2,7757E-07	0,38773081	0,562	0,266	0,00466622
MIR200CHG	1,1288E-07	0,38742751	0,482	0,194	0,00189762
GINS2	2,0418E-10	0,38703613	0,345	0,056	3,4325E-06
DDX5	7,1407E-05	0,38605066	0,843	0,677	1
RPL32	1,6296E-20	0,38563823	1	1	2,7395E-16
FABP6	5,8959E-12	0,38488806	0,309	0,024	9,9116E-08
SLPI	7,1338E-05	0,38473092	0,582	0,403	1
TOP2A	2,5553E-08	0,38465385	0,462	0,161	0,00042957
NOP10	4,6598E-06	0,38323667	0,534	0,274	0,07833533
METTL26	5,6986E-06	0,38259675	0,791	0,548	0,09579899
SARS	1,3913E-07	0,38246497	0,602	0,298	0,00233893
CDKN1B	2,3082E-07	0,38179865	0,434	0,161	0,00388036
TRABD2A	2,6465E-06	0,38175114	0,494	0,242	0,04449027
PFDN5	9,9265E-07	0,38159631	0,932	0,79	0,01668748
COA4	1,2281E-08	0,3812175	0,45	0,153	0,00020646
EIF3K	2,0026E-06	0,37833787	0,61	0,347	0,03366566
PSMD3	9,966E-07	0,37794979	0,57	0,29	0,01675392
HNMT	1,8088E-05	0,37779937	0,538	0,29	0,30407598
PSMG4	1,0494E-08	0,37719805	0,47	0,161	0,00017641
PARK7	1,0023E-05	0,37694767	0,715	0,484	0,16850492
ZNF593	3,4296E-06	0,37654977	0,57	0,306	0,05765468
METTL5	3,7453E-07	0,37628146	0,498	0,218	0,00629629
SSBP1	3,5922E-08	0,37587328	0,598	0,282	0,00060388
RPS18	1,2322E-24	0,37512288	1	1	2,0715E-20
RGL2	4,7795E-07	0,37486626	0,494	0,218	0,00803482
PCBD1	1,0799E-06	0,37461812	0,602	0,323	0,01815414
AP003774.2	2,8038E-09	0,37433709	0,365	0,081	4,7135E-05
SNHG16	5,2297E-07	0,37404245	0,538	0,25	0,00879165

SRSF2	2,8382E-06	0,37380982	0,667	0,395	0,04771246
NAA38	8,9478E-07	0,372706	0,59	0,306	0,01504214
POLR2G	1,6507E-06	0,372706	0,442	0,185	0,02775045
MZT2A	1,8519E-07	0,37169216	0,759	0,468	0,00311321
RPL5	3,1644E-14	0,37053391	1	1	5,3197E-10
NEDD8	1,708E-06	0,37006128	0,57	0,298	0,02871369
NDUFC1	1,4034E-06	0,36893917	0,61	0,331	0,02359219
DANCR	1,618E-07	0,36887414	0,43	0,153	0,00272001
CNPY2	3,7969E-06	0,3683596	0,618	0,355	0,06382944
CKLF	1,8389E-08	0,36800921	0,41	0,121	0,00030915
DCTPP1	1,914E-07	0,3674829	0,442	0,161	0,00321754
SATB2-AS1	5,3871E-08	0,3671064	0,386	0,113	0,00090562
MUC5B	4,3184E-05	0,36656541	0,47	0,242	0,72596282
PRKDC	1,6246E-06	0,36637066	0,494	0,226	0,02731185
RSRP1	1,4906E-09	0,36545019	0,41	0,105	2,5058E-05
SRPK1	4,5461E-06	0,36480441	0,53	0,266	0,0764251
KIAA1324	1,0624E-11	0,36380045	0,261	0,008	1,7859E-07
ENTPD8	9,4507E-06	0,36371722	0,498	0,25	0,15887569
POLD2	2,0328E-06	0,36314265	0,542	0,29	0,03417344
AL390719.2	6,8449E-09	0,36273342	0,353	0,081	0,00011507
SRSF10	6,7056E-06	0,36258178	0,534	0,29	0,11272752
OTUD6B-AS1	9,8655E-10	0,36216984	0,474	0,153	1,6585E-05
CPNE7	1,3407E-07	0,36040303	0,486	0,194	0,00225377
PDCD6	1,8962E-06	0,36040303	0,478	0,218	0,03187709
CKS1B	3,766E-06	0,36024503	0,506	0,242	0,06331086
CNOT1	3,212E-06	0,35996689	0,57	0,298	0,05399623
RPL24	1,4357E-11	0,35950046	1	1	2,4136E-07
SPAG1	8,9868E-08	0,35906908	0,382	0,113	0,00151078
SNHG19	3,5417E-06	0,35831491	0,478	0,226	0,05953882
SUB1	1,9299E-06	0,35818653	0,582	0,315	0,03244356
ATP5MF	9,736E-06	0,35799322	0,9	0,734	0,16367248
HOXB6	9,2755E-08	0,357885	0,406	0,129	0,0015593
REX1BD	2,375E-05	0,35722768	0,562	0,339	0,39926465
TSTA3	2,6685E-05	0,35634476	0,622	0,379	0,44860667
PCNA	4,3024E-05	0,3560152	0,703	0,468	0,72328452
MPC2	7,6241E-07	0,35565084	0,41	0,161	0,01281689
UFC1	6,7814E-07	0,35478409	0,526	0,242	0,01140024
NOB1	4,9748E-07	0,35459662	0,442	0,169	0,00836313
FOPNL	4,7817E-07	0,35395929	0,398	0,137	0,00803849
COX7B	4,1752E-07	0,35301157	0,863	0,621	0,00701886
HDDC2	1,5525E-07	0,35294275	0,454	0,169	0,00260983
NDUFB6	5,2589E-07	0,35245469	0,462	0,194	0,00884072
MT1X	3,0783E-05	0,35238602	0,494	0,282	0,51749454
SLC39A11	2,6432E-06	0,3519794	0,45	0,194	0,04443494
AIG1	1,2449E-07	0,35174638	0,414	0,137	0,00209282
SMIM26	3,9638E-07	0,35147358	0,835	0,573	0,00666348
PHB	2,7798E-05	0,35145555	0,767	0,54	0,46731696
PRMT7	4,0084E-09	0,3506797	0,333	0,065	6,7385E-05
ARL16	1,6436E-06	0,35049805	0,365	0,129	0,02763086
NSA2	1,3204E-05	0,35047398	0,51	0,258	0,22197074
MRPL27	4,5698E-06	0,35019641	0,494	0,234	0,07682332
NPM1	9,831E-06	0,34973808	0,847	0,637	0,16526919

AC010642.2	1,0928E-08	0,34919429	0,365	0,089	0,00018371
MLXIPL	7,1232E-07	0,34919429	0,345	0,105	0,0119748
NUDT21	1,0779E-06	0,34911089	0,43	0,169	0,01812063
GGCT	2,4174E-06	0,34911089	0,414	0,169	0,04063905
XBP1	4,0626E-07	0,34867305	0,434	0,161	0,0068297
RPL7A	2,2784E-14	0,34756753	1	1	3,8303E-10
PRPS2	1,0127E-06	0,34692852	0,353	0,113	0,01702399
TRAP1	5,2964E-05	0,34655715	0,647	0,411	0,89037747
RIDA	1,1904E-07	0,34649612	0,402	0,129	0,00200117
PCP4	4,4631E-07	0,3460757	0,39	0,129	0,00750286
SFPQ	1,3375E-06	0,34469163	0,622	0,339	0,02248502
CUTA	8,7565E-06	0,34443675	0,767	0,524	0,14720489
NDUFV3	7,4784E-06	0,34434235	0,498	0,242	0,12571913
RBBP6	4,4557E-08	0,34429315	0,386	0,113	0,00074905
CAMTA1	4,1999E-07	0,34429315	0,39	0,129	0,00706039
SERBP1	9,4873E-05	0,34283981	0,711	0,516	1
EREG	5,333E-06	0,34211768	0,538	0,274	0,08965288
MRPS18C	3,8411E-08	0,34173312	0,337	0,081	0,00064572
SPCS1	3,4879E-05	0,34047233	0,631	0,427	0,58634928
WNK2	1,5231E-05	0,34012179	0,442	0,202	0,25604805
SNU13	4,1773E-07	0,33986593	0,791	0,516	0,00702238
NDUFB4	3,3114E-05	0,33958145	0,622	0,403	0,55668505
COX14	3,979E-05	0,33895318	0,494	0,274	0,66891113
NASP	1,3059E-07	0,3387239	0,454	0,169	0,00219529
RPS15	6,2819E-16	0,33855165	1	1	1,0561E-11
DAD1	1,105E-06	0,3384588	0,373	0,137	0,01857627
UBE2C	3,0357E-06	0,33787206	0,482	0,218	0,05103249
RAB40B	1,9084E-06	0,33755385	0,43	0,177	0,03208264
TCEA3	5,0198E-07	0,33688103	0,341	0,097	0,00843879
EIF3M	2,0492E-05	0,3363475	0,542	0,298	0,34448309
PSMG1	1,3775E-07	0,33630036	0,39	0,121	0,00231572
TRAF5	6,167E-06	0,3354642	0,502	0,242	0,10367292
RPS3	7,5848E-11	0,33544203	1	1	1,2751E-06
TMEM70	1,0267E-07	0,33495415	0,357	0,097	0,00172592
LSM6	5,8162E-09	0,33377034	0,301	0,048	9,7776E-05
NDUFS6	0,00011757	0,33319599	0,494	0,29	1
IFT43	1,7126E-08	0,33299629	0,361	0,089	0,0002879
FOXP4-AS1	1,7548E-09	0,33275315	0,369	0,089	2,9499E-05
NOP56	1,7071E-05	0,33221218	0,707	0,46	0,28697914
SNHG6	4,2266E-05	0,33187482	0,956	0,863	0,71052769
CDCA7	9,0799E-09	0,33169528	0,281	0,04	0,00015264
DNAJA3	4,1383E-06	0,33142973	0,438	0,185	0,06956968
LRP4	3,596E-07	0,33084398	0,462	0,185	0,00604524
CYB5A	5,4423E-08	0,33079052	0,349	0,089	0,0009149
MIEN1	1,8399E-06	0,33071985	0,602	0,323	0,03093072
PTPMT1	8,0628E-08	0,32917863	0,293	0,056	0,00135544
MYL6B	7,9888E-08	0,32898462	0,321	0,073	0,001343
HOXB-AS3	1,3233E-06	0,32861342	0,341	0,105	0,02224551
SNHG9	1,1911E-05	0,32861342	0,606	0,347	0,20024245
NDUFC2	5,3355E-07	0,32847968	0,639	0,347	0,00896956
SNHG1	1,2679E-07	0,32826304	0,402	0,129	0,00213142
PAQR8	2,513E-06	0,32809511	0,402	0,161	0,04224623

UBA52	1,9639E-09	0,32782204	1	0,984	3,3015E-05
CANT1	7,8818E-05	0,32748873	0,554	0,323	1
RNF7	0,0001138	0,32739359	0,679	0,476	1
LAGE3	5,5135E-07	0,32692923	0,293	0,073	0,00926872
RPS19	7,8862E-15	0,32691221	1	1	1,3258E-10
MRPS24	2,5901E-05	0,32690231	0,474	0,234	0,43542675
TP53RK	1,1261E-07	0,32676972	0,341	0,089	0,00189303
RNF43	0,00031552	0,32676972	0,767	0,589	1
ALG13	2,2513E-07	0,32661485	0,349	0,097	0,00378471
MRPL16	3,0371E-06	0,32590349	0,402	0,153	0,05105633
ENSA	0,0002687	0,32557395	0,687	0,508	1
DHX9	2,0836E-05	0,32552117	0,438	0,202	0,35027097
TMEM256	1,9745E-06	0,32540029	0,627	0,347	0,03319357
LAMTOR4	1,9804E-06	0,32540029	0,811	0,556	0,03329212
TMEM141	4,6011E-06	0,32528247	0,888	0,685	0,07734873
YRDC	9,523E-09	0,32497339	0,281	0,04	0,00016009
PRMT5	1,7924E-07	0,32470997	0,313	0,073	0,0030132
NRARP	3,7846E-06	0,32411526	0,378	0,137	0,06362359
GTF2H5	6,7362E-06	0,3237964	0,438	0,194	0,11324181
EEF1B2	9,7104E-07	0,32322936	0,992	0,952	0,01632415
RBMX	0,00033541	0,32259197	0,542	0,363	1
COTL1	3,6973E-06	0,32184368	0,458	0,202	0,0621552
MRPS17	2,8797E-05	0,32184368	0,422	0,202	0,48410506
NDUFAF4	7,8121E-07	0,32029815	0,353	0,113	0,01313296
MRPL36	1,2921E-05	0,31986487	0,446	0,202	0,21720944
UBE2V2	8,7017E-06	0,31955338	0,534	0,274	0,14628345
HMGCS2	0,00038165	0,3194972	0,944	0,847	1
MDH2	0,00015517	0,31943521	0,534	0,315	1
RAN	6,3391E-05	0,31889422	0,683	0,452	1
WDR90	9,1065E-06	0,3181674	0,349	0,129	0,15308939
MBTPS1	1,7155E-05	0,31811103	0,382	0,161	0,28838833
NDUFA13	0,00015492	0,31779905	0,908	0,782	1
CD47	1,5059E-05	0,31769833	0,618	0,363	0,25315528
MCM6	1,0124E-08	0,31612247	0,309	0,056	0,0001702
EIF4EBP3	2,1858E-06	0,31612247	0,365	0,129	0,03674542
ZFAND1	5,796E-05	0,31432919	0,486	0,266	0,97435941
SNRPD3	3,316E-07	0,31428347	0,478	0,202	0,0055746
MRPL58	3,8007E-06	0,31415829	0,41	0,161	0,06389312
WDR77	3,6635E-08	0,31413118	0,414	0,137	0,00061587
RPL26L1	1,3167E-08	0,31398356	0,329	0,073	0,00022136
ECT2	1,6253E-06	0,31398356	0,309	0,089	0,02732239
RPL13A	3,2148E-11	0,31397778	1	1	5,4045E-07
SEC61G	5,7747E-05	0,31383066	0,771	0,548	0,97078496
TUBA1B	0,0001576	0,31381231	0,779	0,573	1
PRR15L	9,8568E-06	0,31337186	0,679	0,419	0,16570188
MRPS34	0,00024408	0,31308202	0,598	0,387	1
EIF1AX	3,7659E-05	0,31304964	0,606	0,363	0,63308566
RANBP1	0,00018947	0,3127239	0,514	0,298	1
RAB6A	1,5017E-06	0,3123209	0,438	0,177	0,02524486
DTYMK	4,5986E-07	0,31210941	0,398	0,137	0,00773075
IARS2	8,0686E-07	0,31210941	0,394	0,137	0,01356415
RSL1D1	0,00030161	0,31143586	0,586	0,387	1

HNRNPDL	0,0004065	0,31076924	0,695	0,492	1
DGUOK	4,1453E-08	0,31073593	0,514	0,218	0,00069687
BOLA3	1,2992E-06	0,31022187	0,41	0,153	0,02184101
CNBP	0,00068053	0,30983621	0,811	0,653	1
FUBP1	2,1904E-06	0,30979022	0,329	0,105	0,03682365
CENPW	1,2119E-06	0,3096962	0,313	0,089	0,02037277
LUC7L3	0,0002442	0,30954982	0,546	0,331	1
ACP1	2,1051E-05	0,3094741	0,574	0,323	0,35389425
TOMM5	3,6404E-05	0,30939661	0,699	0,46	0,61198426
RBX1	1,1354E-05	0,30906772	0,522	0,266	0,19087829
IARS	4,9305E-06	0,308705	0,486	0,226	0,08288616
NDUFA1	0,00077738	0,30830297	0,783	0,621	1
ERI3	5,0861E-06	0,30785486	0,349	0,121	0,08550172
TSPAN12	2,159E-06	0,30761097	0,313	0,089	0,03629529
APMAP	1,2274E-05	0,30745761	0,53	0,274	0,20633364
ETFB	2,7703E-05	0,30724432	0,502	0,258	0,4657151
OCIAD1	0,00016938	0,30613838	0,554	0,339	1
SRSF1	3,4971E-05	0,30590263	0,526	0,282	0,58789716
DNMT1	9,0976E-08	0,30570586	0,349	0,097	0,0015294
TRIR	0,00015795	0,30555323	0,807	0,613	1
USP25	2,9161E-08	0,30506728	0,285	0,048	0,00049022
MT1H	8,2328E-08	0,30489522	0,245	0,032	0,00138401
TSPAN8	0,00021012	0,30444926	0,811	0,613	1
CCHCR1	3,1772E-06	0,30437727	0,422	0,169	0,05341121
BROX	1,2909E-05	0,30404964	0,373	0,145	0,21700972
TOMM20	8,8764E-05	0,30301359	0,667	0,435	1
AC078993.1	2,2203E-07	0,30294708	0,265	0,048	0,00373249
RPF1	7,9402E-08	0,30274344	0,277	0,048	0,00133483
WDR83OS	0,00106885	0,30270196	0,667	0,484	1
EEF1E1	1,2332E-05	0,30231667	0,386	0,153	0,20732164
CSE1L	6,7057E-05	0,30197829	0,506	0,274	1
EMP2	2,4244E-07	0,30172625	0,365	0,113	0,00407568
PTCH1	6,9625E-06	0,30172625	0,361	0,137	0,1170467
PRDX2	2,9067E-05	0,30150044	0,767	0,532	0,48865267
CRNDE	2,0844E-07	0,30130376	0,333	0,089	0,00350406
PYGB	0,00281364	0,30085572	0,691	0,524	1
H3F3A	1,5561E-05	0,30079082	0,976	0,887	0,26160058
SPINT1-AS1	3,5732E-07	0,30062138	0,277	0,056	0,00600696
PSMB2	7,2988E-05	0,30029751	0,518	0,29	1
HOXB9	4,2888E-05	0,30022485	0,369	0,153	0,72099525
ASCL2	1,112E-05	0,30000281	0,707	0,452	0,18693238
MT-CO2	0,01523706	-0,3007441	0,996	1	1
FAM50A	1,4528E-05	-0,301428	0,269	0,46	0,24423322
RAB1B	2,6396E-05	-0,3024637	0,241	0,395	0,44373857
HLA-E	8,5288E-05	-0,3031212	0,466	0,54	1
PRSS8	0,00118843	-0,3035358	0,602	0,742	1
ARHGDIA	0,00040774	-0,3037105	0,534	0,702	1
JAG1	8,3634E-05	-0,3047726	0,201	0,363	1
INTS1	0,00010741	-0,3048193	0,285	0,484	1
RAC1	0,00081468	-0,3053659	0,55	0,637	1
HPGD	2,1082E-05	-0,3053659	0,088	0,25	0,35440685
HLA-C	8,6471E-06	-0,3059532	0,787	0,774	0,14536588

CD164	7,124E-05	-0,3060227	0,723	0,75	1
DAPK3	7,4005E-05	-0,3065597	0,201	0,355	1
AQP8	1,0509E-06	-0,3074613	0,036	0,202	0,01766619
QSOX1	5,9127E-05	-0,3082438	0,237	0,419	0,99399136
MVP	0,00063617	-0,3109905	0,478	0,645	1
CDC34	0,0001177	-0,311055	0,337	0,508	1
RHOA	1,9924E-05	-0,3114319	0,574	0,637	0,33494705
NAPRT	1,8518E-05	-0,3123964	0,542	0,613	0,31130707
SSR4	0,00109384	-0,3137707	0,859	0,927	1
GNAI2	4,2843E-05	-0,3146957	0,149	0,331	0,72023025
RABAC1	7,0633E-05	-0,31656	0,285	0,508	1
INF2	2,3717E-05	-0,3200801	0,438	0,54	0,39870198
ANKRD37	2,6026E-05	-0,3203162	0,229	0,371	0,43751587
YWHAZ	1,9284E-05	-0,3203386	0,996	0,984	0,32418713
RPS9	3,8123E-05	-0,3203648	0,996	1	0,6408832
CTNNB1	0,00042776	-0,322065	0,462	0,637	1
DCN	1,6207E-06	-0,3224394	0,096	0,282	0,02724562
ABHD2	3,9565E-05	-0,3226113	0,462	0,581	0,66513553
TIMP2	8,2393E-09	-0,3234207	0,072	0,323	0,00013851
ARPC4	0,0002475	-0,323641	0,349	0,508	1
B4GALT1	1,5229E-05	-0,3248741	0,201	0,387	0,25602045
IFNGR2	0,00023	-0,3261732	0,39	0,548	1
SERINC3	0,00027244	-0,3270067	0,458	0,548	1
COLGALT1	0,00015781	-0,3277337	0,325	0,492	1
RNPEPL1	5,8307E-06	-0,3295941	0,237	0,435	0,09802047
PRELP	4,5732E-08	-0,3299346	0,052	0,258	0,0007688
SPTBN1	0,0001174	-0,3318695	0,506	0,629	1
CAVIN1	4,1805E-08	-0,3332088	0,052	0,266	0,00070278
GPR35	0,00057503	-0,3336103	0,426	0,54	1
GRN	1,018E-05	-0,3345335	0,655	0,718	0,1711427
TLE5	8,4928E-05	-0,3349895	0,598	0,718	1
MT-CO3	0,01164807	-0,3356422	0,96	0,984	1
TPT1	8,1187E-13	-0,3359151	1	1	1,3648E-08
FGB	2,1835E-05	-0,3361678	0,221	0,355	0,36706041
COL6A2	1,9791E-05	-0,3361953	0,084	0,242	0,33271326
SPINT2	2,2635E-06	-0,336567	0,855	0,895	0,03805171
LAMC2	5,7836E-06	-0,3443911	0,229	0,371	0,09722888
MT-ND2	0,00020733	-0,345472	0,815	0,895	1
VCAN	1,4907E-06	-0,3462962	0,112	0,298	0,02505936
YWHAE	8,5282E-05	-0,3462962	0,49	0,613	1
FBLN2	2,9124E-06	-0,3468425	0,08	0,258	0,04895967
CXCL16	1,503E-06	-0,353053	0,217	0,427	0,02526729
CD59	8,7245E-06	-0,3531383	0,345	0,484	0,14666736
TMPRSS4	0,0001103	-0,357278	0,49	0,645	1
RHOC	7,8161E-06	-0,3573767	0,462	0,589	0,13139638
APP	5,5893E-06	-0,3581943	0,711	0,782	0,09396222
SQSTM1	7,633E-05	-0,3610337	0,739	0,815	1
CDX2	0,00013318	-0,3626991	0,606	0,718	1
CYBA	0,00010421	-0,3651337	0,414	0,556	1
SERPINH1	3,8863E-06	-0,365348	0,253	0,476	0,06533245
ARFGAP1	6,5478E-05	-0,3670719	0,373	0,532	1
DHRS3	1,2977E-05	-0,3674434	0,333	0,516	0,21814939

IGFBP5	4,7729E-08	-0,3701234	0,092	0,339	0,00080237
LAMA3	3,4631E-09	-0,3708024	0,06	0,298	5,8218E-05
WDR1	1,5895E-08	-0,3740489	0,542	0,581	0,0002672
ZNF703	0,00010967	-0,3742427	0,47	0,621	1
A2M	1,4453E-05	-0,3750394	0,133	0,306	0,24296901
ORM1	1,5243E-07	-0,3753791	0,096	0,323	0,00256245
MICAL1	1,4122E-06	-0,3773645	0,257	0,452	0,02373967
ADM	2,6173E-08	-0,3782512	0,088	0,339	0,00043999
EMP1	1,4741E-07	-0,3791739	0,12	0,347	0,00247804
TRIM31	4,4877E-07	-0,3802011	0,269	0,435	0,00754424
REN	7,7154E-11	-0,3803641	0,016	0,226	1,297E-06
TPI1	9,7689E-06	-0,3811106	0,735	0,831	0,16422504
AHNAK	2,6711E-06	-0,3820239	0,313	0,484	0,04490455
TIMP3	8,5774E-07	-0,382369	0,141	0,339	0,01441942
DDR1	4,3156E-05	-0,382369	0,546	0,702	0,72548975
EEF2	1,6063E-07	-0,3846455	0,984	1	0,00270039
MUC13	4,1222E-06	-0,3872055	0,631	0,734	0,06929822
MT2A	0,00070376	-0,3879785	0,747	0,863	1
TDP2	5,9031E-07	-0,3891343	0,177	0,419	0,00992378
COL6A3	2,5963E-10	-0,3931514	0,056	0,306	4,3647E-06
DNM2	5,0216E-05	-0,3946592	0,478	0,653	0,84418185
AUP1	9,3544E-10	-0,3968262	0,526	0,581	1,5726E-05
HDLBP	2,5421E-07	-0,4002462	0,442	0,597	0,00427353
SOX4	2,0673E-06	-0,4008684	0,458	0,661	0,03475355
C1S	1,7536E-09	-0,4021586	0,092	0,339	2,9481E-05
AMIGO2	4,3477E-08	-0,4027608	0,129	0,363	0,00073088
MAP2K2	7,9409E-06	-0,4035651	0,357	0,613	0,13349527
PLAUR	2,2958E-09	-0,4045413	0,072	0,29	3,8594E-05
SPINK1	6,8493E-06	-0,4057362	0,526	0,669	0,1151444
ANO9	1,7499E-06	-0,4090899	0,61	0,726	0,02941703
CDH17	1,1191E-05	-0,4091613	0,876	0,911	0,18812924
CFL1	1,0628E-06	-0,4109277	0,92	0,96	0,01786644
ENTPD2	1,9934E-07	-0,4112571	0,193	0,435	0,00335114
CYP2S1	2,2013E-07	-0,4124309	0,337	0,548	0,00370061
ECE1	3,0958E-08	-0,4137537	0,221	0,435	0,00052043
RTN4	8,7966E-07	-0,4138397	0,51	0,661	0,01478789
MMP11	4,7919E-08	-0,4156254	0,108	0,339	0,00080557
LUM	2,0155E-08	-0,4182738	0,12	0,371	0,00033882
GGH	7,5678E-07	-0,4208431	0,622	0,726	0,01272217
ACADVL	1,7005E-06	-0,4208431	0,514	0,694	0,02858722
HLA-DRB1	8,1356E-08	-0,4208431	0,08	0,29	0,00136767
ZYX	8,4479E-07	-0,4230208	0,281	0,532	0,01420173
LRP10	2,6783E-08	-0,423337	0,157	0,387	0,00045025
C1R	8,3018E-09	-0,4260798	0,092	0,347	0,00013956
EZR	2,6802E-07	-0,4263058	0,783	0,871	0,00450562
PIM1	1,1197E-06	-0,4274459	0,269	0,484	0,01882333
HLA-DRA	1,4226E-07	-0,4284163	0,124	0,363	0,00239155
IGFBP3	1,204E-10	-0,4318142	0,052	0,306	2,024E-06
FSTL1	1,0513E-08	-0,4361099	0,12	0,379	0,00017673
ITGB1	1,882E-07	-0,4389147	0,361	0,565	0,00316382
PRR15	8,8374E-08	-0,440782	0,663	0,782	0,00148565
SERPING1	6,3216E-09	-0,4414534	0,116	0,347	0,00010627

PKM	1,5353E-08	-0,4425849	0,574	0,702	0,0002581
HEPH	1,4707E-06	-0,446186	0,45	0,621	0,02472324
RABL6	8,3243E-09	-0,4468023	0,386	0,573	0,00013994
ST14	2,0945E-08	-0,4469323	0,498	0,661	0,00035211
NORAD	7,621E-08	-0,4481637	0,807	0,895	0,00128117
SAA1	1,6887E-06	-0,4499056	0,313	0,5	0,02838887
PPDPF	4,9314E-08	-0,4505905	0,904	0,952	0,00082901
BSG	8,0734E-10	-0,4525956	0,851	0,879	1,3572E-05
JUP	3,2593E-09	-0,4549689	0,747	0,79	5,4793E-05
LGALS3BP	2,5521E-07	-0,4552162	0,422	0,629	0,00429035
TNIP1	9,916E-09	-0,4556085	0,129	0,403	0,0001667
H1FX	5,9037E-08	-0,455757	0,333	0,565	0,00099248
C4orf48	7,1772E-10	-0,4586865	0,193	0,508	1,2066E-05
TINAGL1	2,6069E-09	-0,4590178	0,257	0,524	4,3824E-05
PABPC1	3,7813E-14	-0,4601514	1	1	6,3568E-10
PHGR1	6,0587E-11	-0,4615583	0,992	1	1,0185E-06
HSP90B1	2,3558E-08	-0,4629072	0,667	0,766	0,00039603
TPM1	1,1649E-07	-0,4645357	0,39	0,589	0,00195838
ENG	5,7055E-10	-0,4647602	0,1	0,371	9,5916E-06
LRRC32	1,0505E-10	-0,466223	0,064	0,331	1,766E-06
BCAP31	1,4523E-09	-0,4727493	0,671	0,806	2,4414E-05
COMP	1,4992E-15	-0,4765526	0,02	0,315	2,5204E-11
UBC	1,671E-09	-0,4796252	0,94	0,992	2,8091E-05
LSR	7,4965E-08	-0,4812021	0,494	0,75	0,00126023
FNDC1	8,1976E-15	-0,4832837	0,044	0,363	1,3781E-10
SPP1	8,6704E-09	-0,4853692	0,149	0,444	0,00014576
IGKC	4,3624E-07	-0,4892929	0,205	0,444	0,00733365
MFGE8	2,3875E-14	-0,4912324	0,044	0,355	4,0137E-10
CTSA	4,2675E-08	-0,4955279	0,635	0,839	0,00071741
BLCAP	1,2138E-10	-0,4973966	0,418	0,597	2,0405E-06
TAX1BP3	3,3685E-10	-0,4976587	0,261	0,548	5,6628E-06
LMNA	6,3159E-08	-0,4995265	0,53	0,742	0,00106177
MYO1C	2,9147E-10	-0,5090703	0,289	0,605	4,8999E-06
PPP1CB	2,5731E-10	-0,5122175	0,442	0,702	4,3256E-06
TNNC2	1,4789E-08	-0,51427	0,502	0,694	0,00024863
HEBP2	3,2503E-10	-0,5152171	0,47	0,685	5,464E-06
DSTN	6,9429E-12	-0,5168122	0,735	0,895	1,1672E-07
MTRNR2L12	8,2421E-11	-0,519407	0,177	0,444	1,3856E-06
APOC3	6,9854E-08	-0,5203788	0,185	0,444	0,00117432
SULF1	9,0854E-14	-0,5214755	0,052	0,363	1,5274E-09
LBH	3,8289E-09	-0,5223337	0,165	0,419	6,4368E-05
THY1	7,0842E-12	-0,5235694	0,08	0,371	1,1909E-07
COL5A1	5,3033E-14	-0,5246789	0,064	0,395	8,9154E-10
ABHD12	2,4066E-10	-0,5276144	0,373	0,589	4,0458E-06
GPX2	3,502E-10	-0,5283142	0,88	0,919	5,8873E-06
HSPG2	2,343E-11	-0,5303566	0,241	0,468	3,9388E-07
MYH14	1,0643E-09	-0,5380267	0,265	0,524	1,7892E-05
SEC61A1	6,9776E-10	-0,5385719	0,442	0,653	1,173E-05
ITGB4	1,2521E-09	-0,5397842	0,438	0,613	2,105E-05
NNMT	7,1559E-13	-0,5416736	0,076	0,379	1,203E-08
CDHR5	1,2118E-10	-0,5432398	0,145	0,427	2,0371E-06
NBL1	6,5457E-10	-0,5445785	0,47	0,653	1,1004E-05

HP	9,0532E-08	-0,5451713	0,169	0,411	0,00152194
MT-CYB	1,6401E-09	-0,5485716	0,88	0,96	2,7572E-05
ATP9A	2,657E-11	-0,5525792	0,261	0,508	4,4667E-07
MT-ND3	1,4387E-06	-0,5526071	0,731	0,831	0,02418549
TPM4	3,6769E-09	-0,5531258	0,518	0,742	6,1813E-05
SLC6A8	1,7725E-16	-0,5553285	0,044	0,387	2,9797E-12
WNT11	1,3148E-10	-0,5555787	0,293	0,581	2,2103E-06
HLA-A	1,5668E-10	-0,5556013	0,622	0,871	2,634E-06
APOE	4,4075E-08	-0,5575318	0,205	0,476	0,00074094
FLNB	2,5753E-12	-0,5664484	0,317	0,597	4,3294E-08
B2M	1,5468E-09	-0,5668309	0,851	0,927	2,6003E-05
TAGLN2	4,0064E-11	-0,5675452	0,795	0,895	6,7351E-07
FSTL3	5,0334E-14	-0,5767613	0,096	0,444	8,4616E-10
PFN1	2,0251E-12	-0,5771752	0,863	0,944	3,4044E-08
GPX4	1,4435E-12	-0,5831145	0,731	0,855	2,4267E-08
CST3	4,1299E-16	-0,5836995	0,984	1	6,9427E-12
CSTB	9,5994E-12	-0,5873823	0,835	0,927	1,6137E-07
PTTG1IP	7,0936E-14	-0,5875798	0,578	0,75	1,1925E-09
SERINC2	2,705E-10	-0,5881942	0,382	0,621	4,5473E-06
DDIT4	1,1603E-10	-0,5935135	0,321	0,645	1,9506E-06
TPM2	3,9518E-12	-0,5939494	0,193	0,508	6,6434E-08
GAPDH	9,4985E-23	-0,5974111	1	1	1,5968E-18
CNN2	2,1642E-13	-0,6081744	0,365	0,645	3,6382E-09
MUC20	1,2748E-11	-0,6096461	0,269	0,597	2,1431E-07
PIGZ	8,9836E-13	-0,6149142	0,257	0,556	1,5102E-08
MYL6	4,3809E-16	-0,6173735	0,871	0,96	7,3648E-12
ELF3	2,3735E-14	-0,6248377	0,855	0,96	3,9901E-10
IGFBP4	8,596E-13	-0,628523	0,305	0,669	1,4451E-08
CALD1	8,816E-17	-0,6310157	0,088	0,452	1,4821E-12
CLDN3	1,7097E-16	-0,6376388	0,964	0,992	2,8741E-12
P4HB	1,9661E-17	-0,6391486	0,779	0,903	3,3052E-13
MISP	9,2206E-14	-0,6423693	0,574	0,774	1,5501E-09
PDXK	1,0266E-13	-0,6447728	0,462	0,718	1,7258E-09
CD63	1,0571E-15	-0,6491437	0,715	0,879	1,7772E-11
SERPINA1	1,8245E-13	-0,6562917	0,241	0,629	3,0671E-09
APOA1	1,2176E-13	-0,6623697	0,225	0,597	2,0468E-09
SDCBP2	2,4196E-17	-0,6668505	0,108	0,492	4,0676E-13
KRT20	3,2442E-13	-0,6671343	0,261	0,573	5,4539E-09
CAPG	3,9012E-15	-0,6702026	0,249	0,589	6,5583E-11
GPCPD1	2,9423E-13	-0,6722495	0,554	0,685	4,9463E-09
C19orf33	3,0917E-12	-0,6739164	0,667	0,839	5,1975E-08
DGAT1	3,276E-18	-0,675657	0,727	0,919	5,5073E-14
MSLN	4,1231E-12	-0,6785637	0,289	0,581	6,9313E-08
THBS2	1,3476E-17	-0,6988797	0,129	0,548	2,2654E-13
CTSB	6,1774E-16	-0,6999972	0,526	0,847	1,0385E-11
H1F0	5,0895E-17	-0,7029776	0,671	0,823	8,5559E-13
CD74	6,6083E-16	-0,7070001	0,157	0,524	1,1109E-11
RRBP1	1,0855E-14	-0,7109875	0,651	0,879	1,8248E-10
DEPP1	4,5548E-16	-0,7123976	0,145	0,484	7,6571E-12
ANXA2	5,9271E-17	-0,7196659	0,522	0,774	9,9641E-13
HLA-B	2,1019E-18	-0,7231527	0,871	0,976	3,5336E-14
CD151	1,2239E-16	-0,7252366	0,53	0,806	2,0575E-12

SORL1	1,6611E-15	-0,7253891	0,313	0,581	2,7924E-11
PLXNB2	5,782E-18	-0,7319442	0,53	0,734	9,7201E-14
APOC1	1,3967E-13	-0,746419	0,217	0,54	2,3479E-09
CD24	7,7622E-25	-0,746639	0,996	1	1,3049E-20
MT-ND1	5,3363E-13	-0,7523573	0,876	0,96	8,9709E-09
SLC44A4	9,5735E-19	-0,7583681	0,382	0,742	1,6094E-14
S100A11	1,0663E-23	-0,7602164	0,972	1	1,7925E-19
CALR	5,5134E-17	-0,763312	0,627	0,879	9,2686E-13
TMBIM1	1,5257E-15	-0,764443	0,365	0,71	2,5648E-11
LRP1	1,0572E-18	-0,7811501	0,237	0,661	1,7773E-14
IL32	5,5595E-19	-0,7893563	0,261	0,677	9,3461E-15
CCND1	1,1709E-17	-0,8003365	0,558	0,806	1,9684E-13
MT-ND4	2,5292E-18	-0,800738	0,936	1	4,2519E-14
BHLHE40	2,9067E-14	-0,8007544	0,229	0,573	4,8864E-10
ALB	2,8056E-14	-0,8022722	0,378	0,71	4,7166E-10
AGPAT2	4,5186E-19	-0,8058681	0,309	0,726	7,5963E-15
LDHA	2,5928E-21	-0,8118799	0,755	0,903	4,3587E-17
CHPF	2,9496E-18	-0,8125595	0,305	0,685	4,9585E-14
MT-ATP6	2,75E-15	-0,8212203	0,892	0,992	4,623E-11
ACTN4	6,6786E-21	-0,8269431	0,622	0,887	1,1227E-16
MYH9	8,729E-23	-0,8296487	0,534	0,79	1,4674E-18
EPCAM	1,2326E-31	-0,8311842	0,996	1	2,0722E-27
MALAT1	8,4126E-24	-0,8571469	0,819	0,992	1,4142E-19
HSPB1	9,851E-22	-0,862611	0,763	0,887	1,6561E-17
VEGFA	5,052E-19	-0,8763291	0,562	0,806	8,4928E-15
PSAP	1,1341E-26	-0,8800314	0,643	0,935	1,9065E-22
MTMR11	6,5733E-21	-0,8843421	0,273	0,637	1,105E-16
ACTA2	2,5155E-20	-0,8911631	0,133	0,581	4,2288E-16
KRT18	1,0446E-35	-0,9055263	1	1	1,756E-31
TGFBI	2,9829E-23	-0,9473593	0,45	0,831	5,0146E-19
CTSD	6,3143E-24	-0,9801407	0,394	0,831	1,0615E-19
TSPAN1	2,1773E-21	-0,9890299	0,321	0,726	3,6603E-17
COL4A1	4,2771E-25	-1,0107719	0,141	0,621	7,1902E-21
ATP1B1	1,9078E-25	-1,0148792	0,241	0,726	3,2073E-21
LGALS1	5,0142E-28	-1,0418106	0,092	0,613	8,4294E-24
VIM	3,3077E-27	-1,0446684	0,141	0,661	5,5606E-23
GPRC5A	2,5353E-25	-1,0478474	0,39	0,79	4,262E-21
PLEC	1,2478E-32	-1,0557574	0,386	0,855	2,0978E-28
GSN	1,7949E-30	-1,0622048	0,193	0,726	3,0175E-26
COL4A2	1,1229E-26	-1,0745184	0,129	0,637	1,8878E-22
COL3A1	1,6229E-27	-1,098346	0,221	0,71	2,7283E-23
AEBP1	4,5474E-30	-1,098915	0,221	0,742	7,6446E-26
GNAS	8,493E-41	-1,1014302	0,871	0,992	1,4278E-36
TMSB4X	6,6148E-51	-1,1253223	0,996	1	1,112E-46
MT-CO1	1,3372E-28	-1,1545752	0,863	1	2,248E-24
COL18A1	1,3739E-26	-1,1689708	0,177	0,702	2,3097E-22
FLNA	1,004E-34	-1,1944927	0,353	0,847	1,6878E-30
S100A6	3,3722E-61	-1,1980092	1	1	5,669E-57
MYL9	3,1467E-36	-1,2028425	0,233	0,839	5,2899E-32
SLC2A1	5,4057E-30	-1,2074395	0,293	0,766	9,0876E-26
NDRG1	4,2582E-32	-1,2607038	0,249	0,742	7,1584E-28
MGP	7,4032E-36	-1,3267874	0,165	0,766	1,2445E-31

TAGLN	1,6811E-39	-1,3428406	0,165	0,798	2,8261E-35
SCD	2,0434E-41	-1,3717037	0,494	0,927	3,4351E-37
PMEPA1	5,0885E-39	-1,3894527	0,181	0,806	8,5542E-35
COL1A1	6,4556E-41	-1,3946116	0,213	0,855	1,0853E-36
FXYD3	1,5617E-48	-1,4117578	0,795	0,96	2,6254E-44
SDC4	1,7421E-46	-1,4391857	0,61	0,96	2,9287E-42
POSTN	5,693E-41	-1,4512168	0,096	0,758	9,5705E-37
COL1A2	2,8228E-41	-1,4856346	0,241	0,855	4,7454E-37
KRT8	5,2632E-69	-1,5248518	0,988	1	8,8479E-65
ACTB	1,2129E-77	-1,5374187	1	1	2,0391E-73
SPARC	2,3461E-45	-1,5558222	0,349	0,927	3,9441E-41
BGN	2,8651E-40	-1,5589594	0,213	0,815	4,8165E-36
TFF3	2,3661E-49	-1,6246917	0,952	0,992	3,9777E-45
KRT19	8,2645E-76	-1,6792805	0,92	1	1,3893E-71
IGFBP7	2,2996E-43	-1,7014342	0,394	0,944	3,8659E-39
CEACAM6	1,6665E-73	-1,8149995	0,478	0,984	2,8016E-69
EFNA1	1,3991E-79	-1,947954	0,904	0,992	2,3521E-75
TIMP1	7,6726E-68	-2,2487006	0,534	0,992	1,2898E-63
FN1	4,807E-66	-2,2838794	0,245	0,935	8,081E-62
CKB	6,4839E-63	-2,35528	0,454	0,984	1,09E-58
FABP1	1,3223E-60	-2,3833817	0,289	0,927	2,223E-56
