# Supplementary material for: Spatial resolution of cellular senescence dynamics in human colorectal liver metastasis
Source: Aging Cell. 2023 May 8;22(7):e13853. doi: 10.1111/acel.13853 (PMC10352575; doi:10.1111/acel.13853)
Supplement: Supplementary file 10 — Table S4 [file ACEL-22-e13853-s011.pdf]

Supplementary TableS4 eSMCCs pathways

Term	Pathway	Overlap	Adjusted.P.valı	Odds.Ratio
GO:0002181	Ribosome biogenesis and translation	53/93	1,17196E-59	64,2888682
GO:0006412	Ribosome biogenesis and translation	68/214	4,87706E-56	23,3466426
GO:0006614	Ribosome biogenesis and translation	50/90	8,45974E-56	60,2006173
GO:0045047	Ribosome biogenesis and translation	52/103	2,77548E-55	49,3206831
GO:0006613	Ribosome biogenesis and translation	50/94	1,05239E-54	54,7166105
GO:0000184	Ribosome biogenesis and translation	52/113	1,04315E-52	41,2141724
GO:0043043	Ribosome biogenesis and translation	58/162	2,36525E-51	27,3100659
GO:0000956	Ribosome biogenesis and translation	56/171	4,85166E-47	23,713196
GO:0034645	Ribosome biogenesis and translation	66/314	3,7844E-42	13,2017788
GO:0010467	Ribosome biogenesis and translation	66/356	1,50096E-38	11,2652247
GO:0044267	Ribosome biogenesis and translation	64/417	3,1856E-32	8,89915449
GO:0006364	Ribosome biogenesis and translation	44/173	2,17036E-31	16,113167
GO:0042254	Ribosome biogenesis and translation	45/192	1,76694E-30	14,483325
GO:0016072	Ribosome biogenesis and translation	41/162	3,96829E-29	15,8977922
GO:0034470	Ribosome biogenesis and translation	41/201	3,67818E-25	11,9985658
GO:0019646	Aerobic metabolism	26/70	5,43483E-23	26,8608815
GO:0042775	Aerobic metabolism	26/71	7,90367E-23	26,2626263
GO:0022618	Ribosome biogenesis and translation	31/136	2,24271E-20	13,5363881
GO:0000398	mRNA processing	37/274	2,70892E-16	7,21131367
GO:0033108	Aerobic metabolism	22/90	5,75493E-15	14,5528461
GO:0006120	Aerobic metabolism	16/39	9,17674E-15	30,9351292
GO:0032981	Aerobic metabolism	18/58	3,36918E-14	20,0852403
GO:0010257	Aerobic metabolism	18/58	3,36918E-14	20,0852403
GO:0000377	mRNA processing	32/251	2,40248E-13	6,67586386
GO:0042274	Ribosome biogenesis and translation	15/40	3,58577E-13	26,6181818
GO:0042273	Ribosome biogenesis and translation	17/57	4,22389E-13	18,9260845
GO:0006397	mRNA processing	34/300	9,88982E-13	5,85328523
GO:0022613	Ribosome biogenesis and translation	17/60	1,0215E-12	17,6029521
GO:0000387	mRNA processing	13/38	8,55351E-11	22,9647059
GO:0016071	mRNA processing	18/95	2,94294E-10	10,4140985
GO:0006415	Ribosome biogenesis and translation	17/96	3,50141E-09	9,56366684
GO:0042255	Ribosome biogenesis and translation	13/50	3,96262E-09	15,5071542
GO:0070125	Ribosome biogenesis and translation	16/89	9,35166E-09	9,72172122
GO:0070126	Ribosome biogenesis and translation	16/89	9,35166E-09	9,72172122
GO:0006414	Ribosome biogenesis and translation	17/104	1,15681E-08	8,68068021
GO:0032543	Ribosome biogenesis and translation	17/105	1,31423E-08	8,58159506
GO:0042776	Aerobic metabolism	8/17	7,71935E-08	38,8486204
GO:0015986	Aerobic metabolism	8/19	2,24512E-07	31,7819809
GO:0007007	Aerobic metabolism	11/49	5,35111E-07	12,717935
GO:0000028	Ribosome biogenesis and translation	7/17	2,50581E-06	30,5234375
GO:0071276		8/27	5,20816E-06	18,3925586
GO:0042407		8/28	6,97697E-06	17,4720358
GO:0006396	mRNA processing	18/179	7,34177E-06	4,9591654
GO:0046686		8/29	9,01497E-06	16,6391818
GO:0008380	mRNA processing	13/98	1,58326E-05	6,73356401
GO:0006417	Ribosome biogenesis and translation	17/178	3,16476E-05	4,67296293
GO:0006283	DNA damage and repair	11/73	3,31929E-05	7,78527318
GO:0030490	Ribosome biogenesis and translation	8/35	4,01977E-05	12,9376087
GO:0006123	Aerobic metabolism	6/17	5,70694E-05	23,7303098
GO:0006296	DNA damage and repair	8/37	6,08266E-05	12,0441256

GO:0071294		6/18	8,06708E-05	21,7516704
GO:0033683	DNA damage and repair	8/39	8,95584E-05	11,2659306
GO:0006913		8/40	0,000107668	10,913311
GO:0071280		6/19	0,00010922	20,077437
GO:0015985	Aerobic metabolism	5/12	0,000153986	31,0126984
GO:0006122	Aerobic metabolism	5/12	0,000153986	31,0126984
GO:0000245	mRNA processing	8/46	0,000301417	9,18733074
GO:0046688		6/23	0,000350193	15,35019
GO:0000291	mRNA processing	7/35	0,000410836	10,891183
GO:0007005	Aerobic metabolism	15/175	0,000410836	4,1303267
GO:0000027	Ribosome biogenesis and translation	6/25	0,000562036	13,7329739
GO:0045727	Ribosome biogenesis and translation	11/100	0,000577522	5,41593279
GO:0000470	Ribosome biogenesis and translation	6/26	0,000690539	13,045657
GO:0006259	DNA damage and repair	19/277	0,000690539	3,25770749
GO:0065004	DNA damage and repair	13/143	0,00077357	4,39253394
GO:0006289	DNA damage and repair	11/105	0,000862069	5,12653345
GO:0043618	Cellular response to oxidative stress	10/87	0,000875936	5,6815993
GO:0006626	Aerobic metabolism	8/55	0,000985815	7,42462754
GO:0006882		6/28	0,000997567	11,8584734
GO:0006261		12/129	0,001154234	4,49800313
GO:0010557	Ribosome biogenesis and translation	12/129	0,001154234	4,49800313
GO:0000380	mRNA processing	5/18	0,001157204	16,6940171
GO:0010043		6/29	0,001166192	11,3423066
GO:0071248		12/131	0,001289341	4,42195117
GO:0055069		6/30	0,001391799	10,8691537
GO:0051170		9/77	0,001695826	5,77990636
GO:0031328	Ribosome biogenesis and translation	14/180	0,001819828	3,70606234
GO:0043928	mRNA processing	6/33	0,002356224	9,65998515
GO:0036388		8/64	0,002579229	6,22850751
GO:0072655	Aerobic metabolism	7/48	0,002579229	7,43292683
GO:0043488	mRNA processing	12/146	0,003365241	3,9239244
GO:0034599	Cellular response to oxidative stress	11/125	0,003440635	4,22279516
GO:0032508		7/51	0,003686346	6,92507102
GO:0051276		10/106	0,003791468	4,55266854
GO:0010629		19/322	0,003896971	2,76741757
GO:0006297	DNA damage and repair	5/24	0,004294047	11,4187135
GO:0006353	mRNA processing	8/70	0,004437895	5,62401674
GO:0090304		8/71	0,004847781	5,53446255
GO:0017148		9/90	0,004888466	4,8490284
GO:0042773	Aerobic metabolism	3/6	0,00514706	43,2345133
GO:0016070	mRNA processing	11/133	0,005364652	3,94426599
GO:0006839	Aerobic metabolism	7/56	0,005977414	6,21683673
GO:0000288		7/56	0,005977414	6,21683673
GO:0051444		4/15	0,006554483	15,7500504
GO:0061418	Cellular response to oxidative stress	8/75	0,006579733	5,2029784
GO:0006479		6/41	0,006665287	7,44893414
GO:0140053	Aerobic metabolism	6/42	0,00754991	7,24164811
GO:0000056	Ribosome biogenesis and translation	3/7	0,00813233	32,4242257
GO:0002082	Aerobic metabolism	4/16	0,008149442	14,4368071
GO:0006294		5/29	0,00939057	9,0375
GO:0006402		6/44	0,00939057	6,85980542
GO:0061013		10/122	0,009793111	3,89907705

GO:0061641	5/30	0,010539311	8,67555556
GO:0034080	5/30	0,010539311	8,67555556
GO:0000462	5/30	0,010539311	8,67555556
GO:0048524	7/63	0,010915957	5,43777902
GO:0006605	10/125	0,011215376	3,79677577
GO:0006301	6/46	0,011215376	6,51614699
GO:0061043	3/8	0,011501219	25,9380531
GO:0045926	10/126	0,0117148	3,76385122
GO:0006271	4/18	0,011787565	12,3731391
GO:0031145	8/84	0,011968733	4,58471683
GO:0051168	8/84	0,011968733	4,58471683
GO:0031571	7/65	0,012284463	5,2497306
GO:0042127	32/764	0,012955708	1,94427005
GO:0031055	5/32	0,012986704	8,03209877
GO:0010628	23/482	0,013018855	2,21384047
GO:0034249	8/86	0,01341128	4,46670108
GO:0033119	4/19	0,013678501	11,5476718
GO:0007346	12/178	0,013906121	3,16228345
GO:0045069	7/67	0,013906121	5,07421875
GO:0006401	6/49	0,013996086	6,06059978
GO:0071456	10/131	0,014188054	3,60739159
GO:1904667	3/9	0,014790032	21,6139381
GO:0018198	3/9	0,014790032	21,6139381
GO:0042981	31/742	0,014865108	1,93672876
GO:0006369	5/34	0,015790268	7,47739464
GO:0009060	4/21	0,018650603	10,1880788
GO:0042276	4/21	0,018650603	10,1880788
GO:1904666	4/21	0,018650603	10,1880788
GO:0031125	3/10	0,019671295	18,5252845
GO:0043632	3/10	0,019671295	18,5252845
GO:0070498	8/94	0,02116932	4,04952916
GO:0032269	8/94	0,02116932	4,04952916
GO:0070987	4/22	0,02124517	9,62158167
GO:0000717	4/22	0,02124517	9,62158167
GO:1901798	4/22	0,02124517	9,62158167
GO:0034508	5/37	0,021527218	6,77534722
GO:0080135	9/118	0,022550025	3,59822273
GO:2001022	6/55	0,022550025	5,31684923
GO:0046916	8/96	0,023042533	3,95708766
GO:0090305	7/75	0,023156033	4,4754136
GO:0043486	5/38	0,023157389	6,56969697
GO:1901799	5/38	0,023157389	6,56969697
GO:2000059	5/38	0,023157389	6,56969697
GO:0006977	6/56	0,023761214	5,21024499
GO:0050658	7/76	0,024164972	4,41032609
GO:0006336	5/39	0,025545082	6,37614379
GO:0050679	9/123	0,028224223	3,43952089
GO:0062197	8/101	0,02992785	3,7433788
GO:0000727	3/12	0,030475311	14,4070796
GO:0019941	12/201	0,030989655	2,77415887
GO:0046782	5/41	0,030989655	6,0212963
GO:0045333	5/42	0,034085072	5,85825826

GO:0019985	5/42	0,034085072	5,85825826
GO:0051085	4/26	0,034821712	7,87059061
GO:0070911	4/26	0,034821712	7,87059061
GO:1903051	4/26	0,034821712	7,87059061
GO:0050434	4/26	0,034821712	7,87059061
GO:0000731	5/43	0,035865402	5,70380117
GO:0045116	3/13	0,035865402	12,965708
GO:0006576	3/13	0,035865402	12,965708
GO:0000054	3/13	0,035865402	12,965708
GO:0060213	3/13	0,035865402	12,965708
GO:0071347	10/155	0,036999189	3,00658659
GO:0051092	10/155	0,036999189	3,00658659
GO:0034614	6/63	0,038054934	4,56874927
GO:0071229	4/28	0,043291308	7,21396896
GO:0008334	4/28	0,043291308	7,21396896
GO:0071345	21/482	0,044439157	2,00307886
GO:0035973	4/29	0,048468011	6,92505543
GO:0045070	4/29	0,048468011	6,92505543
