# Supplementary material for: Spatial resolution of cellular senescence dynamics in human colorectal liver metastasis
Source: Aging Cell. 2023 May 8;22(7):e13853. doi: 10.1111/acel.13853 (PMC10352575; doi:10.1111/acel.13853)
Supplement: Supplementary file 11 — Table S5 [file ACEL-22-e13853-s007.pdf]

Supplementary TableS5 mSMCCs pathways

Term	Pathway	Overlap	Adjusted.P.val	Odds.Ratio
GO:0030198	ECM remodelling	40/300	2,2395E-23	11,8730159
GO:0097435	ECM remodelling	38/351	4,4863E-19	9,27033282
GO:0043062	ECM remodelling	29/216	7,0965E-17	11,5107257
GO:0045229	ECM remodelling	29/217	7,0965E-17	11,4489119
GO:0002576		22/125	4,0446E-15	15,5091694
GO:0043312	Regulation of immune res	38/481	8,1368E-15	6,5060166
GO:0002283	Regulation of immune res	38/485	9,1971E-15	6,44645845
GO:0002446	Regulation of immune res	38/488	9,8847E-15	6,40248469
GO:0030199	ECM remodelling	18/89	1,7598E-13	18,1693225
GO:0045055		23/180	5,3103E-13	10,6474321
GO:0030334	Regulation of cell migratio	28/408	2,8197E-09	5,39457735
GO:0044267		26/417	1,1577E-07	4,82897141
GO:0043687		23/345	2,9452E-07	5,14763675
GO:1990000		11/63	2,9452E-07	14,797153
GO:0019221	Citokine signaling	31/621	4,4284E-07	3,84867849
GO:0006898		15/143	4,5011E-07	8,28350632
GO:0030336	Regulation of cell migratio	15/144	4,6676E-07	8,21887331
GO:0010466		10/63	3,3391E-06	13,1506758
GO:0002480	Regulation of immune res	5/8	4,4968E-06	114,430894
GO:0070527		8/36	4,9102E-06	19,7987928
GO:0042981	Inhibition of apoptosis	32/742	5,5411E-06	3,29326111
GO:0043069	Inhibition of apoptosis	22/381	5,5411E-06	4,39160219
GO:0060333	Citokine signaling	10/68	5,5749E-06	12,0139398
GO:0043066	Inhibition of apoptosis	25/485	5,5992E-06	3,91792868
GO:0030335	Regulation of cell migratio	18/269	9,4133E-06	5,09241865
GO:0007229	Integrin mediated signalir	10/75	1,285E-05	10,7163121
GO:0075294		5/10	1,3405E-05	68,6515679
GO:0046598		5/10	1,3405E-05	68,6515679
GO:0034109	Regulation of cell migratio	8/44	1,7483E-05	15,3928013
GO:0071346	Citokine signaling	12/121	1,7483E-05	7,70602883
GO:1905049	ECM remodelling	4/5	1,751E-05	273,708333
GO:0007160	Regulation of cell migratio	11/100	1,8374E-05	8,62925347
GO:0030042	Regulation of cell migratio	5/11	2,0604E-05	57,2067364
GO:0002474	Regulation of immune res	7/33	2,9464E-05	18,5929825
GO:0031581	Regulation of cell migratio	5/12	3,2905E-05	49,0318566
GO:0007044	Regulation of cell migratio	7/34	3,4607E-05	17,9034438
GO:0007179	TGF-β signaling	10/89	4,628E-05	8,81093455
GO:0010811	Regulation of cell migratio	9/70	5,1594E-05	10,2428894
GO:2000147	Regulation of cell migratio	15/221	6,0165E-05	5,12652904
GO:0044319	Regulation of cell migratio	6/24	6,1212E-05	22,9487179
GO:0002483	Regulation of immune res	5/14	6,9321E-05	38,132017
GO:0046596		7/39	7,9676E-05	15,102193
GO:0002484	Regulation of immune res	4/7	8,2504E-05	91,2268519
GO:0002486	Regulation of immune res	4/7	8,2504E-05	91,2268519
GO:1905048	ECM remodelling	4/7	8,2504E-05	91,2268519
GO:0090505	ECM remodelling	5/16	0,0001298	31,1957555
GO:0007015		12/152	0,0001298	5,99020408
GO:0010951		8/63	0,00018613	10,065557
GO:0034375	Lipid metabolism	5/18	0,00023661	26,3937282
GO:0048522		25/625	0,00028124	2,98189763

GO:0071560	TGF-β signaling	10/114	0,00031988	6,68439716
GO:2000146	Regulation of cell migratio	10/114	0,00031988	6,68439716
GO:0031330	Lipid metabolism	8/69	0,00033741	9,07273147
GO:0022607		15/261	0,00033751	4,28414194
GO:0008154	Regulation of cell migratio	7/50	0,00034446	11,2325581
GO:0014910		5/20	0,00036577	22,8722416
GO:0034378	Lipid metabolism	4/10	0,00036759	45,6064815
GO:1900024	Regulation of cell migratio	7/51	0,00036759	10,9767145
GO:0032233	Regulation of cell migratio	7/51	0,00036759	10,9767145
GO:0050768		6/35	0,00042998	14,2360743
GO:0006024	ECM remodelling	9/97	0,00048543	7,09042724
GO:1900026	Regulation of cell migratio	6/36	0,00048543	13,7608392
GO:0002711	Regulation of immune res	6/36	0,00048543	13,7608392
GO:0031032	Regulation of cell migratio	8/76	0,00057691	8,13587407
GO:0006027	ECM remodelling	7/56	0,00062802	9,85413534
GO:0033275		6/38	0,00063128	12,8994755
GO:0030049		6/38	0,00063128	12,8994755
GO:0034379	Lipid metabolism	4/12	0,00070863	34,2013889
GO:0033700	Lipid metabolism	4/12	0,00070863	34,2013889
GO:0006936		10/129	0,00070863	5,83735622
GO:1903902		5/24	0,00074963	18,0533651
GO:0043588		8/80	0,00074963	7,68231612
GO:0030155	Regulation of cell migratio	10/133	0,00087373	5,64637029
GO:0007178		10/133	0,00087373	5,64637029
GO:0006639	Lipid metabolism	6/41	0,00087373	11,792008
GO:0022408	Regulation of cell migratio	6/41	0,00087373	11,792008
GO:0042177		6/42	0,00099388	11,4638695
GO:0001916	Regulation of immune res	5/26	0,00103698	16,3323378
GO:0045861		6/43	0,00111212	11,1534682
GO:0001932		14/266	0,00119262	3,88808953
GO:0001569		4/14	0,00119262	27,3583333
GO:0014912		4/14	0,00119262	27,3583333
GO:0016101	Lipid metabolism	7/64	0,00119262	8,46765159
GO:0051496		6/44	0,00119635	10,8594038
GO:0045765		12/203	0,00134047	4,37928197
GO:0051261		5/28	0,00136011	14,9106196
GO:0007043	Regulation of cell migratio	7/66	0,00136011	8,17977996
GO:0022411	ECM remodelling	7/66	0,00136011	8,17977996
GO:0022617	ECM remodelling	7/66	0,00136011	8,17977996
GO:0051050		8/91	0,00153102	6,6604446
GO:0001914	Regulation of immune res	5/29	0,00154816	14,2886179
GO:0002501	Regulation of immune res	3/6	0,00158182	68,183391
GO:0042270	Regulation of immune res	3/6	0,00158182	68,183391
GO:0001523		8/92	0,00158542	6,58081824
GO:0050821		11/179	0,00170461	4,55304186
GO:0050776	Regulation of immune res	11/179	0,00170461	4,55304186
GO:0007010		9/120	0,00170461	5,61464999
GO:0048844		5/30	0,00170461	13,7163763
GO:0052548		7/71	0,00196227	7,53881579
GO:0010770		6/50	0,00210227	9,37571519
GO:0071345		19/482	0,00221118	2,89286308
GO:0010628		19/482	0,00221118	2,89286308

GO:0002479	7/73	0,00221118	7,30962254
GO:0035023	7/73	0,00221118	7,30962254
GO:0007162	7/73	0,00221118	7,30962254
GO:0051492	7/74	0,00231506	7,20015711
GO:0034382	3/7	0,00231506	51,1349481
GO:0038063	3/7	0,00231506	51,1349481
GO:0043589	3/7	0,00231506	51,1349481
GO:0051045	3/7	0,00231506	51,1349481
GO:0030855	8/101	0,0026035	5,94123883
GO:0002697	6/53	0,00261425	8,7759262
GO:0043067	11/194	0,00301416	4,17663302
GO:0042590	7/78	0,00307671	6,79313071
GO:2000181	7/78	0,00307671	6,79313071
GO:2000145	9/133	0,0031766	5,02268323
GO:0031325	8/105	0,0032409	5,69507768
GO:0032489	3/8	0,00338696	40,9058824
GO:0038089	3/8	0,00338696	40,9058824
GO:0045785	7/80	0,00345859	6,60634463
GO:0046635	4/20	0,00364282	17,09375
GO:0051240	15/345	0,00364282	3,17984903
GO:0010977	6/58	0,00394861	7,93006993
GO:0030206	4/21	0,00435637	16,0874183
GO:0007156	6/60	0,00460034	7,63558664
GO:0006464	30/1025	0,00460034	2,15347731
GO:0034371	3/9	0,00460034	34,0865052
GO:0038065	3/9	0,00460034	34,0865052
GO:0042157	3/9	0,00460034	34,0865052
GO:0010873	3/9	0,00460034	34,0865052
GO:0034249	7/86	0,00490741	6,10273151
GO:0010595	7/86	0,00490741	6,10273151
GO:0051043	4/22	0,00490741	15,1929012
GO:0044273	5/40	0,00507167	9,79243405
GO:0030195	5/40	0,00507167	9,79243405
GO:0016525	7/87	0,00514732	6,02614035
GO:0006417	10/178	0,00535986	4,1244512
GO:0031532	6/63	0,00555895	7,2326095
GO:0031401	11/214	0,00573638	3,76128535
GO:0010594	7/89	0,00575201	5,87856226
GO:0034333	3/10	0,00599383	29,2155215
GO:0071407	9/150	0,00633551	4,4132772
GO:0034097	9/150	0,00633551	4,4132772
GO:0032956	7/91	0,00634639	5,7380117
GO:0042127	24/764	0,00634639	2,29544171
GO:0033344	4/24	0,00634639	13,6722222
GO:0001912	5/43	0,00657484	9,01797176
GO:0048010	6/67	0,00723174	6,75696435
GO:0001954	5/44	0,00723174	8,78629501
GO:0034114	4/25	0,0072698	13,0205026
GO:0032269	7/94	0,00739856	5,53930228
GO:0010634	7/94	0,00739856	5,53930228
GO:0048261	4/26	0,00832701	12,4280303
GO:0001501	9/158	0,00859639	4,17461522

GO:0022409	5/47	0,00935913	8,15745811
GO:0050650	4/27	0,00935913	11,8870773
GO:0010872	3/12	0,00935913	22,7208766
GO:0030208	3/12	0,00935913	22,7208766
GO:0090257	3/12	0,00935913	22,7208766
GO:0010896	3/12	0,00935913	22,7208766
GO:0045916	3/12	0,00935913	22,7208766
GO:0045717	3/12	0,00935913	22,7208766
GO:0009966	10/198	0,01027292	3,68190735
GO:0032970	6/73	0,01027292	6,14998434
GO:0006937	4/28	0,01027292	11,3912037
GO:0050771	4/28	0,01027292	11,3912037
GO:1905477	7/101	0,0103784	5,124972
GO:0034332	5/49	0,01061278	7,78587266
GO:0002478	7/103	0,01104867	5,01769006
GO:0071417	7/103	0,01104867	5,01769006
GO:0030207	3/13	0,01104867	20,4477509
GO:0030205	3/13	0,01104867	20,4477509
GO:0034370	3/13	0,01104867	20,4477509
GO:0032370	3/13	0,01104867	20,4477509
GO:0045940	3/13	0,01104867	20,4477509
GO:0042304	4/29	0,01104867	10,935
GO:0006096	4/29	0,01104867	10,935
GO:0030510	6/76	0,01184061	5,88551449
GO:0050770	5/51	0,01198271	7,446599
GO:0030030	4/30	0,01225703	10,5138889
GO:0030204	4/30	0,01225703	10,5138889
GO:0035633	4/30	0,01225703	10,5138889
GO:2001044	3/14	0,01326954	18,5879207
GO:0016045	3/14	0,01326954	18,5879207
GO:1901163	3/14	0,01326954	18,5879207
GO:0090092	4/31	0,01354069	10,1239712
GO:0035025	4/31	0,01354069	10,1239712
GO:1903076	6/80	0,0146802	5,56624457
GO:0051893	5/54	0,01474509	6,98961815
GO:1902533	18/546	0,0151779	2,38636364
GO:0006090	5/55	0,0151779	6,84947735
GO:0043535	5/55	0,0151779	6,84947735
GO:0006641	5/55	0,0151779	6,84947735
GO:0010715	3/15	0,0151779	17,0380623
GO:1903053	3/15	0,0151779	17,0380623
GO:0030038	3/15	0,0151779	17,0380623
GO:0050651	3/15	0,0151779	17,0380623
GO:0043149	3/15	0,0151779	17,0380623
GO:2001028	3/15	0,0151779	17,0380623
GO:0034116	3/15	0,0151779	17,0380623
GO:0042327	11/253	0,015836	3,14881915
GO:0051017	4/33	0,01593316	9,42480843
GO:0007266	5/56	0,01619354	6,71483227
GO:0044272	7/113	0,01643806	4,54200596
GO:0001819	13/335	0,01643806	2,80524945
GO:1902003	4/34	0,01750567	9,11018519

GO:0045953	3/16	0,01785482	15,7266436
GO:0046464	4/35	0,01925944	8,81586022
GO:0071526	4/35	0,01925944	8,81586022
GO:0015914	5/59	0,01980119	6,34081817
GO:0043122	10/224	0,02036578	3,23026447
GO:0048523	18/566	0,02036578	2,2968725
GO:0035909	3/17	0,02036578	14,6025704
GO:0031341	3/17	0,02036578	14,6025704
GO:0090287	3/17	0,02036578	14,6025704
GO:0043691	3/17	0,02036578	14,6025704
GO:1902430	3/17	0,02036578	14,6025704
GO:0045922	3/17	0,02036578	14,6025704
GO:0001895	4/36	0,0204427	8,53993056
GO:0048585	5/61	0,02184744	6,11373818
GO:0007417	11/268	0,02184744	2,96275115
GO:0072524	2/5	0,02184744	45,2988506
GO:2000266	2/5	0,02184744	45,2988506
GO:0071681	2/5	0,02184744	45,2988506
GO:0032803	2/5	0,02184744	45,2988506
GO:0046351	2/5	0,02184744	45,2988506
GO:1903729	2/5	0,02184744	45,2988506
GO:0032371	2/5	0,02184744	45,2988506
GO:0071680	2/5	0,02184744	45,2988506
GO:2000267	2/5	0,02184744	45,2988506
GO:0046461	2/5	0,02184744	45,2988506
GO:0032831	2/5	0,02184744	45,2988506
GO:0038033	2/5	0,02184744	45,2988506
GO:0050995	3/18	0,02252357	13,6283737
GO:1902905	6/91	0,02280442	4,8431921
GO:0043542	4/39	0,02537549	7,80674603
GO:0061097	4/39	0,02537549	7,80674603
GO:0048259	4/39	0,02537549	7,80674603
GO:0043567	3/19	0,02537549	12,7759516
GO:1900746	3/19	0,02537549	12,7759516
GO:0046503	3/19	0,02537549	12,7759516
GO:1902992	3/19	0,02537549	12,7759516
GO:1903364	3/19	0,02537549	12,7759516
GO:0051894	3/19	0,02537549	12,7759516
GO:0001952	5/65	0,0260118	5,70499419
GO:0007169	14/404	0,0260118	2,49448441
GO:0070372	10/238	0,02709294	3,02973746
GO:1904375	3/20	0,02886313	12,0238144
GO:0016052	4/41	0,02886313	7,38400901
GO:0030865	4/41	0,02886313	7,38400901
GO:0007411	9/203	0,02886313	3,19889986
GO:0060312	2/6	0,02886313	33,9724138
GO:1904026	2/6	0,02886313	33,9724138
GO:0045622	2/6	0,02886313	33,9724138
GO:0010901	2/6	0,02886313	33,9724138
GO:0048845	2/6	0,02886313	33,9724138
GO:0051005	2/6	0,02886313	33,9724138
GO:0010985	2/6	0,02886313	33,9724138

GO:0090209	2/6	0,02886313	33,9724138
GO:1901164	2/6	0,02886313	33,9724138
GO:0060563	2/6	0,02886313	33,9724138
GO:1900122	2/6	0,02886313	33,9724138
GO:0043408	8/166	0,02910351	3,48546978
GO:0010718	4/42	0,03009292	7,18932749
GO:0036294	5/69	0,03103435	5,34734321
GO:2000209	3/21	0,03103435	11,355248
GO:0051004	3/21	0,03103435	11,355248
GO:2000242	3/21	0,03103435	11,355248
GO:0045727	6/100	0,03195656	4,37747359
GO:0010810	4/43	0,03195656	7,00462963
GO:0021762	4/43	0,03195656	7,00462963
GO:0048754	4/44	0,03414838	6,82916667
GO:0003208	4/44	0,03414838	6,82916667
GO:0009891	4/44	0,03414838	6,82916667
GO:0048260	4/44	0,03414838	6,82916667
GO:0032270	6/102	0,03414838	4,28583916
GO:0030031	3/22	0,03414838	10,757057
GO:0060759	3/22	0,03414838	10,757057
GO:0051055	3/22	0,03414838	10,757057
GO:0034330	5/72	0,03535283	5,10712986
GO:0000904	5/72	0,03535283	5,10712986
GO:0050769	5/72	0,03535283	5,10712986
GO:0060828	10/253	0,03566377	2,84052768
GO:1900424	2/7	0,03566377	27,1765517
GO:0006600	2/7	0,03566377	27,1765517
GO:0051660	2/7	0,03566377	27,1765517
GO:0052200	2/7	0,03566377	27,1765517
GO:0003376	2/7	0,03566377	27,1765517
GO:0097084	2/7	0,03566377	27,1765517
GO:0003174	2/7	0,03566377	27,1765517
GO:0052031	2/7	0,03566377	27,1765517
GO:1900223	2/7	0,03566377	27,1765517
GO:0062043	2/7	0,03566377	27,1765517
GO:2001046	2/7	0,03566377	27,1765517
GO:0008284	15/474	0,03622512	2,27094689
GO:0010875	3/23	0,03661943	10,2186851
GO:0048145	4/46	0,03713896	6,50330688
GO:0035722	4/46	0,03713896	6,50330688
GO:0051094	8/177	0,03772341	3,2567714
GO:0050778	5/75	0,0395725	4,88750622
GO:0033365	7/142	0,04005055	3,56103964
GO:0008361	3/24	0,04005055	9,73158675
GO:0061621	3/24	0,04005055	9,73158675
GO:0061718	3/24	0,04005055	9,73158675
GO:0061099	3/24	0,04005055	9,73158675
GO:0150117	3/24	0,04005055	9,73158675
GO:1902531	14/437	0,04161399	2,29595047
GO:0071349	4/48	0,04161399	6,20707071
GO:0034446	4/48	0,04161399	6,20707071
GO:0045807	4/48	0,04161399	6,20707071

GO:0032760	5/77	0,04195938	4,75125823
GO:0055090	3/25	0,04195938	9,28877005
GO:0032374	3/25	0,04195938	9,28877005
GO:0061620	3/25	0,04195938	9,28877005
GO:0030837	3/25	0,04195938	9,28877005
GO:0045806	3/25	0,04195938	9,28877005
GO:0046631	2/8	0,04195938	22,645977
GO:0019885	2/8	0,04195938	22,645977
GO:2000514	2/8	0,04195938	22,645977
GO:2000564	2/8	0,04195938	22,645977
GO:0061387	2/8	0,04195938	22,645977
GO:1902965	2/8	0,04195938	22,645977
GO:0038098	2/8	0,04195938	22,645977
GO:1900272	2/8	0,04195938	22,645977
GO:1905247	2/8	0,04195938	22,645977
GO:0043372	2/8	0,04195938	22,645977
GO:1900426	2/8	0,04195938	22,645977
GO:1902966	2/8	0,04195938	22,645977
GO:1905668	2/8	0,04195938	22,645977
GO:0031623	4/49	0,04195938	6,06882716
GO:0009895	4/49	0,04195938	6,06882716
GO:0030111	6/111	0,04309253	3,91668332
GO:0051270	4/50	0,04468683	5,9365942
GO:0030449	4/50	0,04468683	5,9365942
GO:0009968	10/267	0,04468683	2,68385904
GO:0043065	11/310	0,04470806	2,54107999
GO:1901016	3/26	0,04572382	8,88445915
GO:1901222	5/80	0,04599732	4,56051103
GO:0051129	5/80	0,04599732	4,56051103
GO:0051345	7/149	0,04610512	3,38428466
GO:0030301	4/51	0,04696123	5,80998818
GO:0051130	6/114	0,04752925	3,80730381
GO:0034250	5/81	0,04774773	4,50027508
GO:1903557	5/81	0,04774773	4,50027508
GO:0043086	3/27	0,04811031	8,51384083
GO:0051272	3/27	0,04811031	8,51384083
GO:0030514	4/52	0,04811031	5,68865741
GO:1904377	4/52	0,04811031	5,68865741
GO:1903078	4/52	0,04811031	5,68865741
GO:0048013	5/82	0,04811031	4,44160369
GO:0001933	7/152	0,04811031	3,31375681
GO:0036500	2/9	0,04811031	19,4098522
GO:1902959	2/9	0,04811031	19,4098522
GO:2001185	2/9	0,04811031	19,4098522
GO:0006958	2/9	0,04811031	19,4098522
GO:0033599	2/9	0,04811031	19,4098522
GO:0072182	2/9	0,04811031	19,4098522
GO:0022898	2/9	0,04811031	19,4098522
GO:0048333	2/9	0,04811031	19,4098522
GO:0034372	2/9	0,04811031	19,4098522
GO:0003183	2/9	0,04811031	19,4098522
GO:0060192	2/9	0,04811031	19,4098522

GO:2000258	2/9	0,04811031	19,4098522
GO:2001187	2/9	0,04811031	19,4098522
GO:0043382	2/9	0,04811031	19,4098522
GO:0090037	2/9	0,04811031	19,4098522
GO:0045766	6/116	0,04811031	3,73769866
